# Nanopore direct RNA sequencing and the epitranscriptome: Advances in mapping native RNA landscapes

**DOI:** 10.1002/imt2.70136

**Published:** 2026-07-01

**Authors:** Tianyuan Zhang, Jia Li, Chao Tang, You Wu, Hao Wu, Xi‐Tong Zhu, Ziyang Luo, Hang Qin, Lishan Ding, Yu Zeng, Shiou Yih Lee, Xiaotao Shen, Shiwen Gao, Zhaoyang Tian, Qian Tang, Mian Li, Muhammad Tahir Ul Qamar, Yang Dong, Komivi Dossa, Yaxuan Zhang, Hu Chen, Sanqi An, Xiang Yu, Lu Chen, Dingjie Wang, Shengli Li, Ling‐Ling Chen, Yanqiang Li

**Affiliations:** ^1^ Department of Urology The First Affiliated Hospital of Xi'an Jiaotong University Xi'an China; ^2^ Benagen Institute Wuhan China; ^3^ School of Mathematics and Statistics Wuhan University Wuhan China; ^4^ Key Laboratory of Birth Defects and Related Diseases of Women and Children, Ministry of Education, State Key Laboratory of Biotherapy, Department of Laboratory Medicine West China Second University Hospital Sichuan University Chengdu China; ^5^ School of Basic Medical Sciences and Forensic Medicine, North Sichuan Medical College, Institute of Basic Medicine Nanchong China; ^6^ School of Life Sciences and Biotechnology Shanghai Jiao Tong University Shanghai China; ^7^ Precision Research Center for Refractory Diseases, Shanghai Jiao Tong University Pioneer Research Institute for Molecular and Cell Therapies, Shanghai General Hospital, Shanghai Jiao Tong University School of Medicine Shanghai China; ^8^ College of Life Science and Technology Guangxi University Nanning China; ^9^ Guangxi Key Laboratory of Organ Donation and Transplantation, Guangxi Key Laboratory of AIDS Prevention and Treatment Institute of Transplant Medicine, The Second Affiliated Hospital of Guangxi Medical University, Guangxi Clinical Research Center for Organ Transplantation Guangxi Medical University Nanning China; ^10^ School of Pharmacy Chengdu University of Traditional Chinese Medicine Chengdu China; ^11^ Institute of Herbgenomics Chengdu University of Traditional Chinese Medicine Chengdu China; ^12^ Faculty of Health and Life Sciences INTI International University Nilai Negeri Sembilan Malaysia; ^13^ Lee Kong Chian School of Medicine Nanyang Technological University Singapore Singapore; ^14^ School of Chemistry, Chemical Bioneering, and Biotechnology Nanyang Technological University Singapore Singapore; ^15^ Sailgene Technology Co., Limited Hong Kong China; ^16^ Biomedical Sciences College & Shandong Medicinal Biotechnology Centre, Shandong First Medical University & Shandong Academy of Medical Sciences Jinan China; ^17^ Department of Bioinformatics and Biotechnology Government College University Faisalabad Pakistan; ^18^ CIRAD, UMR AGAP Institut Guadeloupe Petit Bourg France; ^19^ UMR AGAP Institut, CIRAD, INRAE, Univ Montpellier, Institut Agro Montpellier France; ^20^ Institute for Math & AI Wuhan University Wuhan China; ^21^ Multi‐omics Platform for Agricultural Organisms Yazhouwan National Laboratory Sanya China

**Keywords:** direct RNA sequencing, long‐read transcriptome, nanopore sequencing, poly(A) tail, RNA modification, SQK‐RNA004, human medicine

## Abstract

Nanopore direct RNA sequencing (DRS) has transformed transcriptomics by enabling single‐molecule, long‐read sequencing of native RNA without the need for reverse transcription or amplification. In contrast to short‐read RNA‐seq and cDNA‐based long‐read approaches, DRS can simultaneously capture multiple RNA modifications, full‐length transcript architecture, alternative splicing patterns, and poly(A) tail features within individual molecules, thereby providing an integrated view of transcriptomic and epitranscriptomic regulation. In this comprehensive review, we outline the biophysical principles underlying nanopore DRS and trace its technological evolution. We compare its performance with short‐read RNA sequencing, long‐read cDNA sequencing, and conventional RNA‐modification mapping strategies, highlighting its advantages in isoform‐resolved quantification and multilayer RNA feature integration, while also clarifying contexts in which alternative or combined approaches may be more appropriate for robust biological interpretation. We further summarize optimized experimental workflows, including library construction strategies tailored to diverse RNA biotypes (mRNA, rRNA, tRNA, circRNA, miRNA, and nonpoly(A) transcripts), as well as recommended quality‐control procedures and sequencing optimization practices. Emphasizing recent computational advances and translational applications of DRS, we cover state‐of‐the‐art algorithms for RNA modification detection, transcript reconstruction, and isoform quantification. We also propose analytical pipelines for poly(A) tail length inference and integrative frameworks that jointly analyze these regulatory layers. We distinguish direct nanopore signals from computational inferences to define confidence levels and emphasize benchmarking and orthogonal validation of readouts. Practical implementation examples are included to facilitate reproducible analysis. Finally, we highlight emerging applications of integrated DRS, including the resolution of complex transcriptomes, the characterization of coordinated epitranscriptomic regulation, and the identification of disease‐associated RNA signatures. We also discuss current technical challenges and future perspectives, particularly in relation to multi‐omics integration and the broader deployment of DRS in precision medicine as well as in plant and animal research.

## Highlights

- DRS enables simultaneous interrogation of multiple features on native RNA molecules at single‐molecule resolution.
- Systematic comparative framework positioning DRS against short‐read, long‐read, and modification‐specific approaches across experimental design, data analysis, and benchmarking.
- A task‐oriented view clarifies when DRS outperforms or complements existing sequencing approaches.
- Evidence‐aware interpretation by distinguishing robust readouts from model‐dependent and validation‐sensitive features of DRS.
- A layered benchmarking and validation framework is outlined to support reliable biological inference from DRS data.

## Graphical abstract

Nanopore direct RNA sequencing advances transcriptomics by capturing full‐length transcripts and multiple RNA modifications; this review details its principles, workflows, tools, applications, challenges, and future research potential.

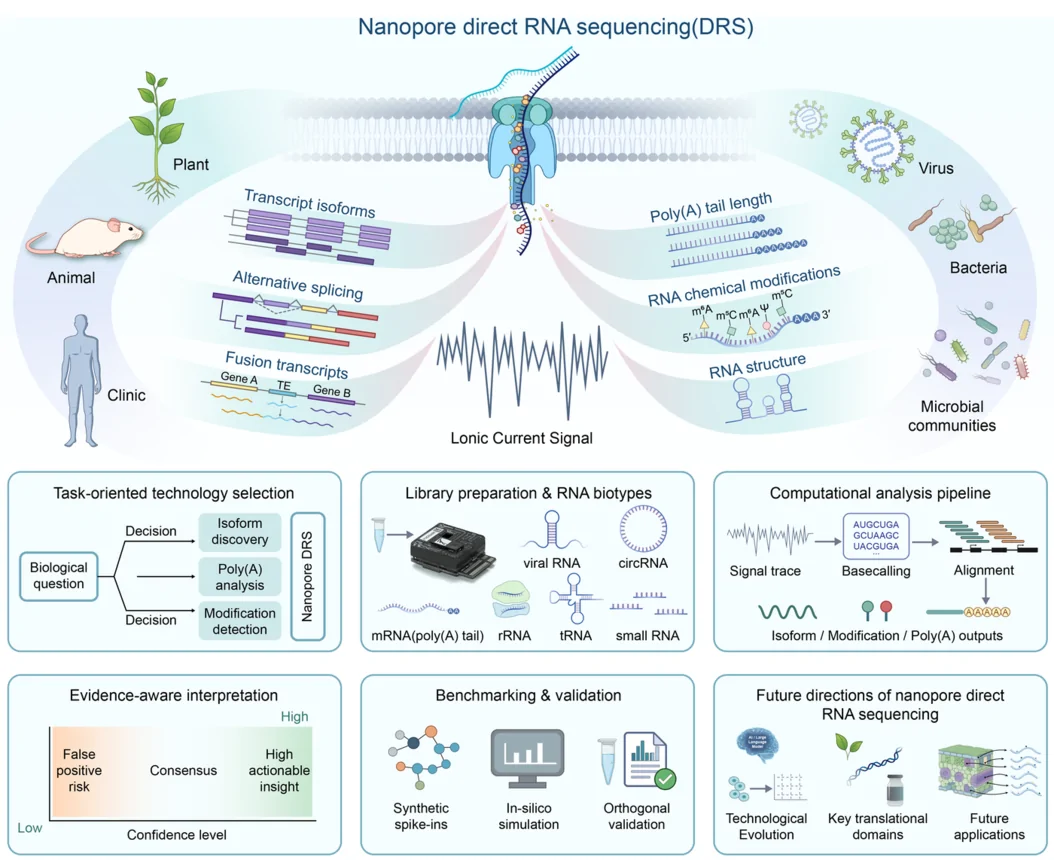

## INTRODUCTION

With the accumulation of experimental and computational evidence, the multilayered and intricate roles of RNA in gene expression control have come into sharp focus. These roles span coding and noncoding RNAs, transcriptional and posttranscriptional regulation, as well as epigenetic modulation and RNA editing. Together, diverse RNA species form a finely tuned and highly dynamic regulatory network that safeguards the spatiotemporal precision and functional versatility of gene expression [1, 2]. In parallel, a growing repertoire of RNA modifications, typified by N^6^‐methyladenosine (m^6^A), has emerged as key epitranscriptomic marks. Their contributions to transcript stability, splicing, nuclear export, translational efficiency, and RNA decay have been progressively elucidated [3, 4]. Analogous to DNA methylation and histone modifications, RNA modifications are highly dynamic and reversible, offering an additional regulatory layer that provides mechanistic insights into the precise control of gene expression programs [5, 6].

**Figure 1:**
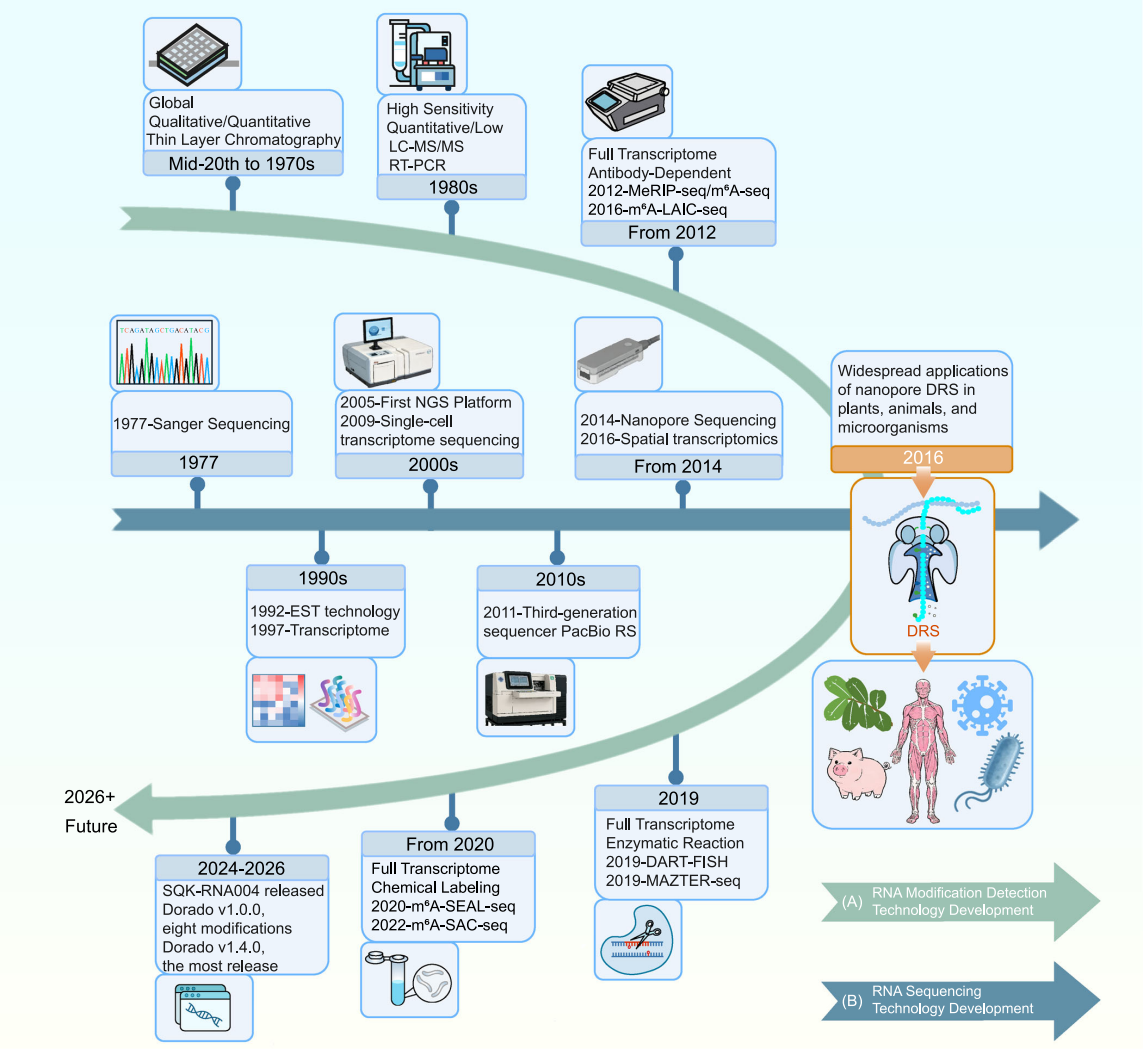
Timeline of major innovations in RNA sequencing and RNA modification technologies. (A) Evolution of RNA modification analysis, from early chromatographic approaches (mid‐20th century), to LC‐MS/MS and RT‐PCR‐based methods (1980s), followed by transcriptome‐wide antibody‐based mapping (e.g., MeRIP‐seq/m^6^A‐seq, m^6^A‐LAIC‐seq) from 2012 onwards, and more recent enzymatic and chemical labeling strategies (e.g., DART‐seq, MAZTER‐seq, m^6^A‐SEAL‐seq, m^6^A‐SAC‐seq). Nanopore DRS with the SQK‐RNA004 chemistry achieves enhanced performance and, when combined with Dorado software v1.0.0 and above, can detect up to eight distinct RNA modifications. (B) Development of RNA sequencing technologies, including Sanger sequencing (1977), next‐generation sequencing (2000s), long‐read platforms (e.g., PacBio RS; 2010s), and nanopore sequencing (from 2014). The introduction of Oxford Nanopore DRS from 2016 is indicated. DART‐seq, deamination adjacent to RNA targeting sequencing; DRS, Direct RNA sequencing. LC‐MS/MS, liquid chromatography coupled with tandem mass spectrometry; m^6^A‐LAIC‐seq, N6‐methyladenosine level and isoform characterization sequencing; m^6^A‐SAC‐seq, N6‐methyladenosine site‐specific alkali cleavage sequencing; m^6^A‐SEAL‐seq, N6‐methyladenosine sequencing via enzymatic‐assisted alkali ligation; m^6^A‐seq, N6‐methyladenosine sequencing; MAZTER‐seq, MazF endonuclease‐assisted RNA m^6^A profiling sequencing; MeRIP‐seq, methylated RNA immunoprecipitation sequencing; PacBio RS, Pacific Biosciences real‐time sequencing; RT‐PCR, reverse‐transcription polymerase chain reaction.

Over the past three decades, RNA sequencing technologies have evolved rapidly, driven by continual breakthroughs in sequencing platforms (Figure 1). This progression has moved from short‐read RNA‐seq (srRNA‐seq) represented by Illumina platforms [7], to long‐read cDNA sequencing represented by PacBio and Oxford Nanopore Technologies (ONT) [8], and, in the last decade, to the advent of nanopore direct RNA sequencing (DRS) [9]. Prior to the next‐generation sequencing (NGS) era, Venter and colleagues in 1991 introduced and implemented the expressed sequence tag (EST) strategy, in which short cDNA fragments were sequenced to rapidly capture gene expression information [10]. Building on this, in 1997, Velculescu, Kinzler, and colleagues coined the term “transcriptome” and used ESTs to systematically catalog cellular transcripts, laying the conceptual foundation for modern transcriptomics [11]. The advent of RNA‐seq marked a paradigm shift, freeing researchers from the limitations of single‐gene, low‐throughput approaches such as Northern blotting and microarrays, and enabling genome‐wide quantification of transcript abundance [12]. Nevertheless, short‐read sequencing is inherently limited by read length and the complexity of transcript reconstruction. In the context of long transcripts, intricate alternative splicing (AS) events, or tandem repetitive regions, assembly‐based inference often fails to recover full‐length structures at single‐molecule resolution [13]. These limitations spurred the development of long‐read cDNA sequencing, in which reverse‐transcribed cDNAs, frequently enriched or amplified by reverse‐transcription PCR (RT‐PCR) or targeted capture, are sequenced in a single‐molecule, enabling isoform‐resolved transcriptome characterization [14].

In parallel with sequencing advances, technologies for detecting and mapping RNA modifications have progressed from global quantification to nucleotide‐level resolution. Early approaches, including paper chromatography, ion‐exchange column chromatography, thin‐layer chromatography (TLC), and dot‐blot assays [15, 16], provided the first evidence for the existence and approximate abundance of major modifications such as m^6^A in eukaryotic mRNA. However, these methods lacked site specificity and sensitivity, limiting their utility for detailed mechanistic studies. Subsequent developments, such as high‐performance liquid chromatography coupled with tandem mass spectrometry (LC‐MS/MS), enabled femtomole‐level quantification, while RT‐PCR combined with Sanger sequencing allowed low‐throughput validation of candidate sites. Nevertheless, LC‐MS/MS does not retain transcript identity or positional information, and RT‐PCR‐based approaches are not scalable to transcriptome‐wide analyses. With the advent of NGS, antibody‐based methods such as m^6^A‐seq and methylated RNA immunoprecipitation sequencing (MeRIP‐seq) [17, 18] generated the first transcriptome‐wide maps of RNA modifications by combining RNA fragmentation with immunoprecipitation. However, these approaches typically localize modifications only to enriched regions of about 100−200 nucleotides (nt), rather than to single bases. Crosslinking‐enhanced strategies such as photo‐crosslinking‐assisted m^6^A sequencing (PA‐m^6^A‐seq) and m^6^A individual‐nucleotide‐resolution cross‐linking and immunoprecipitation (miCLIP) [19, 20], improved resolution to near single‐base level but remain constrained by antibody specificity and potential false positives. Since 2018, non‐antibody‐based strategies have emerged, including chemical derivatization and reverse transcription signature‐based approaches (e.g., Glyoxal and nitrite‐mediated deamination of unmethylated adenosines sequencing (GLORI‐seq) and m^6^A selective chemical labeling sequencing (m^6^A‐SEAL‐seq) [21, 22]), as well as enzyme‐based methods such as enzyme‐based m^6^A detection using MazF (MAZTER‐seq) and deamination adjacent to RNA modification targets sequencing (DART‐seq) [23, 24]. In parallel, related chemistries and workflows have been established for additional marks, including 5‐methylcytosine (m^5^C) and pseudouridine (Ψ). Although these techniques have greatly improved resolution and quantitative comparability, they typically target specific modification types and require additional treatments. More fundamentally, most readouts are still anchored at the cDNA level, making it difficult to simultaneously resolve full‐length transcript structures, modification landscapes, and poly(A) tail features at the single RNA molecule resolution.

A fundamental limitation shared by both short‐read RNA‐seq and long‐read cDNA sequencing is that they sequence cDNA rather than native RNA molecules. This introduces three major sources of bias. First, they differ at the template level. In cDNA‐based sequencing, reverse‐transcribed products serve as the sequencing templates. Consequently, any biases introduced during RT, PCR amplification, or library preparation, such as template switching, 3′‐end bias, GC‐content‐related bias, and amplification noise, are irreversibly embedded in the final sequencing data [25]. Second, the information content is inherently restricted. Conventional RNA‐seq can only infer RNA modifications or poly(A) tail properties indirectly, for instance, by leveraging chemical or enzymatic enrichment or depth‐of‐coverage patterns to deduce a limited set of marks such as m^6^A or m^5^C [26, 27]. It cannot directly and simultaneously read “sequence, modification, and poly(A) tail” on the same individual molecule. Third, the observable molecular information remains incomplete. Transcripts truly exist as RNA molecules, whereas cDNA represents only a copied derivative. Under conditions such as disease, stress, or aging, RT efficiency can be differentially affected by complex secondary structures [28, 29] or modification‐dense regions [30, 31, 32], leading to systematic under‑representation of biologically relevant transcripts [33]. DRS based on nanopore sequencing platforms overcomes these limitations by enabling single‐molecule, long‐read sequencing directly on native RNA templates. In this setup, RNA molecules are threaded through nanopores under the control of a motor protein. Their nucleotide sequence and modification status jointly shape the ionic current trace, which is then decoded by basecalling algorithms into sequence and can be further interrogated to infer modification events [9, 34]. More recently, the SQK‐RNA004 chemistry, fully commercialized in 2024, delivers improved performance through redesigned nanopores and optimized motor proteins, and when paired with Dorado software starting from v1.0.0, it enables the identification of up to eight RNA modifications using nanopore DRS data. This technology thus provides new opportunities to investigate RNA molecules in their native biochemical and structural context.

Against this backdrop, one‐shot measurement of “full‐length transcript, poly(A) tail features, and RNA modifications” on the same molecule is rapidly emerging as a central goal for the next phase of RNA biology. Traditional multiplatform approaches struggle to meet the combined demands of throughput, cost, and quantitative consistency. DRS offers a conceptually appealing “one‐shot, multi‐layer readout” solution, creating new space for what can be regarded as a truly integrated “RNA‐centric biology” that unifies the transcriptome, epitranscriptome, and poly(A) tail dynamics. Rather than viewing nanopore DRS as a replacement for existing transcriptomic methods, we position it as a complementary, molecule‐resolved platform that provides an integrative entry point into the multiple layers of RNA regulation. In this Review, we therefore focus not only on what DRS can currently measure, but also on what still depends on model‐based inference, which applications are best suited to DRS, and what forms of benchmarking and orthogonal validation are required to support different classes of biological conclusions. Building on the substantial body of methodological and applied studies on DRS published in recent years, we here provide a systematic overview of this technology, covering its physical principles, experimental features, cutting‐edge applications, data‐analysis strategies, and method development. Our goal is to connect the full pipeline “from biophysical principles to experimental design, from data processing to algorithmic innovation, and from basic discovery to translational applications,” thereby offering researchers in RNA biology a practically oriented and readily actionable review of DRS.

## NANOPORE DIRECT RNA SEQUENCING: FUNDAMENTAL PRINCIPLES AND DEVELOPMENTAL TRAJECTORY

**Figure 2:**
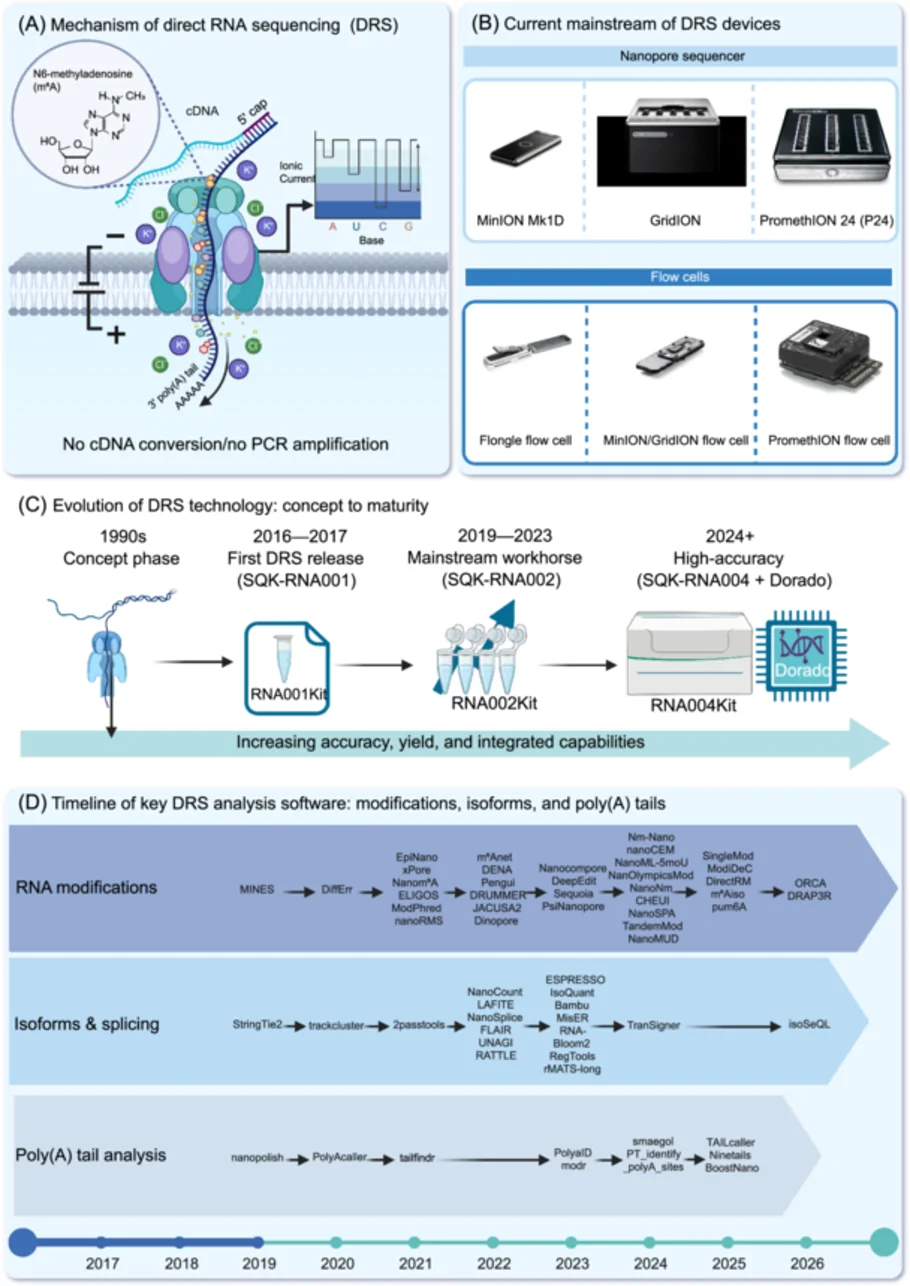
Overview of DRS technology, platforms, and software. (A) Principle of DRS. Native poly(A) + RNA molecules are sequenced directly through a biological nanopore without cDNA synthesis or PCR amplification. As RNA passes through the pore under an applied voltage, nucleotide‐specific disruptions in ionic current enable basecalling and direct detection of RNA modifications such as m^6^A. (B) Current mainstream nanopore platforms and consumables supporting DRS, including MinION Mk1D, GridION, and PromethION instruments, together with corresponding flow cells. (C) Evolution of DRS technology from early conceptual foundations to mature, relatively high‐accuracy workflows. Key milestones include the first commercial DRS kit (SQK‐RNA001; 2016−2017), establishment of SQK‐RNA002 as a mainstream workhorse (2019−2023), and recent advances integrating SQK‐RNA004 chemistry with Dorado basecalling (2024+), resulting in increased accuracy, throughput, and functionality. (D) Timeline of major computational tools developed for DRS data analysis, highlighting methods for RNA modification detection, isoform and splicing analysis, and poly(A) tail length estimation. The progression illustrates rapid expansion and increasing sophistication of the DRS bioinformatics ecosystem from 2017 to the present. m^6^A, N6‐methyladenosine. Created in BioRender. Li, Y. (2026) https://BioRender.com/d8phpl6.

DRS employs nanopore sequencing technology to perform single‐molecule sequencing of native RNA molecules [34]. Unlike traditional approaches, this method does not require RT of RNA into cDNA, thereby avoiding biases that may be introduced during RT and PCR amplification [35]. The underlying principle is based on the translocation of single‐stranded RNA through a biological nanopore embedded in a membrane under an applied electric field. Because RNA molecules carry a negative charge, a motor protein drives the RNA into the nanopore at a controlled speed under voltage. Typically, the motor protein attaches to the poly(A) tail at the 3′ end of mRNA via an adapter sequence, enabling the RNA to translocate through the pore in a 3′‐to‐5′ direction. When a short segment of the RNA strand (approximately 5 nt) occupies the narrow constriction of the nanopore, it impedes ionic current flow and produces characteristic changes in the electrical signal (Figure 2A) [36, 37]. Different nucleotide sequence combinations generate current changes of varying magnitudes, allowing the electrical signals to be regarded as “fingerprints” of the corresponding k‐mer sequences [9, 35, 38, 39]. The sequencing device records these current signals in real time and interprets them into nucleotide sequences using deep learning algorithms, a process known as basecalling [38, 40]. Recent high‐accuracy models developed by ONT leverage neural networks to interpret current waveforms, enabling real‐time and increasingly precise RNA sequencing [41, 42].

Although DRS has only recently matured into a robust platform, its conceptual foundation dates back several decades. In 1989, the American scientist David Deamer first proposed the idea of driving single‐stranded DNA or RNA molecules through nanoscale pores to read their sequence information [43]. In the mid‐1990s, key experimental advances were achieved. Kasianowicz and colleagues observed ionic current blockade events when single‐stranded nucleic acids passed through an α‐hemolysin biological pore which laid the foundation for subsequent single‐molecule sequencing [43]. By the late 1990s, “nanopore sequencing” gradually emerged and was adopted as a formal term and technological approach. During the same period, Hagan Bayley and colleagues conducted related technical research. They developed a method to detect molecular characteristics by measuring stochastic fluctuations in nanopore ionic currents, known as stochastic sensing [44, 45]. They also recognized that this approach could be applied to DNA sequencing. The *Staphylococcus aureus* membrane channel protein α‐hemolysin has an inner diameter of approximately 1.4−2.4 nm [43, 46]. It was the first nanopore proven to allow the passage of RNA and DNA homopolymers while producing identifiable ionic current blockade signals [43, 47, 48].

The founding of ONT by Hagan Bayley and Gordon Sanghera in 2005 greatly accelerated the transition of nanopore sequencing from concept to application [43]. In the decade following ONT's establishment, the company's research team continuously worked on optimizing biological nanopore materials and sequencing enzymes [39]. For example, they engineered the protein CsgG to develop the R9 series of nanopores and upgraded sequencing enzymes, gradually enabling high‐throughput DNA sequencing [49, 50].

This innovation broke the dependence of traditional sequencing on laboratory settings and enabled long‐read DNA sequencing to be performed outside conventional laboratories [43]. In December of 2016, ONT provided the first DRS reagent kits (code: SQK‐RNA001) to a small group of researchers for testing (https://nanoporetech.com/news/news-direct-rna-sequencing-minion-new-paper-preprint). In April 2017, ONT further expanded access by officially making RNA direct sequencing kits available to its user community (https://nanoporetech.com/news/news-direct-rna-sequencing-nanopore-opens-more-users), and preliminary experimental results of direct RNA sequencing were presented at the London Calling conference in May of the same year (https://nanoporetech.com/news/news-london-calling-2017-day-2-updates). In 2018, the ONT research team reported the first comprehensive DRS study in Nature Methods [9], demonstrating full‐length sequencing of yeast mRNA and the feasibility of detecting RNA modifications directly from electrical signals [9].

Since the introduction of the first‐generation SQK‐RNA001 kit, DRS has undergone rapid technological improvements (Figure 2B) [51]. Early applications using this kit demonstrated its potential for complex transcriptome analysis, including simultaneous assessment of transcript diversity, poly(A) tail length, and RNA modifications in human samples [35]. In 2020, a plant transcriptome study showed that DRS could reveal splicing isoforms, alternative polyadenylation (APA) site selection, and m^6^A modification patterns in *Arabidopsis thaliana* mRNA [52]. Building upon the SQK‐RNA001 kit, ONT released an improved RNA sequencing kit, SQK‐RNA002, in 2019. This updated version substantially improved both data yield and stability, and became the mainstream DRS solution between 2019 and 2023 [53]. More recently, the SQK‐RNA004 chemistry, introduced in 2023 and fully commercialized in 2024, further enhanced performance through redesigned nanopores and optimized motor proteins (Figure 2C). While maintaining similar error profiles, SQK‐RNA004 substantially reduces overall error rates, achieving up to ~98% accuracy (F1‐score: 96%–99%), compared with ~90%–94% for SQK‐RNA002, while also increasing read output [53, 54, 55].

A key strength of DRS lies in its ability to generate multidimensional data beyond sequence information alone. By directly sequencing native RNA molecules, DRS preserves chemical modifications such as m^6^A, Ψ, m^5^C, and inosine, which subtly alter ionic current signals and can be detected computationally (Figure 2D) [34, 56, 57, 58]. The latest DRS basecalling algorithms incorporate dedicated electrical signal models that are trained on modification‐aware data and can be used to infer multiple RNA modification types and their positions from nanopore current signals [59]. Because the 3′ poly(A) tail of mRNA consists of repeated identical nucleotides, its corresponding segment produces a low‐variance signal in the sequencing current. By analyzing this signal and correcting for the translocation speed of the molecule through the nanopore, the poly(A) tail length of each transcript can be estimated [35]. Dedicated software tools, such as nanopolish‐polya, have been developed for this purpose. These tools segment the raw electrical signal into poly(A) regions and measure their dwell time. The number of adenosines in the tail can then be inferred from these measurements [35, 52]. In summary, nanopore DRS enables long‐read, amplification‐free RNA sequencing through the interpretation of electrical current signals. This technology not only provides sequence information but also simultaneously captures multiple layers of transcriptomic features, including splicing variation, transcript structure, base modifications, and poly(A) tail length [52, 60].

ONT also released a new generation of open‐source basecalling software, Dorado (https://nanoporetech.com/platform/accuracy), which employs improved deep‐learning basecalling models that enhance accuracy and speed. Since its release in 2023, Dorado has undergone rapid development, transitioning from version 0.x to 1.x. The 1.1 and 1.2 updates in 2024 introduced optimized High Accuracy (HAC) and Super Accurate (SUP) models, while the 1.3 series in 2025 added support for 2′‐O‐methylation (Nm). The latest model (1.4.0) optimized for SQK‐RNA004 signal profiles, achieved substantial improvements in read accuracy. For example, SQK‐RNA004 data base‐called with Dorado (v0.8.0) achieved a median alignment identity of 98.67% (reads ≥ 200 aligned bases) whereas SQK‐RNA002 reads base‐called with Guppy (v6.3.8) reached 90.65%, highlighting the higher single‐read accuracy obtained when using SQK‐RNA004 together with Dorado [53]. Deep learning models of Dorado also support *de novo* detection of multiple RNA modifications, such as m^6^A, m^5^C, inosine, Ψ, and Nm, within a single DRS run. In validation experiments with SQK‐RNA004 data, Dorado's Ψ and m^6^A models achieved high performance, with accuracies and F1‐scores generally in the mid‐90% range or higher [53, 61]. Collectively, these advances mark the transition of nanopore DRS into a relatively high‐accuracy era, enabling more reliable characterization of transcript sequences and epitranscriptomic features [62]. From proof‐of‐concept to the implementation of SQK‐RNA001 and SQK‐RNA002, and now to the maturation of SQK‐RNA004 combined with Dorado, each technological iteration represents a step toward higher accuracy and broader application of DRS [53]. These advances provide valuable tools for comprehensive analysis of transcriptional regulation. With continued optimization of algorithms and sequencing chemistries, DRS is expected to play an increasingly important role in transcriptome research, RNA modification mapping, and RNA‐based therapeutic development [53, 63].

## COMPARATIVE ANALYSIS OF DRS AND CONVENTIONAL RNA SEQUENCING TECHNOLOGIES

### Comparison of DRS and short‐read sequencing techniques

**Figure 3:**
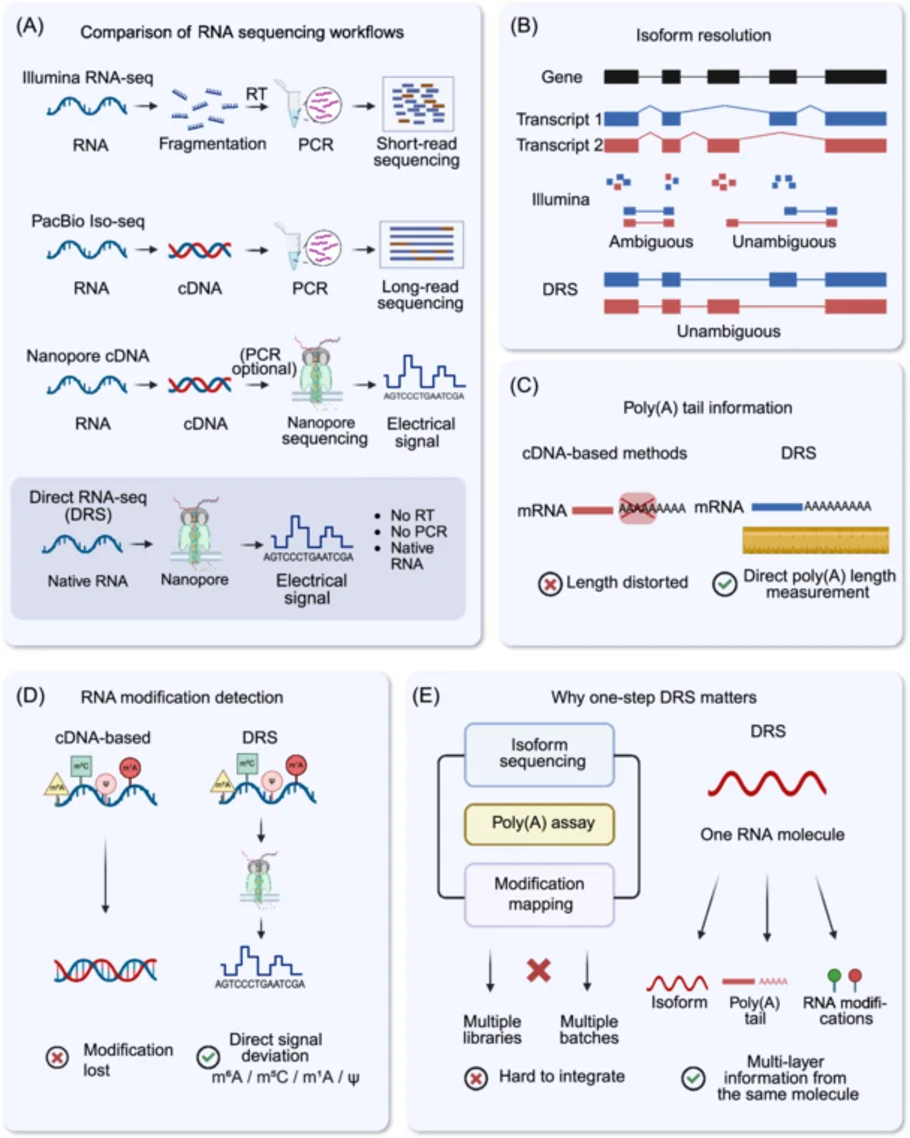
Comparison of RNA sequencing strategies and features captured by DRS. (A) Comparison of workflows among principal RNA sequencing modalities. Illumina RNA‐seq necessitates RNA fragmentation, subsequent RT and PCR amplification to produce short reads. PacBio Iso‐Seq and nanopore cDNA sequencing provide extended reads from cDNA templates, generally incorporating reverse transcription and frequently PCR amplification. Conversely, nanopore DRS sequences native RNA molecules straight via the pore, generating an electrical current signal for basecalling without reverse transcription or PCR, thus minimizing conversion and amplification‐related biases. (B) Isoform resolution. Short‐read RNA sequencing sometimes produces reads that correspond to several transcript isoforms, leading to unclear assignments. In contrast, long‐read methodologies, especially DRS, provide clearer reconstruction and quantification of full‐length isoforms and splice junction connectivity. (C) Information regarding the Poly(A) tail. cDNA‐based library building may skew the perceived length of poly(A) tails due to RT/PCR errors, whereas DRS facilitates direct measurement of poly(A) tail length using native RNA signals and/or read characteristics. (D) Detection of RNA modifications. In cDNA synthesis, numerous RNA modifications are not maintained in the resultant cDNA sequence, but DRS retains native chemical modifications that can be deduced from modification‐related variations in nanopore current (e.g., m^6^A, Ψ, m^1^A and m^5^C). (E) Justification for a single‐step DRS. Through the interrogation of a singular RNA molecule, DRS can concurrently ascertain isoform identity, poly(A) tail characteristics, and modification signatures, hence enhancing integrative, multilayer transcriptome and epitranscriptome investigations compared to methods necessitating distinct assays and batches. cDNA, complementary DNA; m^5^C, 5‐methylcytosine; PCR, polymerase chain reaction; RT, reverse transcription; Ψ, pseudouridine. Created in BioRender. Li, Y. (2026) https://BioRender.com/i6hbowb.

The rapid evolution of transcriptomics has substantially enhanced our ability to investigate RNA function, processing, and regulation. Short‐read RNA‐seq (srRNA‐seq), primarily based on NGS platforms such as Illumina and Ion Torrent, remains the most widely used approach because it offers high throughput, strong reproducibility, and a mature analytical and statistical ecosystem [64]. However, as transcriptome research increasingly emphasizes isoform‐resolved questions, including which transcript variants are produced, how splicing patterns are coordinated, and which isoforms dominate, the limitations inherent to short read length become more apparent [39, 65, 66, 67]. DRS provides a complementary and, in several respects, advantageous strategy by sequencing native RNA molecules without RT or PCR amplification and by generating long reads that often span full‐length transcripts (Figure 3A) [9, 26, 35, 68, 69].

Accurate quantification of genes and transcript isoforms is central to transcriptome analysis. With short reads of typically 150−200 bp [70], gene‐level expression can be estimated robustly at scale using splice‐aware alignment (e.g., STAR [71], HISAT2 [72]), feature‐level counting (e.g., featureCounts [73], HTSeq [74]), and well‐validated differential expression frameworks (e.g., DESeq2 [75], edgeR [76], limma/voom [77]). At the isoform level, quantification is usually performed through pseudo‐alignment or expectation‐maximization(EM)‐based models (e.g., Salmon [78], kallisto [79], RSEM [80]), which infer transcript abundances by probabilistically allocating fragments across candidate isoforms. This approach is computationally efficient and can be accurate when isoforms are sufficiently distinct.

A key limitation of srRNA‐seq stems from read fragmentation. When transcripts share exons or differ by only a few splice junctions, many reads cannot be uniquely assigned to a single isoform (Figure 3B). Isoform quantification therefore depends heavily on statistical deconvolution and becomes sensitive to reference annotation completeness, sequence mappability, and technical artifacts introduced during library preparation, including GC‐content‐related effects and PCR‐related distortions. These dependencies can reduce both accuracy and interpretability of isoform‐level estimates, particularly in complex genomic loci and among highly homologous transcript families [81, 82, 83, 84].

DRS mitigates these challenges by generating long reads from native RNA molecules, preserving transcript structure while avoiding amplification‐associated biases. Because individual reads frequently span multiple exon‐exon junctions and often cover entire transcripts, DRS provides direct structural evidence for transcript identity and substantially reduces ambiguity in read assignment (Figure 3B) [85]. Isoform‐aware quantification can be performed using dedicated tools such as NanoCount [86] or through long‐read workflows that combine long‐read alignment (e.g., minimap2 [87]) with isoform reconstruction, refinement, and filtering (e.g., FLAIR [88], StringTie2 [89], IsoQuant [90], Bambu [91], miniQuant [92]). Downstream differential analysis can then be conducted using established count‐based frameworks such as DESeq2 or edgeR. In this way, DRS reduces reliance on inference‐driven isoform quantification models.

The advantages of long reads are especially evident in AS analysis. srRNA‐seq is highly effective for identifying splice junctions and quantifying local event‐level changes, typically by combining splice‐aware mapping with event‐centric tools such as rMATS‐long (https://github.com/Xinglab/rMATS-long) [93], SUPPA2 [94], MAJIQ [95], and DEXSeq [96]. While this strategy offers strong sensitivity for changes at specific junctions or splicing events, it provides limited information about long‐range exon connectivity. As a result, reconstructing full‐length isoforms and determining how multiple splicing events co‐occur within the same transcript remains challenging. This limitation is particularly evident in cases involving complex splicing patterns, such as exon skipping, alternative donor or acceptor usage, mutually exclusive exons, and multi‐event coupling, and it can reduce the completeness of novel isoform discovery [14].

DRS addresses this gap by directly capturing exon connectivity. Long reads preserve the ordered chain of exon‐exon junctions within individual transcripts, which strengthens isoform‐resolved splicing analysis and supports more accurate identification of complete splice isoforms. Long‐read data also facilitate interpretation of transcriptome features that can be difficult to resolve from short fragments alone, including fusion transcripts and additional RNA processing outcomes such as editing or circularization when supported by appropriate analysis [97, 98]. Accordingly, DRS pipelines that integrate long‐read alignment with isoform reconstruction and refinement can reveal a more complex splicing landscape than short‐read approaches [52].

A closely related application is the identification of the dominant transcript isoforms for each gene, which is critical for interpreting isoform‐specific regulation and functional consequences [99, 100, 101]. In srRNA‐seq, dominant isoforms are generally inferred from estimated isoform abundances. This inference can be uncertain when isoforms are highly similar because fragment assignment is ambiguous and library preparation biases can distort relative abundance estimates, especially in genes with dense isoform catalogs [64, 82]. By enabling direct counting of reads corresponding to complete isoform structures, DRS can reduce ambiguity in isoform assignment. However, DRS introduces platform‐specific biases that must be considered. Poly(A)‐based capture commonly enriches the 3′ end, and RNA degradation or sequencing‐related truncation can reduce representation of intact 5′ ends [38]. These effects can complicate estimation of full‐length isoform abundance and make results sensitive to isoform definitions, filtering criteria, and correction strategies implemented in tools such as FLAIR, IsoQuant, and Bambu.

In summary, srRNA‐seq remains highly effective for scalable and cost‐efficient profiling, with a robust statistical foundation for gene‐level differential expression and event‐centric splicing analyses. Its limited read length, however, restricts unambiguous isoform assignment and full‐length transcript reconstruction, increasing reliance on probabilistic inference and reference annotations. DRS offers several advantages for transcript identification, AS reconstruction, and isoform‐resolved quantification by capturing long, often full‐length, native RNA molecules without RT or PCR amplification. Despite remaining challenges, including higher per‐read error rates, end‐coverage biases, and more complex analytical workflows, continued improvements in Nanopore chemistry, basecalling, and long‐read computational methods are expected to further improve the applicability of DRS in isoform‐resolved transcriptomics and integrative meta‐omics research.

### Comparison of DRS with other long‐read RNA sequencing techniques

Nanopore DRS, PacBio Iso‐Seq, PacBio Kinnex transcriptome sequencing, ONT cDNA sequencing, and ONT Tail Iso‐Seq collectively constitute the current core toolkit for long‐read RNA sequencing. However, they differ substantially in target molecules, library construction strategies, information layers preserved, and sources of bias [81, 84, 102, 103]. At present, Chinese domestic nanopore sequencing platforms (e.g., Qitan, MGI, Pervitro) are primarily optimized for double‐stranded DNA sequencing, with dedicated RNA library preparation kits still under development. They are therefore not considered further in this discussion.

PacBio Iso‐Seq, PacBio Kinnex, ONT cDNA sequencing, and ONT Tail Iso‐Seq all convert RNA into cDNA, and then into double‐stranded DNA, via RT and PCR, thereby producing long‐read‐compatible templates. The canonical PacBio Iso‐Seq workflow comprises full‐length cDNA synthesis, size selection, SMRTbell library preparation, and High‐Fidelity (HiFi) sequencing, yielding highly accurate reads, often approaching 99%. These high‐fidelity reads enable precise delineation of exon‐intron boundaries, detailed characterization of complex AS, and construction of high‐confidence reference transcript annotations [104, 105]. PacBio Kinnex transcriptome protocols build upon Iso‐Seq by optimizing library preparation, incorporating barcodes/UMIs, and introducing normalization steps. These modifications improve sequencing throughput and resource utilization, allowing broader coverage of samples or cell types while attempting to mitigate systematic quantitative distortions affecting transcripts at extremely high or low abundance [106, 107, 108]. Nevertheless, Kinnex remains an intrinsically cDNA‐based strategy, and poly(A) tail features and RNA modifications at the single‐molecule level cannot be directly preserved.

Standard ONT cDNA sequencing (e.g., SQK‐PCS109, SQK‐PBK004) similarly employs oligo(dT) and/or random‐primed RT followed by PCR amplification. Its main advantages include platform flexibility, relatively modest hardware requirements, and reduced per‐sample cost, making it well‐suited for cost‐effective delineation of long‐read transcript structure, major splicing events, and a subset of gene fusions. However, per‐base accuracy and indel rates depend strongly on the basecalling model and subsequent error‐correction procedures. ONT Tail Iso‐Seq (SQK‐PCS114 and SQK‐PCB114), developed from this framework, introduces 3′‐end‐oriented library design and poly(A)‐targeted adapters to enrich information on transcript 3′ ends and poly(A) tails. In this framework, reads typically initiate at the 3′ end and traverse the poly(A) tract, allowing estimation of tail length at the cDNA level and thereby providing more detailed “transcript‐structure plus poly(A) feature” profiles. Nevertheless, all cDNA‐based workflows are intrinsically dependent on RT and PCR, and therefore subject to systematic biases such as template switching, amplification bias, and chimeric artifact formation.

RT and PCR‐related biases represent major limitations of cDNA‐based long‐read transcriptomics. Long transcripts or regions with complex RNA secondary structures are particularly prone to incomplete RT and premature termination, often leading to 3′ read enrichment and apparent 5′ truncation, which can be misinterpreted as spurious isoforms. RT template switching and PCR breakage‐religation generate chimeric cDNA molecules or incorrect exon combinations, especially in highly expressed transcripts and repetitive regions, thereby confounding the interpretation of exitrons, pseudo‐exon skipping, fusion transcripts, and related structural events [28, 109, 110, 111]. PCR efficiency is further modulated by GC content, fragment length, and local sequence/structure context, introducing quantitative biases both within and across samples [11]. In addition, most RNA modifications are effectively “written through” as canonical bases during RT; only a minority give rise to RT stops or misincorporations, producing noisy and indirect signals that are insufficient for comprehensive modification mapping [28]. Poly(A) tail information is distorted during library preparation and size selection, with short tails prone to partial truncation and very long tails often underestimated, limiting inference to population‐level distributions rather than precise single‐molecule measurements [112].

In contrast, the standard DRS workflow avoids these physical amplification‐related biases at the mechanistic level. DRS can capture, within the same read, the full‐length splicing structure, the 3′ poly(A) tract (Figure 3C), and the corresponding ionic current signals perturbed by RNA modifications. This enables the characterization of exitrons, complex AS patterns, and fusion transcripts without introducing RT/PCR‐derived artifacts, making DRS useful as an orthogonal validation strategy for cDNA‐based results in applications where structural false positives are of major concern [113]. It is important to note, however, that DRS introduces its own class of “signal‐decoding” errors. Homopolymeric stretches and regions with intricate secondary structure remain challenging to decode accurately, and the influence of RNA modifications on current traces continues to be a central focus of algorithmic development and model training [114].

Rather than viewing these technologies as competing alternatives, it is more appropriate to consider them as a configurable and complementary toolkit tailored to specific experimental objectives. Iso‐Seq or Kinnex is typically preferred when the principal objective is to construct a very accurate reference transcriptome. For extensive cohort isoform quantification and mechanism‐focused investigations, ONT cDNA or Tail Iso‐Seq offers a cost‐effective balance between throughput and structural resolution. Investigations centered on poly(A) tail length distributions and 3′‐end structures may emphasize Tail Iso‐Seq or Iso‐Seq/Kinnex techniques that utilize 3′‐end‐optimized protocols. When the research question centers on the interplay between epitranscriptomic regulation, transcript architecture and dynamics of poly(A) tails, or when the system exhibits heightened sensitivity to RT‐PCR induced biases, DRS represents an appropriate methodology. Collectively, these methods are best viewed as an application‐dependent toolkit for transcriptome analysis rather than strictly competing technologies. To guide method selection for common research objectives, their relative applicability is summarized in Table S1. Furthermore, a comprehensive comparison of their main advantages, limitations, typical application cases, and verification requirements is provided (Table S2).

### Comparison of DRS with other modification detection methods and its positioning in modification detection

Currently, strategies for RNA modification identification have been developed, mainly including LC‐MS/MS, specific antibody‐based immunoprecipitation, chemical conversion methods, and nanopore DRS [27, 115, 116, 117]. Among them, DRS has emerged as a promising approach in the field of modification detection due to its unique technical principles [118, 119, 120]. In the following, we systematically summarize the core principles, advantages, and disadvantages of these strategies, focus on analyzing the major advantages of DRS over these technologies, and clarify the positioning of DRS in the field of modification detection.

LC‐MS/MS is a core analytical platform for RNA modification detection based on tandem mass spectrometry. The method leverages liquid chromatography to separate enzymatically digested oligonucleotide fragments or nucleosides according to properties such as molecular weight and polarity. Eluted analytes are subsequently ionized and analyzed by tandem mass spectrometry, where modifications are identified and quantified via characteristic mass‐to‐charge (m/z) peaks, with chromatographic retention assisting in site localization [115, 121, 122, 123]. LC‐MS/MS is distinguished by its high specificity, accurate quantitation, capacity for multiplexed modification analysis, absence of amplification bias, and sequence‐agnostic detection, making it a gold‐standard technique in the field [115, 122, 124, 125, 126]. However, it remains constrained by low throughput, lack of single‐nucleotide resolution, and loss of spatial context of modifications within RNA molecules [123, 124, 127].

Antibody‐based immunoprecipitation combined with NGS is currently the most widely used approach in RNA modification detection. The core step of this method involves the enrichment of modified RNA fragments using antibodies followed by sequencing. This strategy has been widely used for various modifications, such as m^6^A [17, 18, 20, 128, 129, 130], m^5^C [131, 132], 5‐hydroxymethylcytosine (hm^5^C) [133], m^6^Am [20, 134, 135], ac^4^C [136], and m^1^A [137, 138]. For instance, MeRIP‐seq (or m^6^A‐seq) was first developed in 2012 for the detection of m^6^A modification within 100–200 nt fragments using m^6^A‐specific antibodies [17, 18]. With advantages including simple operation, high throughput, low RNA input, and suitability for large‐scale screening experiments, this technology has become a widely used technique for RNA m^6^A modification detection and has promoted the rapid development of epitranscriptomics [139, 140, 141]. Additionally, miCLIP, a single‐base resolution m^6^A detection technology, was developed in 2015 based on the MeRIP‐seq [20]. Its core improvement is the addition of an ultraviolet cross‐linking step, which addresses the limitation of low resolution in MeRIP‐seq. Although these methods have enabled transcriptome‐wide identification of RNA m^6^A modifications at the transcriptome level, however, they still face challenges such as complex experimental procedures, limited quantitative accuracy, antibody preference, and restricted applicability [20, 23, 142, 143].

The pursuit of higher accuracy in RNA modification detection and quantification has driven the development of chemical conversion strategies [131, 144, 145, 146, 147, 148, 149, 150, 151, 152, 153, 154]. Prominent among these are GLORI‐seq, designed for m^6^A mapping, and bisulfite sequencing, adapted for m^5^C profiling [21, 155]. GLORI‐seq enables precise m^6^A detection by treating RNA fragments with glyoxal and nitrite, which selectively convert unmodified adenosine (A) to inosine (I), while m^6^A remains intact. During RT, inosine (I) is read as guanosine (G), allowing m^6^A sites to be identified as retained A signals [21, 155]. Bisulfite sequencing relies on sodium bisulfite to deaminate unmodified cytosine (C) to uracil (U), whereas m^5^C and hm^5^C are resistant. Following PCR, U is converted to thymine (T), enabling methylation site identification via C/T mismatch analysis against a reference genome [145, 146, 147]. The principal strengths of this type of strategy include high specificity and sensitivity, single‐base resolution, and absolute quantitation capability. Additionally, several approaches have also been developed to map other modifications in a transcriptome‐wide manner by coupling this selective labeling reaction to high‐throughput sequencing, such as Ψ‐Seq, Pseudo‐seq, CeU‐Seq and PSI‐seq for Ψ, ac^4^C RedaC for T‐seq, ac^4^C‐seq for ac^4^C, and so on [75, 156, 157, 158, 159, 160]. Nevertheless, these methods are constrained by challenges such as incomplete conversion, RNA degradation, technical complexity, and high dependence on RNA structural accessibility, which may limit their applicability in certain contexts [144, 145, 146, 147, 148].

Enzyme‐assisted RNA modification mapping refers to a suite of techniques that employ modification‐dependent enzymes, such as nucleases, methyltransferases, or deaminases, to recognize, cleave, or chemically alter RNA at specific modification sites [22, 24, 116, 161, 162, 163, 164, 165]. By coupling these enzymatic reactions with high‐throughput sequencing, characteristic signals including truncation patterns or base conversions are generated, enabling precise localization and quantification of modifications at single‐nucleotide resolution. This approach is widely applied to detect RNA modifications, including m^6^A, m^5^C, m^1^A, and Nm [22, 23, 24, 132, 138, 161, 166, 167, 168, 169, 170, 171]. A representative example is MAZTER‐seq (also termed m^6^A‐REF‐seq), which exploits the MazF endonuclease whose cleavage at ACA motifs is selectively blocked by m^6^A, thereby allowing site‐specific detection [24, 161]. Although enzyme‐assisted mapping offers high specificity, intrinsic targeting capability, antibody‐free operation, and base‐level precision, it is constrained by limitations such as pronounced dependence on enzyme activity, a relatively narrow substrate range, workflow complexity, stringent reaction conditions, and reduced sensitivity for low‐abundance modifications [22, 24, 116, 164].

Nanopore DRS directly sequences native RNA molecules while preserving their intrinsic electrical profiles shaped by chemical modifications [34, 118, 119]. By capturing base‐specific disruptions in ionic current and analyzing signal traces using machine learning or deep learning algorithms [56, 114], this approach simultaneously resolves nucleotide sequences and modification states at single‐molecule and single‐base resolution (Figure 3D). Numerous algorithms have been developed to detect and quantify RNA modifications in DRS data sets [52, 172, 173, 174, 175, 176, 177, 178, 179, 180, 181, 182, 183, 184, 185, 186, 187, 188]. When compared with conventional modification sequencing techniques, DRS demonstrates several inherent advantages. It eliminates preprocessing requirements and the associated signal biases, a feature particularly valuable for detecting low‐abundance modifications that are often lost in multistep protocols [114, 119, 186]. Meanwhile, the distinct current signatures produced by different RNA modifications enable concurrent identification and quantification of multiple modification types without the need for separate assays. Furthermore, its amplification‐free design preserves the native modification context at the single‐molecule level, achieving transcriptome‐wide mapping with near base‐level resolution, which is a capability largely unattainable by indirect sequencing methods [34, 116]. Perhaps most notably, DRS uniquely integrates high‐throughput capacity with long‐read capability. This long‐read characteristic reveals continuous modification landscapes along entire RNA molecules and facilitates investigation of correlations between modifications and transcript features such as polyadenylation sites (PAS), splice variants, and secondary structures (Figure 3E). [118, 189].

While DRS has established clear advantages and a distinct niche in RNA modification mapping, it remains under active development and faces several practical challenges. First, the analytical accuracy of modification calling requires improvement, particularly in distinguishing low‐abundance or structurally similar modifications. For instance, addressing misclassification arising from subtle current differences between m^6^A and m^6^Am [120, 177]. Second, bioinformatic tools require further refinement for robust multimodification co‑analysis and de novo modification detection, alongside greater automation to minimize reliance on specialized expertise [162, 190]. Third, per‑sample sequencing costs remain higher than those of established methods like MeRIP‑seq or bisulfite sequencing, limiting large‑scale adoption in resource‑constrained settings [39, 120, 191].

Consequently, DRS is redefining the frontiers of RNA modification research through its distinct analytical advantages, standing as a foundational platform for epitranscriptomic analysis. By preserving and directly reading native RNA molecules, it eliminates inferential steps and inherent biases, offering a direct window into the epitranscriptome. This positions DRS with considerable potential for discovery‐driven profiling of novel modifications, deciphering modification crosstalk on the same molecule, and mapping modifications across complete, unamplified transcripts. As such, DRS is contributing to a shift toward a more direct, comprehensive, and functionally insightful understanding of RNA biology.

### Strategic library modifications advance DRS methodology

Although Nanopore DRS offers notable advantages, such as generating full‐length transcript reads and preserving epitranscriptomic information [9, 35], the conventional DRS workflow faces significant limitations. These include a heavy reliance on native poly(A) tails and an inability to accurately define 5′‐end transcript boundaries [52, 120]. To address these challenges, recent developments have introduced a range of derivative strategies, including end‐labeling, cap‐capture, adapter engineering [192], and in vitro polyadenylation. These innovations have markedly improved the resolution and coverage of DRS, especially for transcription start site (TSS) identification, nascent RNA analysis, nonpoly(A) transcript characterization, and small RNA sequencing [35, 36, 52, 192]. Importantly, all of these derivative protocols are still classified as native DRS within the framework defined above, as the molecule translocating through the nanopore and generating the electrical signal is the native RNA strand. Reverse transcription and hybrid adapter designs function only as auxiliary steps and do not convert the assay into a cDNA‐based long‐read sequencing workflow.

Broadly, these emerging methodologies can be classified into three categories. (1) 3′‐end labeling strategies focus on the transcript 3′‐terminus and enable the incorporation of RNAs lacking native poly(A) tails into the DRS workflow through in vitro polyadenylation or ligation of poly(A)‐containing adapters [120, 191, 193]. These approaches have been widely adopted for single‐molecule, long‐read profiling of non‐poly(A) RNA species, including prokaryotic transcripts, viral genomic RNAs, histone mRNAs, and diverse classes of noncoding RNAs (ncRNAs). For example, NERD‐seq enables the detection of multiple non‐coding RNAs without poly(A) tail by in vitro polyadenylation [194]. Moreover, the incorporation of random priming and thermostable reverse transcriptase can resolve highly structured regions commonly observed in SINE RNAs, snoRNAs, and other ncRNAs. (2) 5′ Cap‐capture strategies address the inherent limitations of standard DRS in terms of incomplete 5′‐end coverage and imprecise TSS delineation by employing selective enrichment [52, 195]. In prokaryotes, primary transcripts are typically enriched via chemical tagging of the 5′‐triphosphate group with a desthiobiotin tag, whereas the canonical 5′‐m^7^G‐cap is directly targeted for capture in eukaryotes [196]. When combined with 3′‐end tailing strategies, these methods can detect the full‐length RNA molecules to accurately identify TSS profiles across the entire transcriptome. (3) Advanced derivative strategies extend beyond basic end‐labeling and cap‐capture paradigms to enable the interrogation of dynamic transcriptional processes. Representative examples include nanopore analysis of co‐transcriptional processing (Nano‐COP) and FLEP‐seq [197, 198]. Nano‐COP couples 4‐thiouridine (4sU) metabolic labeling with biotin‐based enrichment to facilitate DRS analysis of nascent RNA populations. In parallel, FLEP‐seq leverages a selective capture mechanism targeting the 3′‐hydroxyl (3′‐OH) group to enrich the elongating RNA molecules. This strategy enables the sequencing of full‐length transcripts engaged with RNA polymerase, thereby providing direct insights into the diversity of isoforms and co‐transcriptional splicing intermediates.

Collectively, these methods harness the distinctive advantages of DRS, including preservation of chemical modifications, generation of long‐read sequences, and compatibility with programmable end structures. Together, these features enable integrated characterization of RNA biology, encompassing processes from transcription initiation and splicing to termination and degradation. As a result, they provide a robust technical framework for the generation of high‐resolution transcriptomic and epitranscriptomic maps.

## THE BIOLOGICAL LANDSCAPE FROM A DRS PERSPECTIVE

**Figure 4:**
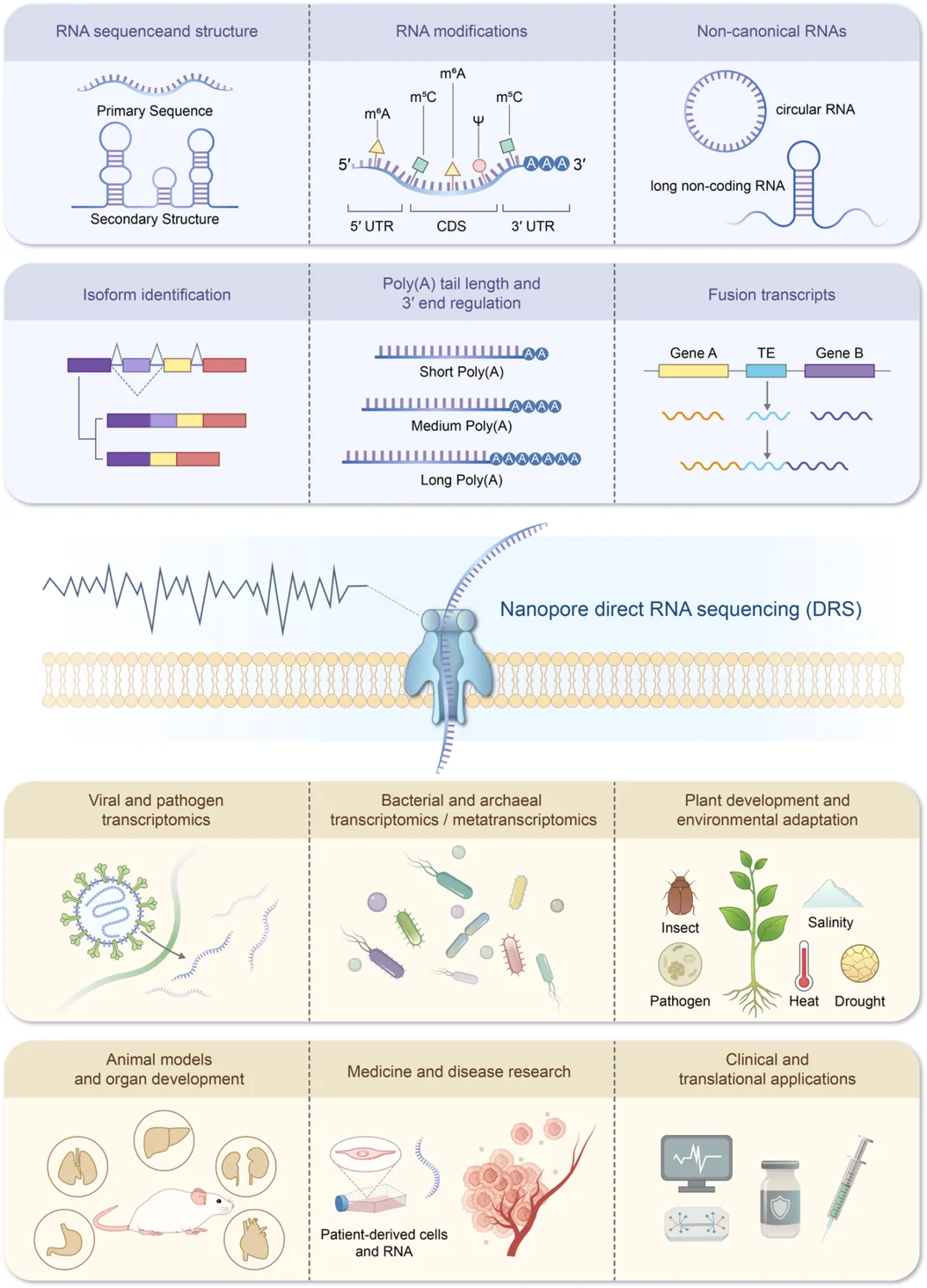
Advantages and biological applications of DRS. Schematic summary of the main strengths and use cases of nanopore DRS. TE: transposable element.

Transcriptome diversity is a fundamental feature of biological systems and a major source of phenotypic complexity in animal and plant species. Beyond gene‐level expression, transcriptome diversity arises from alternative transcription initiation, AS, APA, allele‐specific expression (ASE), RNA editing, chemical modification, and structural heterogeneity. Conventional transcriptomic approaches, dominated by short‐read sequencing and cDNA‐based protocols, fragment RNA molecules and decouple these regulatory layers, limiting their ability to reconstruct native transcript structures. Nanopore DRS overcomes these limitations by directly sequencing full‐length native RNA molecules, thereby providing a single‐molecule view of transcriptome diversity (Figure 4) [9].

### Transcriptomic architecture from the DRS perspective

From the perspective of nanopore DRS, the transcriptome is not simply a collection of expressed genes but a structured population of individual RNA molecules, each defined by a specific combination of TSS, exon‐intron architecture, 3′‐end formation, poly(A) tail features, and chemical modifications [120, 199, 200, 201]. This molecule‐centric view contrasts sharply with conventional srRNA‐seq, which reconstructs transcript models indirectly through computational assembly and often obscures exon connectivity, transcript boundaries, and coordinated regulatory features. By directly observing intact transcripts, DRS reduces ambiguity inherent to short‐read‐based transcript reconstruction and enables systematic discovery of transcriptome features that are difficult to resolve from fragmented data [9, 202]. These include novel isoforms generated by alternative transcription initiation, AS, APA, and gene fusion events, which have been reported across plants, animals, and viral systems [33, 200, 201, 203, 204, 205, 206].

Application of DRS has revealed extensive isoform heterogeneity across species and biological contexts. In organisms such as *A. thaliana*, *Caenorhabditis elegans*, and human cancer models, DRS studies have identified tens of thousands of previously unannotated full‐length isoforms per species, tissue, or developmental condition [203, 204, 207]. This diversity reflects regulated variation in transcription initiation, exon usage, and transcript termination rather than solely stochastic transcriptional noise. DRS further uncovers fine‐grained diversity at transcript 3′ ends, including widespread use of alternative and intronic polyadenylation sites. Such events can generate truncated transcripts encoding proteins with altered or novel functions, as exemplified by intronic polyadenylation of *TLE1* in estrogen‐responsive breast cancer, which produces functionally distinct protein variants [199, 203]. In parallel, DRS enables mapping of epitranscriptomic features, such as m^6^A and m^5^C modifications, within specific transcript contexts in animals, bacteria, and plants, where these modifications often correlate with tissue‐ or condition‐specific expression patterns and characteristic poly(A) tail profiles [200, 208, 209, 210]. Across tissues, developmental stages, and environmental or pathological conditions, combined evidence from DRS and deep RNA sequencing demonstrates that most genes express multiple isoforms with regulated differences in TSS usage, untranslated regions, and terminal exons [65, 201, 202, 207, 211, 212]. Many of these isoforms alter protein‐coding potential or introduce distinct posttranscriptional regulatory elements, thereby influencing protein truncation, localization, stability, and translational efficiency [203, 207, 213, 214].

Collectively, DRS reveals transcriptomes as ensembles of structurally and chemically distinct RNA molecules, whose isoform‐ and modification‐level diversity encodes essential functional and context‐specific information. This architectural view of the transcriptome provides a foundation for mechanistic studies of RNA regulation and highlights the limitations of gene‐centric representations in capturing the full complexity of RNA‐mediated control in biology and disease.

### Alternative splicing and quantitative isoform regulation

AS is a dominant contributor to transcriptome diversity and a central mechanism in development and disease. More than 90% of multi‐exon human genes and approximately 80% of plant genes undergo AS, including exon skipping, intron retention, alternative 5′ or 3′ splice‐site usage, and mutually exclusive exons, which collectively reshape protein‐coding potential and posttranscriptional regulatory elements [82, 215, 216, 217]. Accurately resolving such complexity is challenging for srRNA‐seq, which infers isoforms indirectly from fragmented reads and often collapses distinct transcript variants at the exon or gene level. DRS enables direct, assembly‐free identification of full‐length splice isoforms, providing improved resolution of complex and coordinated splicing patterns that are difficult to reconstruct from short reads [82, 207, 218, 219]. This capability is especially useful for detecting novel or low‐abundance isoforms that would otherwise be missed or misassembled [207, 220].

Full‐length isoform definition also enables improved quantification of isoform usage [82, 218, 219]. Long‐read‐based tools such as LIQA and NanoCount leverage the properties of long reads to estimate isoform abundance and detect differential splicing or isoform switching with greater specificity than short‐read‐based approaches [86, 215, 221]. For example, during human neuronal differentiation, DRS‐based analyses identified thousands of previously unannotated isoforms and revealed hundreds of differentially expressed isoforms and isoform switches, highlighting substantial transcript‐level regulation beyond gene‐level expression changes [86].

Because long‐read sequencing captures complete transcript structures, including untranslated regions (UTRs), it provides a foundation for integrating splicing with downstream functional regulation. Isoform‐resolved transcript models enable investigation of isoform‐specific translational regulation, such as how alternative UTRs, retained introns, or nonsense‐mediated decay‐prone isoforms modulate translational efficiency without altering total RNA abundance [82, 221, 222]. Although most current translational efficiency metrics are computed at the gene level, the structural information provided by DRS and related long‐read approaches makes transcript‐level modeling of differential translational efficiency increasingly feasible when integrated with ribosome profiling or proteomics data.

An additional strength of long‐read sequencing is its ability to support ASE and haplotype‐resolved isoform analysis [223, 224, 225]. Long reads spanning multiple heterozygous variants allow direct phasing of transcript isoforms to parental haplotypes, enabling detection of allele‐specific splicing and expression. Methods such as IsoPhase, HapIso, and IDP‐ASE use long‐read transcriptomes to reconstruct haplotype‐specific isoforms, identify imprinted genes and parent‐of‐origin effects, and dissect *cis*‐ and *trans*‐regulatory influences in systems ranging from maize to human cells [223, 224, 225].

Together, nanopore DRS and related long‐read approaches enable integrated analysis of AS, quantitative isoform regulation, translational control, and ASE at transcript resolution. This isoform‐centric view reveals regulatory mechanisms that are largely invisible to gene‐centric, short‐read analyses and provides critical insights into RNA‐mediated disease processes.

### Poly(A) tail dynamics and 3*′*‐end regulation

APA is a pervasive mechanism shaping transcriptome diversity and a major determinant of RNA stability, subcellular localization, and translational output [35]. By selecting distinct cleavage and PAS, APA generates mRNA isoforms with different 3′ UTR or, in some cases, altered coding sequences, thereby rewiring posttranscriptional regulatory programs [200, 226, 227]. Accurate characterization of APA is therefore essential for understanding RNA fate in both physiological and disease contexts. DRS is uniquely suited for studying APA because sequencing initiates at the native 3′ end of RNA molecules and proceeds through the entire poly(A) tail [60, 210, 228]. This provides direct evidence of transcript termination sites, enabling improved mapping of poly(A) site usage across transcript isoforms, even in complex or heterogeneous samples. In contrast, short‐read APA methods, including DaPars, IsoSCM, APAtrap, and IntMAP, typically infer poly(A) sites from read coverage patterns and often rely on prior annotations, which can obscure complex isoform architectures and coordinated RNA processing events [200].

By reading through native cleavage sites and poly(A) tails, long‐read and DRS‐based approaches directly resolve isoform‐specific 3′ ends and avoid ambiguities associated with transcript reconstruction. When combined with dedicated tail analysis tools such as tailfindR, Nanopolish, Poly(A)tailor, and Ninetails, ONT DRS enables single‐molecule assignment of poly(A) site usage, tail length, and tail composition to specific transcript isoforms [60, 228, 229, 230]. This isoform‐resolved perspective is particularly valuable given that more than half of human genes use multiple poly(A) sites. A large‐scale plant full‐length RNA atlas reported more than 120 million polyadenylated mRNA molecules across multiple tissues and species, uncovering conserved, tissue‐specific poly(A) tail‐length distributions, including reproducible peaks around ~20 nt and ~45 nt in many tissues [231]. Poly(A) tail length itself is dynamic and developmentally regulated, with important consequences for RNA stability and translational control. Genome‐wide studies using approaches such as TAIL‐seq [232], Poly(A)‐seq [233], and DRS‐based tail profiling have shown that highly expressed and efficiently translated mRNAs often carry relatively short steady‐state poly(A) tails in many non‐embryonic systems [226, 234, 235]. Rather than uniformly predicting translational efficiency, tail length is more closely associated with RNA half‐life and pathway‐specific regulatory programs [60, 234, 236]. In addition, widespread incorporation of nonadenosine residues, such as uridylation, guanylation, or mixed tails, adds another layer of regulation by modulating RNA decay and stability [232, 233]. Recent DRS‐based methods, including Ninetails, now enable detection of internal non‐A residues within poly(A) tails in endogenous and therapeutic mRNAs [229].

Importantly, APA and poly(A) tail regulation are closely interconnected with other RNA processing steps. AS and 3′‐end processing exhibit extensive mechanistic crosstalk, and long‐read technologies are essential for resolving coordinated AS‐APA programs on individual transcripts [226, 227, 237]. DRS and PacBio‐based Iso‐seq analyses demonstrate that poly(A) tail length and composition can vary systematically between isoforms derived from the same gene, supporting a model in which splicing decisions, cleavage‐site choice, and tail regulation are co‐regulated to shape RNA stability and fate [210, 230, 232, 238].

These studies support the view that APA and poly(A) tail length and composition form a tightly coordinated 3′‐end regulatory layer. By uniquely linking cleavage site selection, isoform structure, and tail features on the same RNA molecule, nanopore DRS enables an isoform‐resolved understanding of 3′‐end regulation and its role in transcriptome diversity, gene regulation, and disease.

### RNA chemical modifications and epitranscriptomic diversity

RNA chemical modifications add a critical dimension to transcriptome diversity by modulating RNA structure, stability, splicing, localization, and translation [239]. Unlike antibody‐based enrichment or chemical conversion approaches, nanopore DRS detects RNA modifications directly through characteristic perturbations in ionic current signals as native RNA molecules translocate through the nanopore [118, 120, 218, 240]. Consequently, DRS can be used to infer several classes of RNA modifications on single molecules and at single‐nucleotide resolution from nanopore current signals, especially when combined with dedicated modification‐calling models [116, 118, 218, 240, 241]. Recent advances in SQK‐RNA004 pore chemistry and basecalling models such as Dorado now enable implements modification‐aware basecalling models that infer m^6^A, Ψ, m^5^C, Nm, and inosine, improving accuracy and F1 scores in benchmark data sets [53]. Nevertheless, orthogonal validation remains essential, particularly for low‐stoichiometry or context‐dependent modifications.

A major advantage of DRS lies in its preservation of full‐length transcript context. Long reads allow modification calls to be assigned to specific splice and APA isoforms rather than being aggregated at the gene or exon level [60, 118, 218, 240, 242]. This isoform‐resolved view is crucial, as RNA modifications often exhibit variant‐specific distributions that are obscured by short‐read or enrichment‐based methods. Community resources such as DirectRMDB now aggregate hundreds of thousands of DRS‐derived modification sites spanning multiple modification types and explicitly support isoform‐level exploration of epitranscriptomic patterns [240]. Analytical tools such as R2Dtool further integrate isoform‐mapped modification sites with open reading frames, splice junctions, and untranslated regions, enabling systematic analysis of how modification landscapes shift with AS or 3′‐end choice [242]. DRS‐based studies have begun to illuminate coordinated regulation between RNA modifications and other layers of RNA processing. For example, in human leukemia cells, long‐read DRS enabled joint profiling of m^6^A deposition, poly(A) tail length, transcript abundance, and splicing, revealing coordinated transcript‐specific changes following perturbation of the m^6^A writer [60]. Such analyses demonstrate how chemical modifications interact with RNA processing and stability in a highly context‐dependent manner. Widely studied modifications, including m^6^A, m^5^C, Ψ, and A‐to‐I editing, have been implicated in the regulation of splicing, nuclear export, RNA decay, translation, and subcellular localization, and are linked to cancer, cardiovascular disorders, and inherited diseases [81, 116, 243, 244, 245]. In plants, DRS‐based m^6^A profiling has progressed from proof‐of‐concept to quantitatively benchmarked applications. For example, in *Populus trichocarpa* stem‐differentiating xylem, Nanopore‐based m^6^A inference identified 3253 m^6^A‐modified genes, of which 2626 overlapped with MeRIP‐seq results, demonstrating substantial concordance at the gene level while preserving transcript‐specific resolution [172]. Importantly, accumulating evidence indicates that dysregulated RNA modification often acts at the level of specific transcript variants rather than entire genes, leading to isoform‐selective effects on RNA fate [81, 243, 244]. DRS‐based protocols now enable differential modification analysis across conditions, perturbations, and cell states, facilitating dynamic studies of epitranscriptomic regulation in disease models [60, 118, 246].

Together, these advances establish nanopore DRS as an important technology for charting RNA chemical modifications in their native isoform context. By revealing variant‐specific and combinatorial epitranscriptomic programs on individual RNA molecules, DRS provides insights into the functional diversity of the transcriptome and its roles in development, disease, and therapeutic response [177].

### Discovery of noncanonical and noncoding RNA species

Beyond protein‐coding transcripts, DRS has substantially expanded our understanding of transcriptome diversity by enabling systematic discovery and characterization of noncanonical RNA species. Long noncoding RNAs (lncRNAs), circular RNAs (circRNAs), transfer RNAs (tRNAs), and other small or highly structured RNAs are often poorly captured by conventional RNA‐seq protocols owing to their size, secondary structure, low abundance, or lack of polyadenylation. DRS is particularly powerful for profiling lncRNAs, as it captures long, often low‐abundance transcripts with their native 5′ and 3′ boundaries, splice patterns, and poly(A) tail features intact [247]. This overcomes biases associated with poly(A)‐selected or non‐strand‐specific srRNA‐seq, which frequently miss nonpolyadenylated lncRNAs or mis‐assign transcript orientation. In *Arabidopsis*, integration of DRS with low‐abundance‐aware isoform discovery identified more than 1100 previously unannotated lncRNAs and revealed that approximately one‐third of lncRNAs lack poly(A) tails [210]. Moreover, poly(A) tail length and m^6^A modification status were shown to jointly regulate lncRNA abundance and stability, underscoring the importance of isoform‐resolved analysis, particularly in disease contexts where only specific lncRNA variants may be functional [248, 249, 250].

circRNA represents another class of noncanonical transcripts that benefit from long‐read sequencing. Because circRNAs are nonpolyadenylated, their detection typically requires ribosomal RNA (rRNA) depletion or specialized enrichment strategies. Long‐read nanopore approaches have become essential for reconstructing full‐length circRNA sequences and defining their exon composition [251, 252, 253]. For example, the CIRI‐long workflow combines circular RT with nanopore sequencing to achieve substantial enrichment of circRNAs and to resolve complex variants, including mitochondrial and read‐through circRNAs in mouse brain [252]. These studies highlight the importance of long, processive reads for distinguishing lowly expressed circRNAs from linear isoforms and for precisely mapping back‐splice junctions and internal structures, which are critical for functional characterization and biomarker discovery [251, 253, 254, 255]. tRNA poses distinct technical challenges owing to its short length, extensive secondary structure, and dense chemical modification. Customized nanopore DRS protocols and analytical pipelines, including direct tRNA adapters, Nano‐tRNA‐seq strategies, and updated pore chemistries, now enable end‐to‐end sequencing of full‐length tRNAs at single‐molecule resolution [36, 192, 256]. These approaches allow simultaneous quantification of tRNA isoacceptor abundances and detection of numerous modification types on individual molecules, revealing coordinated “modification circuits” within tRNA structural elements such as the T loop, as well as stress‐ or condition‐specific remodeling of tRNA pools [192, 256]. Integration of DRS with LC‐MS/MS and complementary epitranscriptomic assays further enables transcriptome‐scale mapping of structured noncoding RNAs and their dynamic modification states [192, 256, 257, 258].

Collectively, evidence across lncRNAs, circRNAs, tRNAs, and other structured or noncanonical RNAs demonstrates that nanopore DRS substantially expands the detectable transcriptome. By delivering full‐length transcript structures, native processing states, and modification landscapes that are largely inaccessible to conventional srRNA‐seq, DRS provides a comprehensive framework for exploring the functional diversity of noncoding and noncanonical RNA species in biology and disease.

### RNA structure and conformational heterogeneity

RNA structure is an intrinsic component of transcriptome diversity, shaping RNA stability, subcellular localization, and interactions with proteins and other RNAs. Classical approaches to RNA structure probing rely on chemical modification or enzymatic digestion and typically require dedicated experimental workflows that are separate from transcriptome profiling. DRS provides a complementary, signal‐based strategy by capturing ionic current and dwell‐time variations that reflect RNA secondary and tertiary structure during translocation through the nanopore [259]. In DRS, a motor enzyme feeds native RNA molecules through a biological nanopore, and both the nucleotide sequence and its structural context influence the resulting ionic current and translocation kinetics.

Base‐paired regions, stable secondary structures, and chemical adducts used for structure probing can all perturb current intensity and dwell time in characteristic ways. Several proof‐of‐concept studies have demonstrated the feasibility of this approach. For example, the nanoSHAPE framework showed that SHAPE adducts and endogenous modifications in rRNA generate reproducible current and dwell‐time shifts at single‐molecule, long‐read resolution, enabling simultaneous sequencing and structure probing [118]. In a complementary single‐molecule system, an engineered reverse transcriptase coupled to an MspA nanopore revealed that enzyme stepping kinetics are sensitive to downstream RNA secondary structure, allowing direct detection of structured regions without prior cDNA conversion [260]. Related strategies exploiting dwell‐time perturbations at helicase “brake” points have further been used to distinguish Ψ from uridine and to infer modification‐dependent structural stabilization in viral and bacterial RNAs [261, 262]. Collectively, these studies indicate that structured or base‐paired RNA regions produce distinct signal signatures compared with flexible, unstructured segments, although deconvolving structural effects from those of chemical modifications remains an active area of methodological development [118, 260]. Because DRS yields full‐length reads, structure‐sensitive signals can, in principle, be mapped continuously along entire transcripts and analyzed in conjunction with AS patterns, untranslated regions, and epitranscriptomic modifications [263, 264, 265, 266]. In this way, DRS‐based approaches complement transcriptome‐wide chemical and enzymatic structure‐mapping methods by embedding structural information within native transcript architectures.

RNA structure plays a central role in regulating AS, RNA‐protein interactions, and repeat‐associated toxic gain‐of‐function mechanisms, and its disruption contributes to cancer, neurodegeneration, and repeat‐expansion disorders [267, 268, 269]. Disease‐associated single‐nucleotide variants can act as riboSNitches, altering local or long‐range RNA structure and thereby reshaping splicing decisions, RNA stability, or RNA‐binding protein affinity [265, 266]. Integrating DRS‐derived structural signals with isoform‐resolved modification maps and transcript profiles offers a promising strategy to pinpoint variant‐ or isoform‐specific structural changes that rewire posttranscriptional regulation and translational control in disease [264, 270, 271]. sm‑PORE‑cupine integrates chemical probing with direct RNA sequencing to map RNA structure ensembles at single‑molecule resolution. By uncovering isoform‑specific structural heterogeneity and linking these ensembles to translation efficiency and RNA stability, this approach provides valuable insights into RNA structure‐function relationships across complex transcriptomes [272].

Taken together, current evidence supports nanopore DRS as a promising, though still maturing, signal‐based complement to chemical and enzymatic RNA structure probing. Its ability to relate RNA structural features to full‐length isoforms, chemical modifications, and disease‐linked sequence variation on single molecules positions DRS as a promising emerging tool for studying RNA conformational heterogeneity in health and disease. Although systematic applications in plant systems remain limited, this capability holds significant promise for broader implementation.

### Identification of fusion and TE‐related transcripts

Fusion transcripts can arise from readthrough transcription, transposable elements (TEs) mediated rearrangements, structural variation, or rare *trans*‐splicing events, and second‐generation sequencing studies have already reported the presence of hundreds of thousands of fusion transcripts across diverse species [273, 274]. cDNA‐based RNA‐seq is prone to artificial chimeras generated during RT, complicating interpretation. By eliminating RT and PCR, DRS reduces one major source of fusion artifacts and enables direct inspection of breakpoint‐spanning native RNA molecules [202]. At the same time, long‐read fusion detection remains sensitive to mapping errors and annotation gaps, particularly in repetitive plant genomes. Accordingly, fusion transcript identification using DRS should be interpreted with caution and supported by orthogonal evidence [275]. In plant systems, a promising direction is to link fusion‐like transcripts to TEs activity and genome evolution, rather than treating them solely as isolated anomalies. Analysis in *A. thaliana* has revealed that intragenic TEs can function as regulatory modules that are co‐transcribed with host genes, and their epigenetic states modulate RNA polymerase II elongation as well as the usage of APA signals embedded within TE sequences, thereby shaping the production of alternative TE‐gene transcript isoforms [276].

Together, these applications define a DRS‐centered atlas of RNA biology. By enabling joint observation of RNA modifications, isoform architecture, transcript termination, noncanonical RNA species, structural signals, TE‐associated diversity, and fusion events, DRS establishes a coherent foundation for integrative transcriptome analysis.

## TYPICAL APPLICATION SCENARIOS AND BREAKTHROUGH DISCOVERIES OF DRS TECHNOLOGY

### Viral and pathogen transcriptomics

DRS has emerged as a useful technology for studying viral and pathogen transcriptomes [277, 278, 279]. Unlike conventional cDNA‐based sequencing approaches, DRS enables the sequencing of full‐length native RNA molecules without RT or amplification, thereby preserving strand information, transcript structure, and RNA modification signatures [189]. These features have facilitated new analyses for understanding the transcriptional complexity, epitranscriptomic regulation, and rapid detection of infectious agents.

Central to these processes is the recognition that RNA modifications constitute an important regulatory layer in both host and viral gene expression (Figure 4) [280]. Nanopore DRS enables native, full‐length RNA reads while capturing modification‐associated perturbations in ionic current, providing a complementary alternative to short‐read, antibody‐ or chemistry‐based mapping that typically fragments molecules and loses isoform linkage [189, 281]. Using DRS, RNA modifications have been extensively characterized across transcripts from diverse viral genomes, encompassing both DNA viruses (e.g., adenovirus) and RNA viruses such as SARS‐CoV‐2, HIV, hepatitis B virus (HBV), bamboo mosaic virus (BaMV), and cucumber mosaic virus (CMV) [282, 283]. In 2020, Price et al. utilized nanopore DRS to demonstrate that m^6^A modifications on adenovirus RNAs are essential for regulating the splicing efficiency of viral late transcripts, highlighting a critical role for epitranscriptomic regulation in viral gene expression [184]. Studies on SARS‐CoV‐2 virus uncovered that m^6^A modifications are enriched in the 3′ end of the viral genome [284, 285]. Two studies on HIV have revealed isoform‐dependent methylation patterns and unique 2‐LTR transcript modifications, and identified several critical m^6^A sites near the 3′ end of the viral RNA, which are essential regulators of normal HIV‐1 RNA splicing and protein translation [286, 287]. Applying nanopore DRS to HBV transcripts, researchers identified four high‐confidence m^5^C sites, demonstrating that HBV mRNAs are extensively m^5^C‐modified and underscoring the power of DRS for precise, transcript‐resolved viral epitranscriptome mapping [288]. DRS has also been applied to detect other modifications such as Ψ [289, 290] and Nm [291] on viral RNAs. However, integrative studies combining DRS with complementary approaches have revealed the absence of significant m^6^A modification sites in certain RNA viruses. This includes mosquito‐borne viruses such as chikungunya (CHIKV) and dengue (DENV), as well as plant‐infecting viruses like zucchini yellow mosaic virus (ZYMV) and trichosanthes mottle mosaic virus (TrMMV) [292, 293]. Overall, the sites and frequency of RNA modifications in viruses are relatively low, and future studies will require more experiments to further confirm the functions of these potential modifications in virus infection. Besides virus, DRS has also been used to detect RNA modifications in other pathogens. Using a modification‑free in vitro transcription (IVT) sample as a negative control, Tan et al. applied nanopore DRS to enable transcriptome‑wide RNA modification detection in *Escherichia coli* and *S. aureus* [208].

In addition to epitranscriptomic profiling, the long‐read nature of nanopore sequencing enables modification detection in the context of full‐length transcripts, allowing researchers to link epitranscriptomic marks with specific isoforms, subgenomic RNAs, or transcriptional variants [287, 294]. This integrative view is particularly valuable for RNA viruses with compact and multifunctional genomes.

Many viruses exhibit highly complex transcriptional strategies, including nested transcription, overlapping open reading frames, and extensive use of subgenomic RNAs [295]. Nanopore DRS, with its ability to produce full‐length native RNA reads, has substantially advanced the discovery of viral RNA structural variants. For example, nanopore DRS of SARS‐CoV‐2 revealed a highly complex transcriptome containing numerous previously unannotated subgenomic RNAs, alternative transcription regulatory junctions, and noncanonical fusion transcripts [278, 279]. Similar approaches applied to herpes simplex virus 1 uncovered extensive alternative transcription start and PAS, as well as read‐through and antisense transcription, highlighting unexpected transcript isoform diversity during infection [296]. Other researchers have also uncovered previously unannotated transcript isoforms, alternative transcription start and termination sites, and read‐through transcription events in diverse RNA viruses [51, 278, 297].

Beyond isoform discovery, DRS enables strand‐specific quantification of genomic and antigenomic RNAs, providing insights into replication and transcription dynamics in positive‐ and negative‐sense RNA viruses [278, 296]. In segmented or recombination‐prone viruses, long reads help resolve chimeric RNAs and fusion transcripts that may play roles in viral evolution or pathogenicity [296, 298]. Together, these studies demonstrate that nanopore DRS is particularly suited to resolving the structural complexity of viral transcriptomes, providing an integrated view of RNA architecture that is often inaccessible to short‐read sequencing technologies. In many clinical or environmental samples, pathogen‐derived RNA represents only a small fraction of total RNA, posing challenges for direct sequencing [299, 300]. To address this, targeted and enrichment strategies have been developed to enhance the representation of pathogen transcripts in DRS libraries.

Host RNA depletion strategies, such as ribosomal RNA removal, poly(A) selection, and size selection, can substantially reduce the overwhelming background of host‐derived transcripts, thereby increasing the relative abundance of pathogen RNAs in sequencing libraries [208, 301]. Adaptive sampling provides an additional, software‐controlled enrichment approach unique to nanopore sequencing, in which short stretches of signal from a molecule are analyzed in real time and compared to reference sequences; reads matching target pathogen genomes are accepted and allowed to continue sequencing, whereas non‐target (e.g., host) molecules are rejected by reversing the voltage and ejecting them from the pore, thereby enriching pathogen‐derived transcripts during the run [302]. Experimental evaluations using synthetic mock communities demonstrated enrichment of rare species by up to ~14‐fold under optimal long‐read conditions, with an effective enrichment of ~5‐fold after accounting for yield losses due to read rejection [303]. This allows selective enrichment of pathogen‐derived molecules during the sequencing run itself, improving efficiency and reducing sequencing costs. Except for ONT's built‐in adaptive sampling strategy, several computational tools have been developed to implement adaptive sampling in real time [304, 305]. Signal‐based methods such as UNCALLED [306] and Sigmap [307] directly compare raw current traces with reference‐derived signal models to rapidly classify molecules before full sequencing. Sequence‐based approaches including Readfish [308], ReadBouncer [309], and RUBRIC [310] first perform ultra‐fast basecalling and then align short initial sequence fragments to reference genomes to decide whether to retain or reject a read. SquiggleNet [311] employs convolutional neural networks trained directly on raw nanopore electrical signals to classify molecules as target or non‐target in real time, without requiring basecalling or full sequence alignment. Together, these tools provide flexible strategies for pathogen enrichment and targeted sequencing.

Despite its versatility, ONT adaptive sampling faces several limitations in transcriptomic applications. The relatively short length of mRNA molecules reduces the potential benefit of early read rejection, while delays in basecalling and alignment often mean that a substantial portion of each molecule is sequenced before a decision is made [304, 312]. Frequent rejection events can also decrease pore utilization efficiency and overall sequencing yield [303].

One of the most important advantages of nanopore sequencing is its capacity for real‐time data generation [313]. Nanopore DRS enables rapid detection of RNA pathogens directly from clinical or field samples, with sequencing and analysis occurring concurrently. This capability has been demonstrated in studies where near‐complete viral genomes and strain‐level information were obtained within hours of sequencing, such as DRS of porcine reproductive and respiratory syndrome virus (PRRSV) from clinical samples, enabling accurate strain discrimination and detection of co‐infections [314]. Real‐time sequencing allows early identification of viral species and strains, often before a sequencing run is completed [315]. In addition, because DRS preserves native RNA molecules, it offers the unique potential to detect RNA modifications alongside sequence information, raising the possibility of monitoring epitranscriptomic signatures associated with virulence, host adaptation, or antiviral resistance [316]. As portable nanopore platforms continue to improve, DRS‐based workflows are becoming increasingly feasible in decentralized or resource‐limited settings. This opens new opportunities for on‐site pathogen surveillance, rapid outbreak monitoring, and integration of transcriptomic and epitranscriptomic data into public health decision‐making [317, 318, 319].

### Bacteria, archaea, and metatranscriptomes

DRS has so far been less widely applied to bacteria, archaea, and metatranscriptomes, largely because commercial kits compatible with nonpoly(A) RNA molecules are not available. With recent technological advances, however, this approach is emerging as a powerful approach for dissecting bacterial, archaeal, and community‐level metatranscriptomes, enabling information to be retrieved across multiple layers of biological regulation (Figure 4).

In prokaryotes, one major application of DRS is the single‐molecule, full‐length characterization of primary transcript architectures. In *Vibrio parahaemolyticus*, enzymatic poly(A) tailing followed by DRS enabled comprehensive mapping of TSS, TTS, and operon structures, revealing that many internal and antisense TSSs actually arise from overlapping genes and quantifying highly complex combinations of transcriptional units within individual operons, thereby refining annotations derived from srRNA‐seq [320]. Functionally, this strategy has been used to resolve antibiotic resistance repertoires in *Klebsiella pneumoniae*, simultaneously profiling the content of plasmid‐borne resistance genes and their instantaneous expression levels; within ~10 h, expression signals for ≥35% of resistance genes can be detected and multiple co‐transcribed operons composed of resistance determinants (e.g., *rmtB‐blaTEM‐1* and *aac(6′)‐Ib‐cr‐blaOXA‐1‐catB4*) can be delineated [321]. In *E. coli*, native RNA reads uncovered extensive heterogeneity in TSSs, TTSs, 5′/3′ UTR and operon organization, and revealed a diversity of transcript isoforms far exceeding that reconstructed from short‐read data [191, 208]. In the plant pathogen *Dickeya dadantii*, virulence‐associated genes are frequently embedded within complex, condition‐dependent transcriptional units whose composition and boundaries shift markedly in response to environmental cues [322]. In *S. aureus*, native RNA reads enabled construction of a “discontinuous operon atlas,” identifying numerous operons that are split, interlaced, or rearranged across distant loci, thereby challenging the traditional view of strictly continuous operons [323]. Comparative analyses across multiple bacterial and archaeal species further indicate that archaea commonly exhibit abundant leaderless mRNAs, atypical initiation and termination patterns, and irregular transcriptional units, features that are difficult to reconstruct from short reads [324].

DRS is also well‐suited for interrogating rRNA and tRNA modifications and their dynamic remodeling under environmental stress. Early work on 16S rRNA demonstrated that it can distinguish canonical from modified nucleotides within native 16S molecules, providing a direct, signal‐level readout of modification status [325]. A subsequent systematic analysis of *E. coli* ribosomes used DRS to resolve 17 distinct RNA modifications across 36 positions, enabling simultaneous detection of multiple modified sites in both the 30S and 50S subunits [262]. Antibiotic‐challenge experiments further showed that ribosome‐targeting drugs trigger pronounced loss and rewiring of modifications around the A‐ and P‐site regions, which appear in DRS data as characteristic “modification‐loss fingerprints” [326]. Heat shock and other perturbations induce broad remodeling of rRNA and tRNA modification landscapes in *E. coli*, and many mRNA modifications previously reported by antibody‐enrichment approaches may in fact be substantially less prevalent than initially proposed [208, 327, 328].

Beyond rRNA, DRS has enabled single‐molecule dissection of tRNA structure, processing and modification. Direct nanopore sequencing of individual full‐length tRNAs has shown that even highly structured, heavily modified tRNAs can be read continuously from 5′ to 3′, with both sequence and modification information retained within the same molecule. This proof‐of‐concept established a technical foundation for functional studies, demonstrating that isoacceptor and isodecoder tRNAs can be distinguished on the basis of their molecule‐specific modification signatures [36]. Given that archaeal rRNAs and tRNAs often harbor exceptionally dense and chemically diverse modifications, full‐length native reads provide a unique opportunity to track how modification patterns mature over time and how they contribute to archaeal adaptation to extreme environments [329]. In *Pseudomonas aeruginosa* clone C, DRS captured condition‐dependent transcriptional programs for virulence factors, resistance determinants and metabolic regulators within a single experiment [330]. In *P. aeruginosa*, tRNA hydroxylation has been identified as a key epitranscriptomic regulator of metabolic state and, consequently, pathogenicity; full‐length, modification‐preserving tRNA reads from DRS allow specific modification combinations to be directly linked to virulence phenotypes [331]. In *E. coli*, integration of NAD tagSeq II with DRS revealed pronounced growth‐stage‐dependent changes in NAD^+^ capping across the transcriptome, suggesting that this redox‐linked 5′ modification acts as a molecular sensor of metabolic status and growth dynamics [332]. Likewise, RNA‐mediated immune pathways can be directly monitored by DRS. For example, in an RNA‐activated Cas12a3 defense system, Cas12a3 cleaves tRNA tails to execute antibacterial immunity, and the resulting cleavage and processing patterns are detectable [333]. Collectively, these studies illustrate how it connects RNA modifications and processing events to core functional phenotypes such as virulence, stress tolerance, and immunity in bacteria and archaea. At the community level, DRS has been applied to metatranscriptomic and clinical settings. The DEMINERS framework increases the throughput and accuracy of DRS data, enabling comparative transcriptome analyses of complex clinical specimens and improving both the sensitivity and resolution of pathogen detection and expression profiling [334]. In food safety, combining direct metatranscriptomics with multiplex RT‐PCR amplicon sequencing allows simultaneous detection of viable pathogens within complex food matrices, with DRS providing direct evidence for pathogen presence and active expression of virulence genes [335]. In environmental studies, optimization of RNA extraction protocols for nanopore DRS has enabled soil metatranscriptome profiling from highly inhibitory matrices, facilitating characterization of active microbial consortia and their expressed functional pathways [336]. In marine ecosystems, DRS‐based metatranscriptomics of planktonic crustacean communities has revealed seasonal shifts in community composition and functional gene expression, underscoring the potential of DRS for time‐series monitoring of active microbiomes in situ [337]. At present, modification analyses in metatranscriptomic DRS are still largely confined to global trends or abundant rRNA/tRNA species, and precise mapping of low‐abundance mRNA modification sites remains challenging.

Finally, standardized protocols for preparing native and unmodified bacterial RNA, together with matched IVT controls, have provided a robust experimental and analytical foundation for these studies [301]. Overall, the application of DRS in prokaryotic systems and their metatranscriptomes is rapidly evolving from purely structural transcriptomics toward integrated epitranscriptomic, functional, and ecological analyses.

### Plant development and environmental responses

Plant growth, development, and environmental adaptation rely on tightly coordinated regulation across multiple layers of RNA biology, including transcript structure, alternative processing, polyadenylation, RNA modification, and the activity of noncoding RNAs [338, 339]. Historically, these layers have been investigated using distinct experimental strategies, mainly srRNA‐seq for expression and splicing, 3′‐end sequencing for APA, and antibody‐based approaches for RNA modifications, resulting in fragmented data sets that lack molecular linkage [340]. Consequently, it has remained difficult to determine whether different RNA regulatory features co‐occur on the same transcript molecules or act independently within a given developmental or stress context.

DRS provides a technical advance by enabling the simultaneous interrogation of multiple RNA features on native RNA molecules. In many plant species, this integrative capacity has begun to reveal coordinated RNA regulatory states that were previously inaccessible [52, 341]. A representative example comes from rice, where DRS‐based analyses jointly profiled m^6^A and m^5^C modifications across multiple tissues [33]. 3389 to 14,499 modified transcripts in different tissues carrying both modifications were detected, indicating widespread co‐occurrence of epitranscriptomic marks that had previously been mapped separately. Importantly, these modifications were not uniformly distributed across all transcript isoforms of a gene; instead, specific isoforms, often defined by alternative 3′ ends or terminal exon usage, exhibited preferential modification, suggesting a tight coupling between RNA processing and epitranscriptomic regulation. In *A. thaliana*, direct sequencing of native RNAs revealed that m^6^A‐modified transcripts frequently display distinct poly(A) tail length compared with unmodified transcripts, particularly under stress conditions [52]. Beyond protein‐coding RNAs, DRS has extended multilayer analyses to lncRNAs. 1149 novel lncRNAs from *A. thaliana* and their m^6^A modification and poly(A) tail features have been simultaneously profiled, revealing reduced methylation levels relative to protein‐coding RNAs, a positive role of m^6^A in lncRNA abundance, and a length‐independent contribution of poly(A) tails to lncRNA stability [210]. These studies illustrate how DRS expands plant RNA research from parallel analyses of isolated features into an integrated view of coordinated RNA states. Nanopore DRS enables mechanistic insights into how plants fine‐tune gene expression during development, forming the foundation for problem‐driven discoveries in plants.

Plants are continuously exposed to fluctuating environmental conditions and pathogen challenges, requiring rapid and reversible reprogramming of gene expression [342]. While transcriptional induction and repression under stress have been extensively characterized using srRNA‐seq, accumulating evidence indicates that stress adaptation in plants relies heavily on posttranscriptional regulation [217, 342]. A major limitation of conventional approaches is that these regulatory layers are typically assayed separately, making it difficult to determine how they are coordinated on the same RNA molecules during stress responses.

DRS has begun to address this challenge by enabling integrated analyses of multiple RNA features in stressed plant tissues. In *A. thaliana*, DRS‐based profiling under salt stress revealed widespread remodeling of both mRNA processing and RNA modification landscapes [52, 269]. These studies showed that stress‐responsive genes frequently exhibit coordinated changes in APA, isoform usage, and m^6^A modification, rather than isolated alterations in any single layer. Importantly, m^6^A‐modified transcripts displayed distinct stability and protein output profiles under stress, linking epitranscriptomic regulation directly to physiological outcomes [343]. Similar multilayer regulatory patterns have been observed in other crop species [344, 345]. In maize roots exposed to short‐term salt stress, DRS revealed extensive stress‐induced transcriptome reorganization, including 2223 differentially expressed isoforms, reduced poly(A) tail length, and decreased m^5^C signals [346]. These findings highlighted that stress responses in crops are not limited to changes in gene expression levels but involve coordinated reconfiguration of transcript structures and chemical modifications, which may contribute to stress tolerance mechanisms. DRS has also provided new insights into the role of RNA regulation in biotic stress responses. Research from *A. thaliana* and apple demonstrated that m^6^A modification is essential for resistance against pathogens, with stress‐induced changes in RNA modification patterns affecting the expression and processing of defense‐related transcripts [347, 348]. Notably, these studies linked RNA modification status to specific transcript isoforms and processing outcomes, suggesting that epitranscriptomic regulation fine‐tunes immune responses at a resolution beyond gene‐level expression.

TEs are a defining feature of plant genomes, often accounting for the majority of genomic content in crop species [349]. Beyond their well‐established roles in genome evolution and structural variation, increasing evidence suggests that TEs actively shape transcriptome diversity and stress responsiveness [350, 351]. However, dissecting TE‐derived transcriptional regulation has long been challenging, as srRNA‐seq struggles to resolve TE‐associated transcript isoforms due to sequence repetitiveness, ambiguous read mapping, and fragmented transcript reconstruction [202]. By directly capturing full‐length native RNA molecules spanning gene‐TE junctions, DRS has revealed how TEs are transcriptionally integrated into host gene architectures. A landmark example comes from the cotton genus (*Gossypium*), whose genomes are characterized by massive TE expansion and frequent polyploidization. Tian et al. demonstrated that TEs exert a widespread and systematic impact on posttranscriptional regulation across eight cotton species [352]. Specifically, TE‐associated isoforms were found to be pervasive within a substantial proportion of expressed genes, harboring distinct TE‐derived features, including alternative terminal exons and intragenic TE insertions that create new alternative splice sites. Notably, TE‐driven turnover of splice sites and regulatory sequences may have contributed to regulatory divergence following polyploidization in cotton, with 5255 genes showing lineage‐specific divergence at the splicing level. Complementary insights have been obtained in *A. thaliana*, where DRS demonstrated that intragenic TEs can act as regulatory modules that are co‐transcribed with host genes, and that their epigenetic states influence RNA polymerase II elongation and the usage of APA signals embedded within TE sequences [276]. These findings provide a mechanistic framework for understanding how TE‐derived regulatory elements are conditionally activated, particularly in plants, where a sessile lifestyle and constant exposure to environmental and biotic stresses impose strong selective pressure for stress‐responsive regulatory plasticity [353].

Allele‐specific regulation is a pervasive yet underexplored layer of gene control in plants, particularly in hybrids and polyploids where multiple parental or subgenomic copies coexist within the same nucleus. In these systems, phenotypic variation, heterosis, and environmental adaptation often arise not simply from differences in gene presence or absence, but from allelic divergence in expression level, RNA processing, and post‐transcriptional regulation [354, 355]. However, resolving such allelic complexity has long been challenging, as srRNA‐seq lacks sufficient haplotype context to confidently assign reads to specific alleles, especially in highly similar homoeologous regions. Beyond expression quantification, DRS has revealed that allelic divergence frequently extends to transcript structure and RNA processing. In allotetraploid *B. napus*, Li et al. uncovered extensive subgenome‐specific AS and APA, with thousands of homoeologous gene pairs exhibiting asymmetric isoform usage across tissues [356]. Similar patterns have been observed in other polyploid systems, where one allele preferentially produces truncated or extended transcript isoforms, suggesting that posttranscriptional regulation contributes to functional divergence of subgenome following polyploidization [357].

Long‐read analyses of panicle development in hybrid rice illustrate the power of full‐length transcript profiling to resolve allele‐dependent APA landscapes. Using Iso‐seq, Wu et al. showed that ~80% of expressed genes harbor multiple poly(A) sites and that APA patterns differ between hybrids and parental lines, with shortened 3′ UTRs associated with increased expression and miRNA‐mediated regulation during spikelet development [358]. While not based on DRS, these findings highlight how long‐read approaches uncover heritable APA regulation that is largely inaccessible to short reads. Importantly, DRS could further extend such analyses by coupling accurate APA detection with allele‐specific RNA modification profiling on the same native transcripts, opening new avenues for dissecting multilayer allelic regulation in plants. Recent nanopore DRS studies in human and mouse systems have already demonstrated the feasibility of resolving allele‐specific m^6^A modification patterns at single‐molecule resolution [359], providing a proof‐of‐principle for extending similar epitranscriptomic analyses to plant transcriptomes.

Beyond descriptive transcriptome analyses, DRS has increasingly been applied to resolve concrete molecular mechanisms underlying plant development, with flowering‐time control in *A. thaliana* serving as a paradigmatic example. Flowering integrates environmental cues and endogenous signals through multilayered regulation involving transcription, chromatin state, RNA processing, and RNA modification. Many key regulators in this pathway, such as FLC, FCA, FPA, and antisense transcripts like COOLAIR, are controlled predominantly at the post‐transcriptional level, rendering them particularly amenable to DRS‐based interrogation [360, 361].

Early DRS studies in *A. thaliana* revealed extensive heterogeneity in RNA 3′ end formation and transcript termination among flowering‐related genes, uncovering APA events that were previously obscured by srRNA‐seq [52]. Subsequent work demonstrated that RNA‐binding proteins such as FPA promote premature transcription termination at specific loci, including nucleotide‐binding leucine‐rich repeat (NLR) genes and flowering regulators, thereby reshaping transcript architecture and downstream gene activity [362]. Further, DRS has enabled direct interrogation of RNA modification in flowering control. In the seedlings and inflorescence tissues of *A. thaliana*, DRS‐based analyses showed that m^6^A deposition is enriched near stop codons and 3′ UTRs of flowering regulators, and that perturbation of m^6^A writers or readers leads to delayed flowering phenotypes [363, 364]. Crucially, DRS revealed isoform‐specific m^6^A patterns, linking alternative RNA processing directly to epitranscriptomic regulation. By tracing how RNA processing and modification converge on specific regulatory genes, DRS enables a transition from global transcriptome surveys to mechanism‐driven insights.

Taken together, studies across plant development and environmental responses highlight important advances enabled by nanopore DRS, whereby transcriptome analysis is no longer confined to parallel measurements of isolated RNA features but can instead capture coordinated RNA states on individual native molecules. DRS has revealed how multiple regulatory layers are jointly remodeled during development, stress adaptation, and genome evolution. In this sense, DRS functions not merely as a profiling technology, but as an analytical framework that links RNA‐level coordination to phenotype‐relevant mechanisms in complex plant systems.

### Animal models and organ development research

Understanding organ development requires a comprehensive dissection of transcriptional regulation across multiple aspects, including gene expression dynamics, isoform choice, RNA processing (AS and APA), and post‐transcriptional modifications. The resolution of information from these regulatory layers is constrained by the underlying RNA sequencing technology. Traditional srRNA‐seq has transformed developmental biology; however, it relies on indirect reconstruction of transcript structures and often fails to resolve complex isoforms, repetitive regions, and long‐range coupling between exons and 3′ UTR. In addition, RT and PCR introduce biases that obscure native RNA features. Nanopore DRS provides an alternative transcriptomic approach to address many of these limitations [9, 35, 51, 56, 325].

Animal models are particularly well‐suited for DRS‐based analyses. Developmental staging in organisms such as *C. elegans*, zebrafish, and mice allows time‐resolved mapping of isoform programs. Genetic perturbation and tissue‐specific sampling support inference linking developmental phenotypes to AS or APA, with structural transcript changes directly observable in vivo using DRS. Moreover, in organs with high isoform complexity (brain, heart, immune organs), DRS complements single‐cell and short‐read atlases by providing full‐length transcript context, including isoform‐resolved regulatory features such as epitranscriptomic signatures and tail dynamics.

A landmark demonstration of DRS in developmental biology is the full‐length DRS analysis across *C. elegans* developmental stages, which reveals extensive unannotated isoforms and unexpectedly high transcriptome complexity [201]. This work illustrates several key advantages of DRS in development studies. First, DRS identifies novel isoforms that are specific to embryonic, larval, or young adult stages. Second, full‐length coupling of DRS links splicing patterns to 3′ ends for analysis of developmental regulation of UTRs. Third, refined transcript annotations enable isoform‐level hypotheses in developmental genetics. Beyond development, DRS time‐course studies in *C. elegans* aging provide a methodological template for longitudinal DRS analyses of developmental organ maturation, as such longitudinal designs reveal coordinated transcriptomic remodeling across isoform usage, tail dynamics, and epitranscriptomic regulation [365].

Embryogenesis involves rapid cell fate decisions and dynamic AS. Long‐read sequencing studies of developmental stage transitions identify unannotated isoforms and stage‐dependent splicing within full‐length data [366]. Early zebrafish development also undergoes dramatic changes in polyadenylation and translation during the maternal‐to‐zygotic transition. Combining DRS with polysome or ribosome profiling directly links poly(A)/UTR features to translational output. DRS is particularly powerful for mapping isoform switching during organogenesis (heart tube formation, neurogenesis, hematopoiesis); defining stage‐specific patterns of 3′ UTR remodeling associated with translational control; and characterizing RNA modifications during developmental transitions.

DRS is now widely applied in the study of mammalian tissues, including multi‐organ surveys. An example integrates nanopore DRS to map tissue‐specific RNA landscapes across mouse organs under metabolic perturbations, illustrating the breadth of in vivo DRS readouts at the levels of isoforms, expression, and RNA metabolism [367]. Similarly, a porcine multitissue study with fetal samples profiles transcript isoforms and the m^6^A landscape across tissues using DRS [368], demonstrating that DRS can scale to complex mammalian physiology and support comparisons between fetal and adult tissue programs [369]. For developmental neurobiology, DRS approaches are important because many regulatory RNAs in neurogenesis and synaptogenesis are not strictly polyadenylated or are expressed in isoforms with complex ends. Therefore, expanded DRS can provide a more comprehensive view of the regulatory RNA landscape during brain development [370]. Using DRS across multiple mammalian species and an avian outgroup, a recent study revealed that a small subset of evolutionarily conserved isoforms accounts for most gene expression, while the majority of isoforms are species‑specific and lowly expressed. The identification of conserved and isoform‑specific m^6^A deposition, along with widespread coordinate splicing, highlights the evolutionary importance of epitranscriptomic regulation in maintaining functional transcript diversity and buffering transcriptome variation [371].

Developmental regulation often relies on the process of transcript maturation rather than transcript abundance. DRS captures transcript maturation through long reads spanning splice junctions and PAS, with extensions to nascent or chromatin‐associated RNA. K. Choquet et al. employed the DRS approach in 12 human lymphoblastoid cell lines (LCLs), and identified allele‐specific splicing patterns that are regulated by genetic variants, such as splice‐site SNPs and distal intronic variants [370]. Notably, HLA class I genes exhibited frequent allele‐specific splicing orders that co‐occurred with AS and APA events, illustrating the complexity of RNA maturation regulation in immune‐related loci [370]. In addition, the same study described genetic regulation of nascent poly(A) tail lengths in chromatin‐associated RNA (including large allele‐specific differences for *ERAP2*), suggesting a connection between polyadenylation dynamics and transcript abundance [370]. In developmental immunology and organ maturation, such allele‐dependent programs may tune transcript timing, isoform choice, and abundance in a lineage‐ and stage‐specific manner.

Vertebrate embryo studies in zebrafish and *Xenopus laevis* show that DRS demonstrate stage‐specific isoform switching and poly(A)‐tail dynamics during embryogenesis, supporting a role for posttranscriptional regulation in major developmental transitions [114]. In combination with modification profiling [372, 373], these studies indicate that DRS provides a unified view of isoform structure, processing, and epitranscriptomic state during development. Cardiac systems present a practical challenge because mitochondrial RNAs account for a large fraction of polyadenylated RNA in cardiomyocytes, masking lower‐abundance nuclear transcripts. Adaptive sampling has been combined with DRS to reduce mitochondrial RNA abundance in mouse heart tissue and hiPSC‐derived cardiomyocytes [194, 302]. This methodological adjustment improves detection of low‐abundance transcripts (e.g., *Ccl7*, *Rbm6*) and supports precise characterization of isoform expression and AS during cardiomyocyte differentiation [374, 375]. This integrated DRS‐based approach shows that selectively shifting the molecules sequenced can reveal previously undetected developmental regulators and improve mechanistic inference in organ development. Across model organisms and mammalian systems, DRS offers a broadly applicable framework for characterizing transcript maturation and post‐transcriptional regulation during organ development.

### Medicine and disease research

Human diseases increasingly reflect dysregulated RNA processing rather than simple changes in gene expression. Alterations in splicing, polyadenylation, RNA modifications, stability, and translation collectively shape pathology. Nanopore DRS enables simultaneous analysis of full‐length transcript structure and epitranscriptomic features on single molecules.

AS drives transcriptomic diversity and is frequently dysregulated in disease, altering coding potential and RNA fate without changing gene expression. srRNA‐seq often misses complex or low‐abundance isoforms. DRS enabling integrated analysis of splicing architecture alongside multiple regulatory features on the same molecule [9, 218, 376, 377].

In biomedical contexts, DRS has proven useful for resolving disease‐associated splicing complexity. For example, DRS identified 32 distinct full‐length *BRCA1* isoforms, many harboring multiple coordinated exon‐skipping events, including co‐occurrence of known pathogenic deletions such as *Δ*9‐10 with *Δ*21 [378]. In inherited retinal dystrophies, DRS enabled direct detection and quantification of full‐length aberrant transcripts arising from cryptic splice sites, pseudoexon inclusion, and complex combinations of splicing defects, including low‐abundance isoforms that are difficult to resolve with srRNA‐seq [379]. In rare genetic disorders, integration of nanopore DRS or cDNA sequencing with genomic haplotypes has enabled allele‐resolved analysis, linking heterozygous splice variants to allele‐specific isoform expression, as demonstrated in McArdle disease [380]. More broadly, DRS‐based analyses have identified novel isoforms, differential transcript usage, and poly(A) tail variation as potential biomarkers in conditions such as sepsis [381].

Despite these advantages, several limitations remain. Nanopore sequencing error rates are higher than those of Illumina platforms, often necessitating customized bioinformatic pipelines and, in some cases, short‐read‐based error correction for precise splice junction and open reading frame annotation [197, 377, 382]. In addition, full‐length DRS reads typically miss a short segment at the extreme 5′ end (approximately 6−12 nt), meaning that accurate TSS mapping may require complementary assays. Quantification of isoform abundance, while steadily improving, is still less mature than gene‐level quantification from short‐read data, and hybrid experimental designs are therefore commonly employed [381, 382, 383].

APA is a widespread but underappreciated regulator of gene expression. By altering poly(A) site usage, APA reshapes 3′ UTR length and regulatory elements, influencing RNA stability, localization, and translation, and is frequently dysregulated in disease. DRS is uniquely suited to study APA because it sequences native RNA molecules from the 3′ end through the full poly(A) tail, generating a characteristic homopolymer signal that enables single‐molecule mapping of transcript termini and estimation of poly(A) tail length [35, 60, 226, 228, 384]. Genome‐wide DRS provides reproducible poly(A) tail measurements across a broad dynamic range, from a few nucleotides to ~400 nt in human cells, offering quantitative insight into 3′‐end regulation beyond short‐read capabilities. Related methods, including TERA‐seq and Nano3P‐seq, profile both polyadenylated and nonpolyadenylated RNAs while preserving native 3′ ends, showing high concordance with annotated APA sites. End‐capture nanopore strategies further resolve RNA processing and decay intermediates at single‐molecule resolution. By avoiding RT and PCR, DRS reduces protocol bias, especially valuable for variable‐quality clinical samples.

At the biological level, nanopore DRS and related end‐capture methods reveal extensive heterogeneity in poly(A) tail length and composition across transcript isoforms. Genome‐wide analyses show broad, isoform‐specific tail length distributions that correlate with RNA stability and decay during vertebrate embryogenesis [35, 384]. In human leukemia cells, DRS uncovered a negative correlation between poly(A) tail length and steady‐state RNA abundance and stability, challenging the simplistic view that longer tails universally confer increased stability and instead revealing pathway‐specific tail‐length regimes [60]. Integrative analyses across multiple data sets further demonstrate that both tail length and tail composition, including the presence of nonadenosine residues, regulate mRNA stability and translation in a context‐dependent manner, with short or heterogeneous tails often marking highly expressed and efficiently translated transcripts [226, 232, 234, 235, 236, 385].

Specialized nanopore methods such as Nano3P‐seq and PolyTailor explicitly quantify poly(A) tail composition, enabling detection of non‐A residues and mixed tails as additional regulatory layers [384, 386]. These features have functional consequences across diverse systems. In human cells, allele‐specific DRS has identified genetic variants that modulate poly(A) tail length in concert with splicing order and APA choice, particularly in HLA class I genes, linking 3′‐end regulation to interindividual differences in immune gene expression [370].

Despite progress, standard nanopore DRS enriches for poly(A)+ RNAs and typically requires tails >10 nt, potentially underrepresenting short‐ or tail‐less transcripts. Modified approaches, such as poly(I) tailing or Nano3P‐seq, improve coverage. Nevertheless, DRS enables profiling of native RNA termini and poly(A) tails, advancing understanding of APA and 3′‐end regulation.

RNA chemical modifications, including m^6^A, Ψ, and Nm, dynamically regulate RNA structure, stability, splicing, and translation. Studying these effects requires detection in native transcript context. Nanopore DRS provides a direct, signal‐based approach for isoform‐resolved epitranscriptomic analysis. As native RNA passes through a nanopore, each k‐mer produces a characteristic ionic current signature [34, 114]. Chemical modifications alter current intensity and dwell time, generating detectable deviations captured either as basecalling errors or through modeling of raw signal traces [34, 114, 186, 387].

A key advantage of DRS is preservation of full‐length native RNA molecules, allowing modification sites to be mapped within precise transcript contexts, including specific splice isoforms, UTRs, and overlapping transcripts [26, 114, 118, 120, 186, 256]. By maintaining linkage between modification and transcript architecture, DRS overcomes limitations of short‐read methods that fragment RNA and obscure isoform assignment [34, 118, 120, 240, 241, 277, 388]. The field is supported by growing resources and tools [26, 240]. Databases such as DirectRMDB compile DRS‐derived modification maps across species, while computational methods including Nanocompore, xPore, CHEUI, TandemMod, and ModQuant detect differential and multitype modifications, estimate stoichiometry, and assess co‐occurrence on single molecules [34, 119, 177, 182, 185, 389, 390]. These advances enable mechanistic analysis of RNA‐modifying enzyme perturbations underlying disease.

Collectively, by converting modification‐induced signal changes into single‐molecule calls on full‐length RNAs, nanopore DRS overcomes several limitations of conventional epitranscriptomic methods, enabling isoform‐resolved analysis of RNA modification pathways in health and disease.

Nanopore DRS enables integrated analysis of RNA regulation across multiple layers. AS, APA, and RNA modifications coordinately shape RNA fate, yet srRNA‐seq typically analyzes them separately and loses isoform‐level linkage between processing events [200, 391, 392, 393, 394, 395]. Mechanistically, AS and APA are tightly coupled: 3′ end processing influences exon selection, while splicing decisions reshape 3′ UTR architecture and poly(A) site choice, together modulating coding potential, mRNA stability, localization, and translation [237, 393, 396], and thereby influencing processes such as apoptosis, immune responses, and oncogenic signaling [200, 216, 391, 392, 394, 395, 397, 398]. RNA modifications further interact with AS, APA, and RNA‐binding proteins [399, 400, 401, 402, 403, 404]. Epitranscriptomic studies increasingly highlight that these modifications function within integrated regulatory networks rather than as isolated marks [400, 401, 402, 403, 404].

Recent work has highlighted long‐read and third‐generation sequencing technologies as essential for quantifying coupled AS‐APA events and for resolving full‐length transcript isoforms in which multiple processing decisions co‐occur [216, 237, 394]. By preserving the physical linkage between splice patterns, 3′ UTR architecture, and, uniquely in the case of DRS, native RNA modification states on the same molecule, these approaches enable direct investigation of coordinated regulatory programs that are largely invisible to fragmented short‐read data or mark‐specific assays [200, 216, 237, 394, 402, 403]. Such integrated readouts reveal how multiple layers of RNA processing are orchestrated on individual transcripts.

Dysregulation of AS, APA, and RNA modification contributes to diverse diseases, including cancer, cardiovascular and neurodegenerative disorders, and immune dysfunction [200, 216, 391, 392, 393, 394, 397, 398, 399, 401, 404]. Increasingly, studies emphasize integrative analyses that combine isoform usage, RNA processing, epitranscriptomic states, and functional outputs to define disease‐specific regulatory programs and therapeutic targets [200, 394, 399, 400, 402, 403]. Long‐read DRS embodies this systems‐level perspective, enabling isoform‐resolved analysis of coordinated RNA regulatory layers that are often rewired in pathological states.

Beyond structure and modification, RNA stability and translational efficiency are key endpoints of RNA regulation, with altered decay or translation reshaping protein output in disease. Studying these processes requires isoform‐specific resolution. DRS provides molecule‐level readouts of RNA turnover, including transcript integrity, modification patterns, and direct measurement of poly(A) tail length [60, 120, 226], enabling isoform‐resolved analysis of tail dynamics across genes and biological conditions.

Genome‐wide DRS analyses reveal noncanonical relationships between poly(A) tail length, RNA abundance, and stability. In human leukemia cells, shorter‐tailed transcripts were often more abundant and stable [60], challenging the traditional long tail equals stability model. Instead, RNA fate appears context‐dependent, shaped by specific tail‐length regimes and tail dynamics. Consistent with TAIL‐seq and related studies, poly(A) tail length and 3′‐end modifications influence both RNA decay and translational efficiency, directly linking 3′‐end regulation to protein output [60, 226, 232].

DRS‐based analytical frameworks, such as NanoTrans and DEMINERS, extend these insights by jointly quantifying transcript isoforms, poly(A) tail features, and RNA modification states, enabling comparative analyses across normal and disease conditions or in response to therapeutic perturbations [120, 206]. Application of epitranscriptomic callers to DRS data, including pum^6^a and RNANO, has further connected modification dynamics to altered RNA stability in cancer, exemplified by hypoxia‐regulated m^6^A demethylase activity in gastric tumors [405]. By integrating transcript structure and regulatory features at single‐molecule resolution, nanopore DRS reveals coordinated changes in RNA stability and translation that drive disease progression and therapeutic response.

In prostate cancer, Nm of rRNA is a key regulator of translation. EZH2 binds the Nm enzyme fibrillarin (FBL) and increases rRNA Nm, which enhances global and IRES‑dependent translation and supports cancer cell growth [406]. Besides mapping diversity, including detecting Nm sites in rRNA, nanopore DRS combined with the NanoNm machine‑learning tool showed that internal Nm on mRNA increases mRNA stability and expression, is linked to widespread 3′ UTR shortening, and thereby promotes prostate cancer progression [178, 291, 407]. Studies have focused on the regulatory role of METTL3‐mediated m^6^A modification in AS [408]. Most recently, Yi et al. revealed that the interplay between EZH2 and m^6^A regulatory pathways [409] using DRS.

For Breast cancer, in ER^+^ breast cancer models, Nanopore DRS has shown that METTL3‐dependent changes in m^6^A sites can be linked to pathways associated with sensitivity or resistance to endocrine therapy, thereby providing site‐level evidence for mechanisms of resistance [410]. Further work indicates that different molecular subtypes exhibit subtype‐specific patterns of m^6^A hypomethylation or hypermethylation, and gene‐engineering experiments have verified the DRACH motif‐dependent activity of *ALKBH5*, suggesting that m^6^A helps shape tumor heterogeneity [411]. Beyond m^6^A, long‐read approaches have also been applied to profile the m^5^C epitranscriptome of breast cancer mRNAs, and, together with gene editing, to explore its regulatory networks, thereby extending the use of DRS to multiple modification layers [412].

For Hematological malignancies, DRS is used to analyze the transcriptome and epitranscriptome of human leukemia cell lines [60]. In acute myeloid leukemia (AML), azacitidine (AZA) treatment induces remodeling of mRNA m^5^C and has led to the proposal of prognostic biomarkers [413]. After both AZA and venetoclax (VEN) therapy, a panoramic analysis of m^6^A alterations suggests that RNA modifications are both a mechanism and an indicator of therapeutic efficacy [414]. DRS has revealed a novel mechanism of immunotherapy resistance mediated by intron retention in the *CD19* gene and selective splicing of the 5′ UTR in the *CD20* gene [415, 416]. These findings highlight the ability of DRS to directly capture “structurally resistant transcripts.”

In nonsmall cell lung cancer, studies have linked aberrant m^6^A patterns to prognostic genes for risk stratification [417]. DRS can also perform single‐molecule methylation analysis of small RNAs in blood and identify lung cancer‐associated methylation patterns with diagnostic potential [418]. In colorectal cancer, DRS has enabled simultaneous profiling of RNA modifications and alternative splicing, uncovering tumor‑associated exon‑skipping events with biomarker potential. Loss of exon ENSE00001632812 in the *MYH11‑201* isoform was consistently observed in tumor tissues and validated in TCGA cohorts, supporting its diagnostic relevance. By integrating modification calling with splicing analysis, this approach provides a framework for identifying cis‑regulatory relationships between RNA modifications and aberrant splicing, highlighting the power of long‑read sequencing to dissect epitranscriptomic regulation in colorectal cancer [419]. In lung adenocarcinoma, *CMTR2* mutations lead to splicing defects and expose therapeutic vulnerabilities, highlighting the value of long‐read sequencing for dissecting splicing mechanisms [98].

Nanopore DRS can be used to resolve m^6^A modifications on noncoding RNAs, providing complementary validation alongside MeRIP‐seq [420]. Studies also show that the modification landscape correlates with tumor grade [421]. In addition, the lncRNA *CHROMR* is associated with patient survival, offering clues for potential biomarkers [422]. Combined with CRISPRi functional screening, these lncRNA candidates can be further advanced toward actionable therapeutic targets [423].

Moreover, Nanopore sequencing detected ribosomal protein‐associated m^5^C changes and linked them to oxidative stress, metabolic reprogramming, and immune responses [424]. In clear cell renal cell carcinoma, Nanopore sequencing has been used to integrate the m^6^A methylome with the transcriptome, and long‐read RNA sequencing of archived tissues has enabled the discovery of novel transcripts [425, 426]. In hereditary leiomyomatosis and renal cell carcinoma, it can directly capture cryptic fumarate hydratase (FH) splicing mutations [427]. DRS has been used to systematically map the dynamic landscape of m^6^A and m^5^C modifications in muscle‐invasive bladder cancer and to assess their prognostic value [428, 429]. Nanopore RNA assembly improves the completeness of the transcriptomic landscape and enables the discovery of novel functional transcripts [430]. Transcript‐level features suggest value for stratifying aggressive subtypes [431]. Prmt5‐deficient B cells exhibit increased RNA‐processing complexity and are associated with slowed colorectal tumor progression, indicating that RNA processing in immune cells can also influence the tumor ecosystem [432]. DRS is demonstrating strong multidimensional analytical power in pan‐cancer research. First, at the level of transcriptome architecture, it enables the construction of a single‐gene transcript atlas [433], identification of oncogenic gene fusions [434, 435] and tumor‐specific splicing variants [436]. Second, at the epitranscriptomic level, DRS can be used to call multiple RNA modifications in rRNAs [437], and lncRNAs [438, 439], and reveal their biological significance, including m^6^A [440], m^5^C, Ψ [441], and A‐to‐I [441, 442]. Nanopore‐based epitranscriptomic fingerprinting of rRNA Nm and related marks can distinguish normal tissues, tissue‐of‐origin, and tumor subtypes, revealing stable, cancer‐specific rRNA modification signatures that may serve as diagnostic biomarkers and inform ribosome‐targeted therapies [437], which can enable the discovery of novel cancer diagnostic biomarkers [443]. In addition, DRS can dynamically monitor post‐transcriptional regulatory features such as poly(A) tail [444, 445].

Nanopore DRS can bridge the chain from variant to isoform and then to phenotype. In human muscle cells, it can directly capture DUX4‐activated repeats and isoforms, thereby completing disease‐relevant transcript annotations [446]; in congenital myopathies, it enables transcript‐level validation of the pathogenicity of biallelic *DST‐b* variants [447]; and for suspected splicing variants, allele‐specific isoform analysis can be performed [380]. DRS can interpret the pathogenicity of *BRCA1* exon duplications that generate fusion transcripts [448].

In amyotrophic lateral sclerosis/frontotemporal dementia (ALS/FTD), DRS directly revealed aberrant cryptic polyadenylation resulting from TDP‐43 protein loss [449], which may be associated with METTL3 deficiency [450], and enabled the construction of an m^6^A modification atlas in motor neurons [451]. In Alzheimer's disease, DRS allows precise discrimination of disease subtypes [189]. In addition, DRS has been used to evaluate the impact of environmental toxins on the epitranscriptome of brain organoids [452], and to elucidate a mechanism whereby m^6^A modification regulates neuronal ferroptosis after intracerebral hemorrhage via *Vdac3* [453]. Researchers generated a full‐length transcriptomic atlas of abdominal aortic aneurysm and identified novel transcripts [454]. They also revealed an emerging role of m^6^A RNA modification in cardiovascular diseases [455]. In heart failure, dysregulation of hnRNPL affects AS [456]. In patients with sepsis, blood‐based DRS can be used to identify both co‐transcriptional and posttranscriptional disease biomarkers [381]. Studies have reported sex‐specific transcriptomic and epigenomic characteristics in fear learning similar to those observed in posttraumatic stress disorder [457].

Framing RNA regulation by molecular mechanisms rather than disease categories underscores DRS as a versatile platform. Core processes, splicing, polyadenylation, RNA modification, and stability, are recurrently disrupted across diseases, and DRS enables systematic, isoform‐resolved analysis in clinical samples, supporting cross‐disease comparisons and shared regulatory insights. DRS is positioned to become a key tool for dissecting RNA regulatory mechanisms and advancing precision medicine.

### Clinical and translational applications: RNA vaccines, RNA drugs, and diagnostic markers

The clinical relevance of nanopore DRS derives from its ability to capture multiple layers of RNA regulation with disease phenotypes [63]. By providing direct access to the epitranscriptome, DRS enables mechanistic insights into gene regulation and RNA function that are increasingly important for clinical diagnostics and therapeutic development [26]. In contrast to srRNA‐seq, which fragments transcripts and relies on computational reconstruction, DRS identifies disease‑specific epitranscriptomic signatures and pathogenic isoforms without PCR amplification [458], thereby reducing technical bias and improving biological interpretability [189].

These technical advantages position DRS as a powerful platform for RNA biomarker discovery across diverse disease contexts. In cancer biology, the RNA modification landscape is tightly linked to tumor initiation and progression, with modifications such as m^6^A modulating oncogenic signaling pathways and tumor immune evasion [459]. Beyond cancer, recent DRS studies in sepsis have identified co‑ and posttranscriptional biomarkers, including poly(A) tail length variation, RNA modification dynamics, and isoform usage patterns, providing deeper insights into disease states than traditional sequencing approaches [460]. Similarly, DRS has revealed multilayered epitranscriptomic remodeling in macrophages following *M. tuberculosis* infection, underscoring its diagnostic potential in infectious diseases [62]. Conventional RNA profiling methods are often limited by amplification bias and loss of native RNA features, whereas DRS offers a long‑read, amplification‑free alternative that preserves native RNA modifications and molecular heterogeneity, further expanding its utility for biomarker discovery and clinical applications [9, 461, 462].

Likewise, nanopore DRS enables comprehensive profiling of viral transcriptomes, such as SARS‑CoV‑2, revealing complex RNA isoforms and modification states that inform viral replication strategies and pathogenic mechanisms [279, 334]. More broadly, DRS is emerging as a transformative tool in clinical diagnostics by enabling direct detection of RNA modifications and post‑transcriptional regulatory features that serve as sensitive biomarkers across a wide range of diseases [442, 459, 463]. Continued advances in modification‑calling accuracy and stoichiometry estimation further enhance biomarker specificity and interpretability [381], particularly as disease‐associated alterations in RNA modification patterns often precede detectable changes in transcript abundance.

Beyond diagnostics, nanopore DRS has emerged as an enabling technology for RNA‑based therapeutics, particularly in the rapidly expanding fields of mRNA vaccines and immunotherapy. mRNA‑based strategies hold broad potential for infectious diseases, cancer vaccines, and personalized immunotherapies, in which patient‑specific neoantigens can be encoded into mRNA constructs to elicit tailored antitumor immune responses [464, 465, 466]. The relatively low production costs and manufacturing simplicity of mRNA therapeutics further support their potential for globally accessible treatment strategies [467]. However, the rapid clinical deployment of mRNA vaccines has created a critical need for high‑resolution technologies capable of assessing RNA quality, structural integrity, and modification states. DRS has proven particularly effective in this context by enabling direct, full‑length characterization of therapeutic RNA molecules, thereby supporting quality control, optimization, and regulatory evaluation of mRNA‑based therapeutics [468].

DRS enables direct evaluation of vaccine RNA by identifying truncated transcripts, poly(A) tail length heterogeneity, and synthetic nucleotide modifications, such as Ψ and m^6^A, that critically influence translation efficiency and immunogenicity [468]. These analytical capabilities underpin emerging frameworks for comprehensive mRNA vaccine quality control and batch‑to‑batch consistency testing [467, 468]. Moreover, the real‑time nature of nanopore sequencing facilitates longitudinal monitoring of RNA integrity and modification drift during storage, transportation, and deployment, representing a key capability for ensuring the reliability of global mRNA vaccine distribution pipelines [467].

In parallel with vaccine applications, a broad range of RNA‑based therapeutics, including mRNA drugs, antisense oligonucleotides (ASOs), siRNAs, and RNA‑editing constructs, require precise characterization of RNA stability, structure, and modification states, all of which can be directly assessed by nanopore DRS [460]. Among these features, m^6^A modifications play central roles in regulating cancer progression, immune homeostasis, metabolic disorders, neurocognitive function, and stem cell differentiation, underscoring their relevance for therapeutic RNA design and optimization [469, 470, 471, 472]. Beyond m^6^A, additional RNA modifications, including m^7^G, m^5^C, m^1^A, Ψ, and Nm, have been implicated in diverse disease pathways and represent promising targets for next‑generation RNA therapeutics [473, 474].

The development of mRNA‑encoded therapeutic antibodies by companies such as BioNTech and Moderna further illustrates the clinical potential of RNA‑based technologies, with multiple candidates currently in preclinical and clinical development targeting infectious diseases, cancer, and toxic exposures [475, 476, 477]. In this context, DRS provides a multidimensional view of RNA therapeutic performance and quality [478]. Beyond mRNA, modifications in mitochondrial RNA, tRNA, and diverse noncoding RNAs have been increasingly implicated in disease pathogenesis [271, 474], and ongoing advances in DRS continue to extend its applicability to these clinically relevant RNA classes.

Despite its transformative potential, DRS faces several challenges that must be addressed before widespread clinical adoption. Sequencing throughput remains lower than that of short‑read Illumina platforms, and RNA input requirements are comparatively high. Limited RNA yields from clinical specimens, particularly liquid biopsies, further constrain applicability [479], although sample multiplexing strategies may partially mitigate this limitation [37, 334]. In addition, short RNA molecules are inefficiently captured by nanopores; however, optimization of acquisition parameters within ONT's MinKNOW software has been shown to substantially improve short‑RNA recovery [192]. DRS also exhibits systematic signal loss of approximately 15 nt at read termini, which can affect basecalling accuracy and transcript boundary resolution [480]. Although recent advances, including ONT's SQK‐RNA004 chemistry and updated flow cell designs, have improved sequencing throughput and enabled more reliable direct detection of RNA modifications such as m^6^A and Ψ, challenges related to RNA chemical stability and the lack of standardized regulatory and analytical frameworks continue to limit clinical implementation. These limitations are particularly relevant for the evaluation and quality control of mRNA vaccines and RNA‑based therapeutics [63].

Native RNA modification profiles offer distinct advantages over conventional expression‑based biomarkers for early disease detection and therapeutic response monitoring. Accumulating evidence indicates that epitranscriptomic alterations often precede measurable changes in transcript abundance, positioning RNA modifications as more sensitive and dynamic indicators of disease state [459]. Recent work in sepsis highlights this potential, demonstrating that nanopore DRS can directly sequence native RNA from minimally processed clinical samples, thereby enabling a viable path toward real‑time molecular diagnosis [381]. In parallel, by directly interrogating RNA structure, integrity, and modification authenticity, DRS complements established analytical techniques such as high‑performance liquid chromatography and mass spectrometry, providing a more comprehensive assessment of therapeutic RNA products. In summary, by delivering an unprecedented view of the transcriptome and epitranscriptome in their native state, nanopore DRS offers clear advantages for mRNA vaccine quality control, RNA biomarker discovery, and therapeutic RNA monitoring. Continued advances in AI‑powered basecalling, nanopore signal modeling, pore chemistry optimization, and standardized benchmarking frameworks are expected to further enhance accuracy, reproducibility, and interpretability. Together, these developments are likely to substantially expand the clinical reach of nanopore DRS, ultimately positioning it as an important technology for precision medicine applications.

## DESIGN, STANDARDIZATION, AND OPTIMIZATION OF DRS WORKFLOWS

With its capability to sequence individual RNA molecules at single‐nucleotide resolution, DRS has emerged as a useful platform for studying RNA modification dynamics [26]. In practical applications, the quality of experimental design, the degree of refinement in protocol optimization, and the level of standardization in operation collectively determine the reliability of the resulting data, the accuracy of modification detection, and the comparability of findings across different studies. In this chapter, we systematically summarize key aspects of DRS experimental design, library preparation strategies, multilayer quality control schemes, and recent methodological advances, with the aim of providing a comprehensive and practice‐oriented reference for selecting and implementing DRS‐based technical routes.

### Key considerations in experimental design: Refined sample selection and RNA pretreatment

In DRS studies, experimental design should be closely aligned with the underlying biological question and optimized at the levels of sample handling and RNA pretreatment. Different RNA classes, such as mRNAs, tRNAs, and circRNAs, exhibit pronounced differences in length distribution, structural complexity, modification profiles, and in vivo abundance, which influence the choice of library preparation schemes and sequencing strategies. Accordingly, explicit sample inclusion criteria and RNA quality control metrics need to be defined on the basis of the molecular properties of the target RNA species, together with tailored enrichment or depletion steps, to improve experimental success rates and robust data quality from the outset of the DRS workflow. Throughout this section, we use “DRS” only for workflows in which the native RNA strand is the sequencing template that passes through the nanopore. Across the six experimental strategies discussed here, reverse transcription and adapter engineering are used solely to stabilize RNA or improve capture, and the resulting reads always derive from native RNA molecules, consistent with this canonical definition of nanopore DRS.

### DRS library construction strategies for various RNA biotypes

Library construction is a pivotal step in DRS workflows, as it shapes RNA capture efficiency, full‐length read yield and the preservation of native RNA modifications. Different RNA biotypes vary substantially in length distribution, structural complexity, terminal modification patterns and abundance, necessitating tailored library preparation schemes to fully leverage DRS. In the following sections, we briefly summarize and compare library construction strategies for major RNA classes, including mRNAs, rRNAs, tRNAs, circRNAs, miRNAs, and other nonpoly(A) RNAs. Detailed experimental protocols for DRS library preparation of above RNA classes are provided both in the Note S1 and Github (https://zhangtianyuan666.github.io/DRS_doc).

#### mRNA library

**Figure 5:**
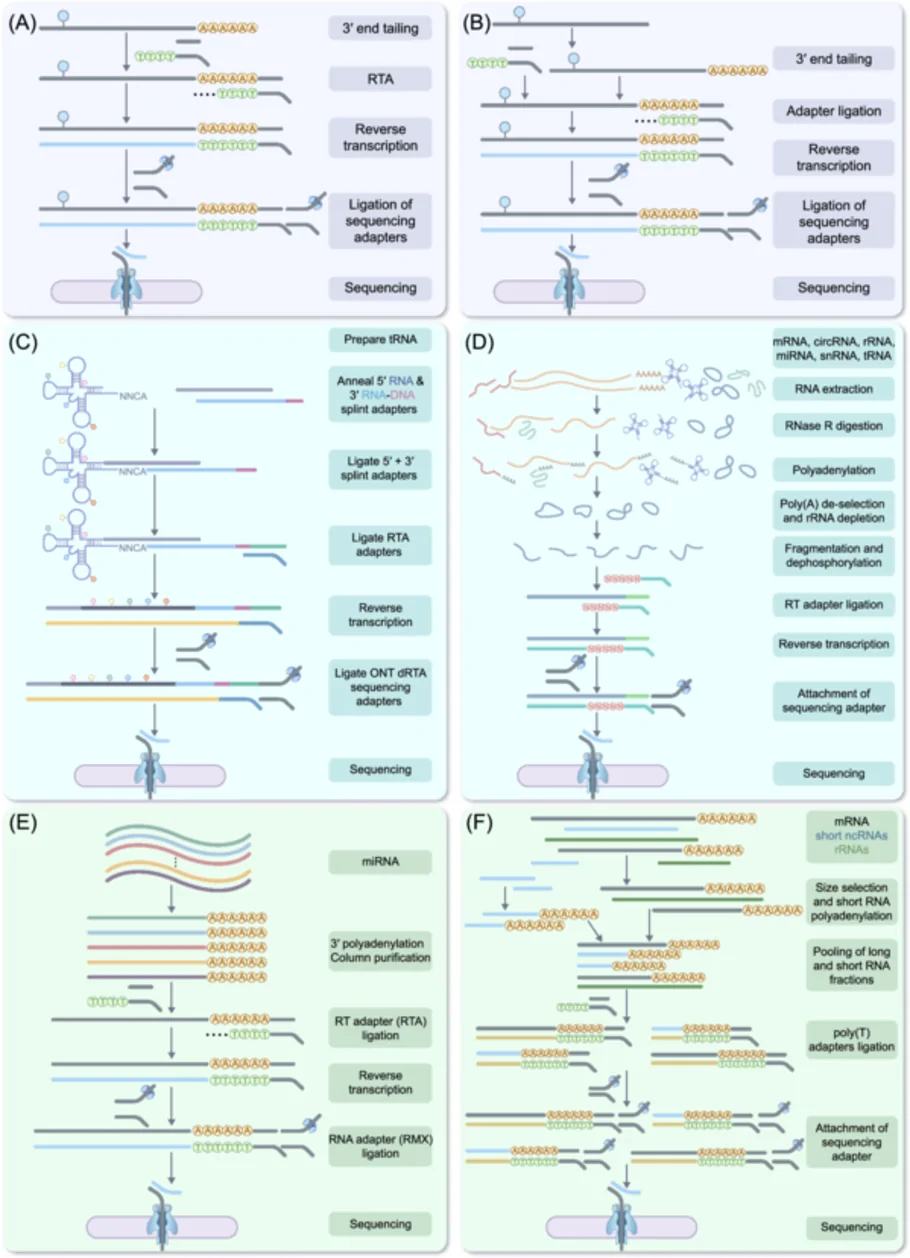
Schematic workflows for DRS of diverse RNA classes. (A) mRNA DRS workflow, illustrating 3′‑end tailing (if required), adapter ligation, reverse transcription primed at the 3′ terminus, ligation of sequencing adapters and nanopore sequencing. (B) rRNA workflow, including processing of primary rRNA transcripts, rRNA maturation, and direct RNA approaches, adapter ligation and sequencing. (C) tRNA workflow, showing annealing of 5′ and 3′ splint adapters to mature tRNAs, ligation of reverse‑transcription adapters, cDNA synthesis, ligation of ONT adapters and subsequent nanopore sequencing. (D) circRNA workflow, involving total RNA extraction, RNase R digestion to enrich circular molecules, polyadenylation and removal of residual linear RNAs, adapter ligation, reverse transcription, and loading of circRNA‐cDNA hybrids for sequencing. (E) miRNA workflow, in which small RNAs are enriched, subjected to 3′ polyadenylation, ligated to reverse‑transcription and RNA adapters, converted to RNA‐cDNA hybrids and sequenced. (F) Non‑poly(A) RNA workflow, depicting parallel processing of long and short nonpolyadenylated transcripts, size selection, pooling, ligation of poly(T) adapters, and construction of libraries compatible with DRS. cDNA, complementary DNA; circRNA, circular RNA; miRNA, microRNA; mRNA, messenger RNA; ONT, Oxford Nanopore Technologies; rRNA, ribosomal RNA; tRNA, transfer RNA.

mRNA library preparation is one of the most established and widely used workflows in DRS, with its central steps focusing on poly(A) enrichment and stabilization of transcript structure (Figure 5A). Overall, the protocol should be implemented under stringent quality control to ensure data quality. The workflow starts with total RNA extraction followed by comprehensive quality assessment. Samples should be free of visible discoloration or particulate matter, with an A260/280 ratio maintained at approximately 1.9−2.2 and an A260/230 ratio ≥2.0. A minimum total RNA yield of 2 μg is typically required to support robust downstream DRS library preparation. A minimum RIN value of 8.0 is recommended to ensure adequate preservation of full‐length transcripts [26, 481]. After passing quality control, poly(A)+ mRNAs are selectively enriched using oligo(dT)‐conjugated magnetic beads. The oligo(dT) oligonucleotides immobilized on the bead surface hybridize specifically to the 3′ poly(A) tails of mRNAs, allowing their separation from other RNA species. The enriched mRNAs are then ligated to a double‐stranded reverse transcription adapter (RTA) carrying a 3′ poly(T) overhang, which promotes specific annealing to the mRNA 3′ end and reduces nonspecific ligation events. Although RT is formally optional in DRS, it is recommended in most applications. Guided by the RTA, reverse transcriptase generates cDNA‐RNA hybrid molecules. This step enhances template stability during downstream handling and mitigates RNA degradation during sequencing. At the same time, partial unfolding of higher‐order mRNA structures decreases conformational resistance during nanopore translocation, thereby improving long‐read yield. Subsequently, sequencing adapters (RLA) harboring a motor protein are ligated to the prepared molecules. The motor drives the RNA through the nanopore at an approximately constant speed, resulting in more consistent ionic current signals, which is favorable for basecalling and modification detection. After bead‐based purification to remove unligated adapters and residual contaminants, the final library is mixed with sequencing buffer, loaded onto a flow cell, and sequenced on ONT platforms such as PromethION.

#### rRNA library

rRNAs are devoid of poly(A) tails and exhibit intricate higher‐order structures together with a high density of chemical modifications, features that collectively complicate library construction and often render them incompatible with conventional sequencing workflows (Figure 5B). Total RNA is first extracted and subjected to quality assessment, with a RIN ≥ 8.5 generally recommended to preserve native rRNA modification sites and secondary/tertiary structures, thereby reducing the accuracy drop caused by degradation in modification calling. As rRNA typically accounts for 80%−90% of total RNA, additional enrichment is not required. For nonpolyadenylated rRNAs, two representative approaches are commonly employed. One is in vitro polyadenylation, by adding poly(A) tails to the 3′ ends of rRNAs so that they can be processed with conventional mRNA library adapters. The other is to design customized RTAs complementary to rRNA 3′ termini, enabling specific recognition and ligation without polyadenylation [26, 325]. Given the highly structured nature of rRNA, improved structural unfolding is often required during library construction. In addition to generating cDNA‐RNA hybrids by RT, additives such as betaine can be included to improve the ability of reverse transcriptase to traverse structured regions and to increase the efficiency of full‐length rRNA synthesis.

Subsequent adapter ligation and purification steps are broadly similar to those of mRNA libraries, but sequencing parameters may need to be tuned to accommodate the long length of rRNAs (e.g., 28S rRNA spans several kilobases). Optimizing nanopore signal acquisition for long molecules helps to ensure complete read‐through. rRNA‐DRS libraries prepared in this way have been successfully used to profile diverse modifications in bacterial and eukaryotic rRNAs, such as m^7^G and Ψ in *E. coli* rRNA, and to characterize dynamic changes in rRNA modification patterns under stress conditions [325, 329].

#### tRNA library

tRNAs are short (approximately 70−90 nt), highly structured RNAs with dense stem‐loop elements and an unusually rich modification landscape, without a polyA tail (on average ~13 modifications per molecule). These features pose specific challenges for DRS library preparation, particularly in short‐fragment enrichment, structural unfolding, and adapter design (Figure 5C) [26]. In practice, relatively large input amounts (e.g., ≥ 10 μg total RNA) are often required to compensate for multistep losses, and RNA integrity is usually kept at RIN ≥ 8.5 to preserve tRNA structure and modification patterns [192].

Short RNA fragments can be enriched from total RNA using silica‐based purification systems that preferentially retain RNAs up to about 200 nt, thereby generating a fraction enriched in tRNAs [36]. The tRNA‐enriched fraction is then deacylated, for example by incubation in 0.1M Tris‐HCl (pH 9.0) at 37°C for 30 min, to remove aminoacyl groups from the 3′ ends that would otherwise interfere with adapter ligation. After neutralization with an acidic buffer and cleanup, deacylation efficiencies of ≥95% are typically achieved [192]. Exploiting the conserved NCCA motif at tRNA 3′ termini, a double‐stranded RNA adapter with defined overhangs can be designed, whereby the 3′ end carries an approximately 10‐nt poly(A) overhang to provide a binding site for subsequent RTA, whereas the 3′ end presents a UGGN overhang complementary to the NCCA sequence. Following the design, this adapter is ligated to the tRNAs. Extending the ligation reaction to roughly 16 h and supplementing the mixture with an RNA helicase (e.g., Rha) to relax local stem‐loop structures increases the ligation efficiency from about 60% to over 85% [36]. The resulting RNA molecules are then ligated to a double‐stranded RTA carrying a 3′ poly(T) overhang. RT is then carried out to generate cDNA‐RNA hybrids; osmolytes such as high‐concentration betaine are often added to facilitate traversal of structured regions by the reverse transcriptase, thereby improving the success of full‐length synthesis and increasing the proportion of full‐length reads by approximately 40% [192, 482, 483].

Subsequent ligation of motor‐containing sequencing adapters and bead‐based purification largely follow standard DRS workflows. However, run and basecalling settings must be adapted to the short length of tRNAs; for instance, adapter‐detection thresholds in MinKNOW may need adjustment to avoid misclassifying genuine tRNA reads as adapter dimers or contaminants. With appropriate optimization, the effective output of tRNA libraries can become comparable to mRNA libraries [36]. Such workflows have been successfully applied to tRNA modification profiling in yeast, *E. coli*, and human cells; for example, Nano‐tRNAseq uses a related strategy to detect loss of the Ψ55 modification in Pus4‐deficient strains [484].

#### circRNA library

circRNA lack free 5′ and 3′ ends, occur at relatively low abundance, and are easily masked by linear transcripts. Consequently, DRS library preparation for circRNAs relies on specific enrichment and adapter ligation strategies tailored to their circular topology (Figure 5D). Total RNA is first extracted and quality‐checked, with an input amount of ≥5μg generally recommended to ensure adequate capture of low‐abundance circRNAs and overall RNA integrity.

To deplete linear RNA quality RNA extraction and stringents, RNase R is commonly employed, taking advantage of its selective degradation of linear transcripts from their 3′ ends, while circRNAs, lacking free termini, are largely resistant and thus become enriched in the remaining fraction [485, 486, 487]. Following initial RNase R treatment, a secondary depletion step is recommended to eliminate any remaining linear RNAs, improving circRNA enrichment purity. The purified circRNAs are then subjected to controlled fragmentation to generate linear molecules of optimal length for sequencing. These linearized fragments possess accessible ends. A double‐stranded RNA adapter is ligated to the 3′ end of each fragment using T4 RNA ligase. Using the ligated adapter as a primer‐binding site, standard RT is performed to generate a cDNA‐RNA hybrid for each fragment, thereby converting the circular topology into a linear format compatible with sequencing.

Subsequent ligation of sequencing adapters, purification, and nanopore sequencing follow standard DRS protocols. However, basecalling and mapping require adjustments to accurately identify back‑splice junctions from circular templates. This workflow has been effectively used for profiling m^6^A modifications on plant circRNAs, demonstrating the utility of DRS for studying epitranscriptomic modifications in circular RNAs [485].

#### miRNA library

miRNAs are the shortest known non‐coding RNAs (approximately 18−22 nt) and occur at very low abundance, which creates two major obstacles for library construction because basecallers typically discard reads <50 nt, leading to substantial loss of miRNA signals, and the low copy number of miRNAs requires highly efficient enrichment to achieve adequate depth (Figure 5E) [488]. The workflow therefore begins with high‐quality RNA extraction and stringent QC, requiring RNA purity of A260/280 1.9−2.2 and A260/230 ≥ 2.0, a total input of ≥5 μg, and assessment of miRNA size distribution to exclude heavily degraded samples. A two‐step enrichment strategy is then applied. First, RNAs ≤200 nt are selected to deplete long transcripts and enrich the small‐RNA fraction. Second, biotinylated probes complementary to conserved regions of target miRNA families are used for hybridization, followed by streptavidin‐bead capture, which can increase the miRNA fraction from <0.1% in small‐RNA fraction to >50% in the enriched pool. To accommodate the very short inserts, customized single‐end adapters are employed, with the 5′ end carrying a barcode for multiplexing, and the 3′ end containing a short sequence complementary to the miRNA 3′ terminus, thereby reducing adapter dimer formation and nonspecific ligation. Ligation reactions are typically extended to 12−16 h and supplemented with ligase enhancers, yielding ligation efficiencies above 70%. A critical step is the reconfiguration of basecaller thresholds for adapter detection and minimal read length, lowering the retention cutoff to around 15 nt to prevent miRNA reads from being discarded as artifacts. Optional RT to generate cDNA‐RNA hybrids can be introduced, particularly for degraded or clinical samples, to stabilize miRNA‐derived molecules. After ligation of motor‐containing sequencing adapters and bead cleanup, libraries are sequenced on MinION/GridION/PromethION instruments. Downstream, miRNA‐oriented mapping and quantification pipelines are used, taking into account the short length and high sequence similarity of miRNAs and, where appropriate, incorporating secondary‐structure information to better distinguish homologous family members.

#### Non‐poly(A) RNA library

Non‐poly(A) RNAs are transcripts that lack a 3′ poly(A) tail, encompassing lncRNAs, snRNAs, snoRNAs, histone mRNAs, subsets of viral RNAs, and abundant structural RNAs such as rRNAs and tRNAs. Library preparation for this RNA class must be independent of poly(A)‐based capture to enable efficient enrichment and full‐length sequencing of the intended targets. Guided by non‐poly(A)‐oriented protocols such as NERD‐seq (Figure 5F) [194], the workflow is optimized for non‐poly(A) transcripts. Total RNA is first extracted and quality‐checked, with an RIN ≥ 8.0 and an input amount ≥3 μg. rRNAs are then depleted using biotinylated probes targeting conserved regions, followed by removal of probe‐rRNA complexes with streptavidin‐coated magnetic beads, typically achieving ≥95%type. For mRNA libraries rRNA depletion while minimizing mechanical fragmentation of nonpoly(A) species. Adapter ligation is carried out with a generic single‐stranded RNA adapter. The adapter bears a 5′ phosphate group, allowing RNA ligase‐mediated covalent joining to 3′‐hydroxyl termini of non‐poly(A) RNAs without the need for a pre‐existing poly(A) tail. The RNA products generated in the previous step are subsequently joined to a double‐stranded RTA adapter bearing a 3′ poly(T) overhang. RT is then carried out to generate cDNA‐RNA hybrids. Subsequent ligation of sequencing adapters and purification steps follow standard DRS procedures. This strategy is applicable to bacterial transcripts and to viral RNA that lack poly(A) tails [301].

### Post processing and sequencing optimization of library preparation

A standardized and reliable DRS workflow requires fine‐grained optimization and multistep quality control across postlibrary processing, sequencing parameter tuning, and data‐analysis calibration, under a unified end‐to‐end QC framework. Library purification is a key optimization step. For conventional mRNA and rRNA libraries, a one‐step magnetic‐bead cleanup is employed, in which the first round removes unligated adapters, small‐molecule contaminants, and free nucleotides [36]. For specialized libraries such as tRNA and circRNA, dedicated purification strategies are required, whereas circRNA libraries require an additional step to eliminate residual linear RNAs remaining after RNase R digestion. The sequencing reaction mix is prepared strictly according to the manufacturer's instructions, adjusting the effective RNA library concentration to 100−200 pM to ensure optimal nanopore occupancy, and flow cell pore activity is checked prior to loading to minimize pore blockage and yield loss. Sequencing parameters are then tuned according to RNA type. For mRNA libraries, a minimum mean read length threshold of ≥200 nt is applied; For tRNA libraries, dedicated basecalling models or modes tailored for short reads may be required to achieve accurate basecalling; for rRNA libraries; For rRNA libraries, higher‐order rRNA structures need to be disrupted during RT to ensure efficient copying of long rRNA molecules [9, 36]. The adoption of SQK‐RNA004 chemistry markedly increases throughput and accuracy, as compared with earlier kits, SQK‐RNA004 improves per‑read accuracy and reduces mismatch and insertion errors, and ONT internal benchmarking further reports a ~2.5‑fold increase in throughput and an increase in median read accuracy from ~93% to ~98.7% when using optimized basecalling models, thereby providing higher‑quality raw signals for RNA modification detection [62, 489].

## DRS DATA ANALYSIS

**Figure 6:**
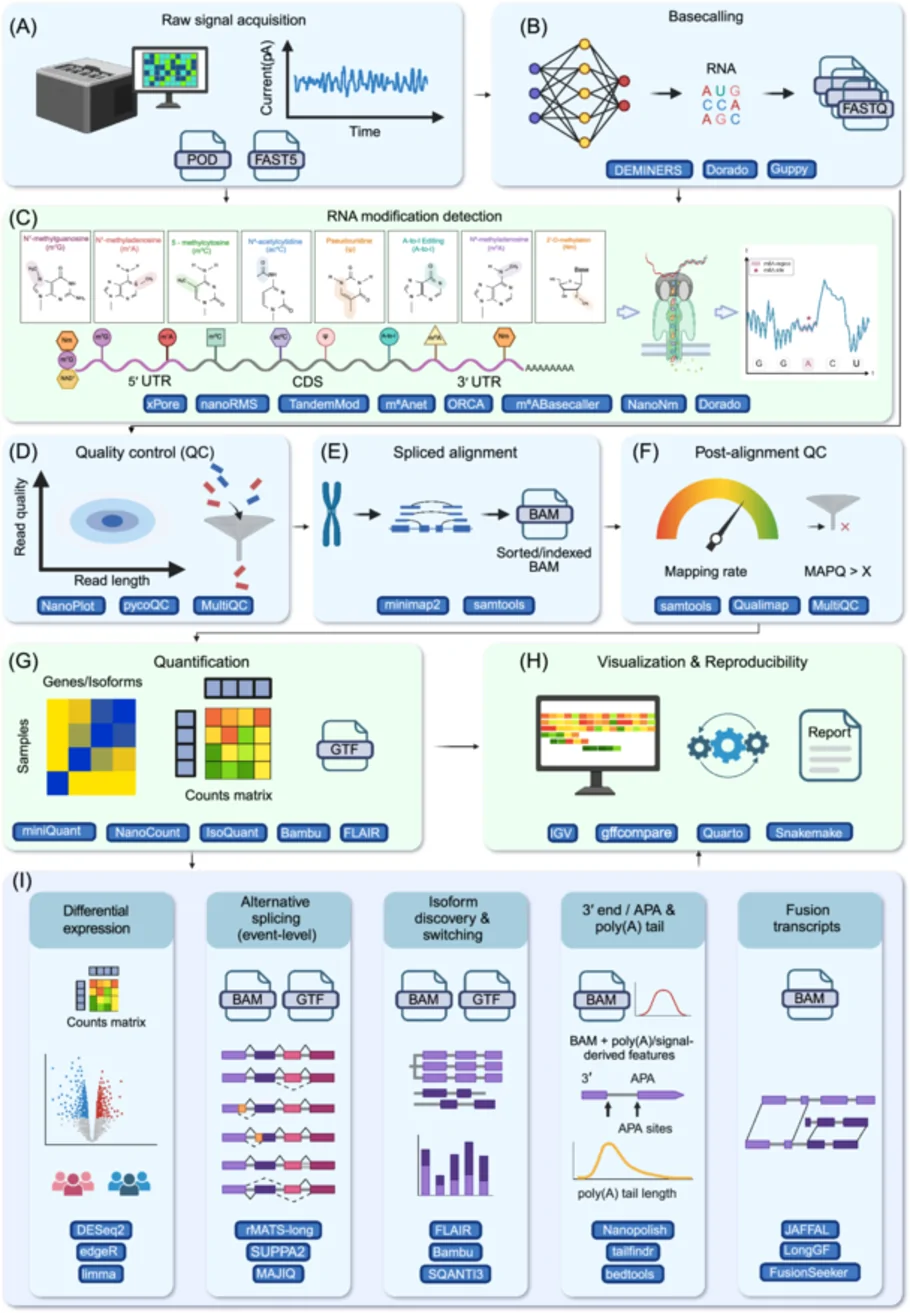
End‐to‐end computational workflow for DRS data analysis and downstream applications. Schematic representation of a typical DRS bioinformatics pipeline, illustrating the progression from raw data to biological interpretation, with corresponding bioinformatics tools for each analytical phase highlighted in blue boxes. (A) Acquisition and storage of raw ionic current signals in native nanopore sequencing file formats (POD/FAST5). (B) Basecalling transforms raw ionic current signals into nucleotide sequences formatted in standard FASTQ. (C) Identification of RNA modifications from signal‐level characteristics spanning essential transcript regions (5′ UTR, CDS, and 3′ UTR), encompassing prevalent epigenetic markers and editing occurrences (e.g., m^6^6A, m^5^C, Ψ, ac^4^C, m^1^A, m^7^G, A‐to‐I, Nm). (D) Pre‐alignment quality control to evaluate fundamental sequencing variables such as read length and read quality. (E) Spliced alignment of DRS reads to the reference genome/transcriptome, resulting in aligned reads processed into sorted and indexed BAM files. (F) Postalignment quality control to summarize genomic mapping parameters, including overall mapping rate and distributions of MAPQ scores. (G) Transcript and gene‐level quantification informed by standard GTF, producing a gene/isoform counts matrix for subsplice junctions, estimationsequent statistical analysis. (H) Data visualization and reproducible scientific reporting, encompassing genome‐browser‐based examination of transcriptome characteristics and organized workflow management for analytical reproducibility. (I) Diverse downstream biological analyses facilitated by long‐read DRS data encompass transcript differential expression analysis, event‐level alternative splicing analysis, de novo isoform discovery, isoform switching analysis, 3′ end usage/alternative polyadenylation analysis, poly(A) tail length estimation, and fusion transcript detection from full‐length RNA reads. ac^4^C, N4‐acetylcytidine; A‐to‐I, adenosine‐to‐inosine RNA editing; BAM, Binary Alignment Map; CDS, coding sequence; GTF, Gene Transfer Format; m^1^A, 1‐methyladenosine; m^5^C, 5‐methylcytosine; m^6^A, N6‐methyladenosine; m^7^G, 7‐methylguanosine; MAPQ, mapping quality; Nm, 2′‐O‐methylation; UTR, untranslated region; Ψ, pseudouridine. Created in BioRender. Li, Y. (2026) https://BioRender.com/0zmrexv.

Nanopore DRS generates raw ionic current signals that require multiple layers of computational processing before biological interpretation can be achieved (Figure 6A). Accordingly, a typical DRS data analysis workflow can be broadly organized into three major stages, namely (i) signal‐level processing and basecalling, in which raw ionic current traces are converted into nucleotide sequences; (ii) RNA modification detection, leveraging signal‐ or sequence‐level features to identify chemical modifications along RNA molecules; (iii) sequence alignment, transcript‐level quantification, and extraction of other biologically relevant RNA features, such as poly(A) tail lengths, splicing patterns, and additional posttranscriptional regulatory signatures.

The analysis begins with basecalling, where raw ionic current signals are converted into nucleotide sequences (Figure 6B). Basecallers utilize a variety of architectures, including *Hidden Markov Model* (HMM) [490, 491], convolutional neural networks (CNNs) [492], recurrent neural networks (RNNs) [493], attention mechanisms [494], and transformer models [495], to interpret current signals [496, 497].

Following basecalling, nanopore DRS enables a wide spectrum of RNA‐centric analyses, with RNA modification profiling representing one of its most important capabilities (Figure 6C). Unlike short‐read sequencing approaches that rely on RT or chemical conversion, nanopore sequencing directly measures ionic current disruptions as native RNA molecules pass through the pore [56, 388, 498]. Chemical modifications on nucleotides alter the local current signal and basecalling behavior, generating characteristic deviations that can be computationally modeled to infer modified bases at single‐molecule resolution [177, 183, 188, 499]. Recent advances in modification‐aware basecalling frameworks now support transcriptome‐wide detection of several prevalent modifications, including m^6^A, A‐to‐I, Ψ, and m^5^C, while preserving full‐length transcript context [372, 500].

In addition, systematic read‐level quality control (QC) is performed to assess sequencing performance and curate the dataset prior to downstream analysis. QC procedures typically include evaluation of read length distributions and removal of low‐quality or excessively short reads (Figure 6D). NanoPlot [501] provides comprehensive visualization and summary statistics of read length distributions, quality scores, throughput, and channel activity from FASTQ or sequencing summary files. Fastplong [502] provides an integrated framework that combines QC reporting, flexible read filtering, adaptor trimming, and poly(A) tail removal, features particularly relevant for DRS and full‐length transcriptome analyses. In practice, visualization tools and filtering utilities are often used together to ensure standardized, reproducible, and high‐quality ONT data processing.

Beyond RNA modifications, Nanopore DRS also enables downstream transcript quantification and structural characterization. Full‐length reads are mapped to their genomic or transcriptome origin using long‐read‐aware spliced aligners (Figure 6E). Optionally, postalignment quality control can be performed to assess mapping accuracy, splice junction support, read completeness, and coverage uniformity, thereby minimizing artifacts and false‐positive transcript models (Figure 6F). Based on read alignments, transcript reconstruction frameworks define full‐length transcript isoforms, refine transcript boundaries, and update gene annotations [209], followed by isoform‐ and gene‐level expression quantification to enable differential expression analysis across conditions (Figure 6G) [86]. Representative alignment patterns and transcript architectures can be further visualized in genome browsers (Figure 6H). Beyond expression profiling, nanopore DRS supports comprehensive characterization of RNA processing and structural diversity, including detection of AS patterns, identification of novel splice junctions, estimation of poly(A) tail length and APA sites, mapping of transcription starts and termination sites, and discovery of fusion transcripts and noncoding RNAs (Figure 6I). Together, these capabilities provide a unified, transcript‐resolved view of RNA processing and posttranscriptional regulation. Importantly, because nanopore DRS sequences full‐length native RNA molecules at single‐molecule resolution, these diverse features can be measured concurrently on the same RNA strand, enabling direct investigation of how RNA modifications are coordinated with splicing patterns [503], poly(A) tail dynamics, and transcript stability [60], and thereby offering an integrated perspective on post‐transcriptional gene regulation that is not achievable with fragmented or ensemble‐based sequencing approaches.

### Basecalling

Basecalling is the computational process that translates raw nanopore ionic current signals into nucleotide sequences by inferring base sequence underlying the measured electrical signal. A variety of basecalling tools are available for DRS [334, 504, 505, 506]. Guppy is an earlier ONT basecaller, developed in C++ as a closed‐source replacement for Albacore, and in the context of DRS it supports only the SQK‐RNA002 chemistry, making it increasingly outdated as new chemistries emerge. Dorado is ONT's current flagship high‑performance basecaller, designed to convert raw nanopore electrical signals into DNA or RNA sequences using optimized deep‑learning architectures. Compared to Guppy, Dorado offers substantial improvements in basecalling accuracy and throughput, supports multiple flow cells and chemistries including SQK‐RNA002 and SQK‐RNA004, and features a modular design that enables advanced functionalities such as modified basecalling and duplex calling. Unlike Guppy and Dorado, which are primarily optimized for inference, Bonito offers greater flexibility for users who wish to retrain or fine‑tune basecalling models on custom data sets, though this comes at the cost of requiring deeper computational expertise and more extensive GPU resources.

Among open‑source community tools, RODAN [504] was the first to introduce an EfficientNet‑style pure convolutional architecture for RNA basecalling. DEMINERS [334] is a comprehensive RNA‐omics toolkit that extends beyond basecalling to include barcode demultiplexing, direct pathogen assembly, and clinical metagenomic analysis. GCRTcall [505] adopts a fundamentally different strategy by applying the Conformer architecture from speech recognition to nanopore RNA signal processing. Coral [506] represents one of the most recent advances in RNA basecalling and implements an extreme form of dual‑context “signal‐sequence” modeling.

In summary, the selection of a nanopore RNA basecalling tool should be guided by specific application needs. Dorado (for SQK‐RNA004) or Guppy (for SQK‐RNA002) is appropriate for plug‑and‑play usage and maximum compatibility. For early SQK‐RNA002 data sets with sufficient computational resources, Coral currently provides the highest accuracy, while DEMINERS is optimal when integrated basecalling and barcode demultiplexing are required. With the increasing adoption of SQK‐RNA004 chemistry and improved accessibility of GPU resources, nanopore RNA basecalling has effectively entered the “99% accuracy era.” For users prioritizing processing speed, stability, and ecosystem compatibility, Dorado remains the preferred choice. In contrast, most open‑source models are implemented in Python and lack industrial‑grade deployment optimization, resulting in comparatively lower decoding throughput.

### RNA modifications detection tools

DRS enables the direct study of RNA chemical modifications by reading individual RNA molecules in real time. As an RNA strand ranslocates through a nanopore, chemical modifications create distinct perturbations in the ionic current signal [460]. By decoding these signal changes, researchers can identify modification sites at the single‐molecule level. Over the past few years, a growing suite of computational tools has been developed to detect and map these RNA modifications from DRS data [334, 504, 505, 506]. An overview of tools for detecting RNA modifications based on DRS is summarized in Table S3. These methods can be broadly classified into three categories based on their underlying principles, including statistical comparison of nanopore sequence or signal features, machine‑learning models trained on signal features, and modification‐aware basecalling approaches.

Statistical‐based approaches represent the earliest and most conceptually straightforward class and can be further subdivided into two major categories. The first category comprises statistical, signal‐level comparison frameworks that treat RNA modifications as distributional shifts in the raw current signal. These methods typically operate after “resquiggling” (realigning signal events to reference positions) and then test whether current intensity or dwell‐time distributions differ between experimental conditions, such as modified versus unmodified samples or wild‑type versus knockout data sets. Early pipelines such as Tombo [507] established the basic workflow of signal alignment and per‐site hypothesis testing, while comparative frameworks such as Nanocompore [34] formalized differential modification detection by contrasting signal distributions across conditions using Kolmogorov‐Smirnov tests or bivariate Gaussian mixture models. More recent probabilistic approaches such as xPore [185] model per‐site signals as mixtures of modified and unmodified states via a Bayesian framework, enabling estimation of modification rates (stoichiometry) and differential modification across samples, often without requiring a perfectly “unmodified” control. Related single‐molecule quantification frameworks, exemplified by nanoRMS [186], extend distributional comparison to per‐read clustering, enabling stoichiometry estimates and heterogeneity analysis at the molecule level. The primary strength of statistical signal comparison methods lies in their discovery potential, as they are modification‐agnostic and do not require prior knowledge of modification signatures; however, they typically demand deep sequencing coverage, are sensitive to signal alignment accuracy, and cannot distinguish between different modification types that produce similar current perturbations.

The second category leverages basecalling error signatures as proxies for RNA modification. In this paradigm, modifications are inferred from systematic mismatches, indels, trace deviations, or quality‐score perturbations at specific sites. Tools such as DiffErr [52] and DRUMMER [387] exemplify differential error‐based detection for m^6^A, typically by comparing wild‐type to writer knockout or knockdown conditions and identifying positions where error profiles shift reproducibly. DRUMMER includes a specific m^6^A mode that reports the distance to the nearest AC dinucleotide and the 5‑nt sequence motif centered on that motif (NNACN). EpiNano [56] extended this logic by building supervised classifiers on engineered error features derived from synthetic constructs, demonstrating that basecaller errors can carry reproducible modification information even without explicit signal modeling. EpiNano is specifically trained and applied within RRACH motifs (the known m^6^A consensus sequence), as its analysis and predictions are restricted to this motif context. Similarly, NanoPsiPy [508] exploits the unique U‐to‐C mismatch pattern characteristic of Ψ in DRS data. ELIGOS [173] uses statistical models of systematic basecalling errors to detect various RNA modifications at single‐base resolution without requiring machine learning. Error‐driven strategies are computationally efficient and can perform well for modifications with strong, consistent basecaller error footprints; their main limitations are tight coupling to specific basecaller versions and chemistry platforms, as well as reduced robustness for modifications that do not produce distinct error signatures.

Machine learning‐based approaches constitute the largest group and infer RNA modifications directly from nanopore signal features. These methods employ supervised or weakly supervised machine learning approaches that operate on engineered features derived from either raw current signals, basecalling traces, alignment context, or combinations thereof. These models use either machine learning models such as support vector machines (SVM), random forests, gradient boosting, or deep learning architectures such as BiLSTMs, ResNets, attentions, and transformers to map feature vectors around candidate sites to modification probabilities. For m^6^A, tools such as MINES [176], which employs a random forest classifier, and nanom^6^A [172], which utilizes an XGBoost model, combine current‐derived features with motif or k‐mer constraints and train classifiers using sites supported by orthogonal assays or synthetic standards. m^6^Anet [175] employs a deep learning framework based on multiple‐instance learning (MIL), enabling site‐level m^6^A detection and stoichiometry estimation without requiring read‐level labels or matched knockout controls. RedNano [509] integrates raw signal features with base‐calling error profiles through a residual neural network, achieving strong generalization across phylogenetically distant species. Xron [58] incorporates a convolutional recurrent neural network combined with HMM to perform methylation‐aware basecalling, allowing simultaneous sequence decoding and m^6^A identification from the same signal stream. Models like pum^6^a [405] and SingleMod [179] use techniques such as positive‐unlabeled learning and attention mechanisms to deduce modification states from individual reads, even with incomplete training data.

For other marks, analogous feature‐engineered and learning‐based strategies have been extended beyond m^6^A to encompass a broader spectrum of epitranscriptomic modifications. For m^5^C, deep learning frameworks including CHEUI [182], TandemMod [177], and modCnet [188] combine raw signal features with neural architectures to enable transcriptome‐wide and single‐molecule resolution profiling. For Ψ, a diverse set of tools has emerged that leverage both signal distortions and base‐calling discrepancies. Methods such as NanoPsu [57], Penguin [187], and IndoC [510] use supervised or semi‐supervised machine learning on signal and alignment‐derived features. More recent deep learning models, NanoSPA [183] and NanoMUD [499], further enable high‐resolution and, in some cases, multi‐mark detection involving Ψ and related derivatives such as m^1^Ψ. For A‐to‐I RNA editing, signal‐aware deep learning approaches such as Dinopore [511] directly model ionic current traces using convolutional neural networks, while DENA [174] and related frameworks demonstrate that recurrent architectures trained on native RNA can capture editing‐associated signal perturbations without reliance on genome‐based SNP filtering. Complementary anomaly‐detection strategies, such as iForest [512], instead exploit systematic base‐calling deviations to distinguish C‐to‐U editing events from sequencing noise. Detection of Nm has similarly benefited from supervised models that integrate signal statistics and k‐mer context, including Nm‐Nano [513] and NanoNm [514], which apply ensemble machine learning to achieve single‐nucleotide resolution mapping. Specifically, NanoNm has validated its Nm detection method across multiple species and identified FBL‐associated or Nm‐deficient sites in both rRNA and mRNA. A key challenge in detecting these modifications is their low abundance relative to m^6^A [515], creating severe class imbalance in machine learning models. This often leads to increased false positives when optimizing for sensitivity, while stricter thresholds to improve precision risk missing true modification sites, making accurate detection inherently difficult.

Beyond single‐modification tools, a growing number of integrative systems aim to characterize multiple RNA modification types simultaneously from the same dataset. Frameworks such as TandemMod [177], modCnet [188], NanoRL [516], DirectRM [180], ORCA [503], and CircRM [517] employ multilabel deep learning or transfer learning strategies to profile diverse combinations of m^6^A, m^5^C, Ψ, m^1^A, hm^5^C, ac^4^C, and related marks across transcript classes. Among them, 6 tools including m^6^Anet, singleMod, TandemMod, epiNano, NanoSPA, and Dinopore were retrained for simultaneously detecting multiple types of RNA modifications from both SQK‐RNA002 and SQK‐RNA004 data [518]. These multi‐mark approaches offer the advantage of maximizing information extraction from limited sample material, but they also face greater computational complexity and require carefully curated training data with ground‐truth annotations for multiple modification types.

Another category comprises basecaller‐integrated frameworks that streamline RNA modification detection by embedding it directly into the sequence decoding process. Rather than treating modification calling as a separate downstream analysis step, these approaches infer per‐read modification probabilities during basecalling or immediately thereafter, typically by jointly modeling nucleotide sequence context and modification‐induced perturbations in the ionic current signal. Representative tools in this category include Dorado [500], Remora [519], m^6^Abasecaller [372], mAFiA [181], and IL‐AD [520], all of which employ neural network architectures trained to simultaneously decode canonical sequence information and modification signatures from raw or basecaller‐derived signal features. For example, m^6^ABasecaller incorporates m^6^A prediction directly into the basecalling workflow, enabling real‐time, single‐molecule detection without requiring matched control samples or post hoc signal processing. Similarly, Dorado, the current basecaller developed by ONT, provides pretrained models for multiple RNA modifications, allowing concurrent sequence reconstruction and multitype modification inference within a unified computational pipeline. A notable advantage of basecaller‐integrated frameworks is their streamlined, one‑step workflow and reduced need for downstream post‑processing; however, they offer less flexibility for users who wish to customize feature engineering or apply alternative model architectures to specific research questions.

In practice, for SQK‐RNA002 data, m^6^Anet is recommended for m^6^A detection due to its balanced precision‐recall and strong wild‐type versus knockout discrimination [162, 518]. For SQK‐RNA004 data, Dorado is the default performer across m^6^A, m^5^C, Ψ, and A‐to‐I, achieving an area under the receiver operating characteristic curve (AUROC) of 0.9. Retrained SingleMod, TandemMod, EpiNano, NanoSPA, and DinoPore can serve as alternative options for detecting these RNA modifications. Notably, while m^6^A remains the most accurately detected modification, non‐m^6^A tools in both chemistries require substantial improvement in precision‐recall balance and biological validity [518].

In summary, RNA modification detection tools for nanopore DRS have evolved from statistical signal comparison and error‐based inference to advanced machine learning and basecaller‐integrated frameworks. These approaches differ in their assumptions, data requirements, and resolution, ranging from modification‐agnostic differential analyses to single‐molecule, multimark profiling. While learning‐based and integrated basecalling models offer improved sensitivity and scalability, careful benchmarking, model selection, and validation remain essential to ensure robust and biologically meaningful interpretation.

### Training data sets and resources for building nanopore RNA modification detection models

The development of accurate computational models for RNA modification detection from nanopore DRS data critically depends on the availability of high‐quality training data sets with reliable ground truth labels. Because nanopore signals are influenced by both sequence context and chemical modifications, training data must be carefully designed to disentangle these factors and expose models to diverse k‐mer environments and stoichiometries.

One major source of training data comes from IVT RNA with site‐specific modifications. In this approach, synthetic RNA molecules are generated in which a defined fraction or all copies of a particular nucleotide at known positions carry a specific modification. By sequencing both modified and unmodified versions of the same RNA, researchers obtain paired data sets that differ only in chemical state, providing clean labels for supervised learning. Such data sets are particularly valuable for learning modification‐specific signal signatures and for calibrating models to estimate modification stoichiometry. Representative examples include the widely used Curlcake and ELIGOS synthetic RNA constructs [56], in which long designer sequences were computationally generated to contain all possible 5‐mer contexts while minimizing RNA secondary structure. These sequences were split into manageable fragments, transcribed in vitro, and selectively synthesized with modified nucleotides such as m^6^A incorporated during transcription. A second important class of IVT training substrates consists of systematically designed short templates targeting defined 5‐mer contexts [173]. In these libraries, synthetic DNA templates are engineered such that a central 5‐mer contains one or more occurrences of a specific base, which is replaced during transcription with a modified nucleotide (e.g., m^6^A, m^1^A, m^5^C, hm^5^C, f^5^C, Ψ, m^7^G, or inosine). Flanking sequences are designed to avoid the same base, ensuring that the modification signal can be attributed unambiguously to the targeted positions. However, IVT systems often lack the sequence diversity of native transcriptomes, which can limit model generalization if used alone. To address this limitation, Wu et al. constructed an in vitro epitranscriptome training resource (IVET) [177, 188] by performing IVT on a large rice cDNA library, generating thousands of transcripts that collectively span a broad spectrum of natural sequence contexts.

However, despite their value as controlled training resources, IVT‐based modification data sets also have important limitations. In many IVT designs, the canonical nucleotide triphosphate is completely replaced with a modified analog. As a result, every occurrence of that base in the transcript becomes modified, rather than only a subset of biologically selected positions. This produces transcripts with unrealistically high and uniform modification density, which differs substantially from native RNA.

Another complementary strategy uses native biological samples combined with orthogonal experimental assays to provide site‐level labels for model training and evaluation. In this framework, transcriptome‐wide maps generated by biochemical methods combining with short reads sequencing are used as reference annotations to supervise or benchmark nanopore‐based predictions. For example, miCLIP enables near‐single‐nucleotide mapping of m^6^A through antibody crosslink, induced mutation signatures [34], while m^6^A‐REF‐seq and related endoribonuclease‐based approaches identify m^6^A sites by exploiting methylation‐sensitive cleavage patterns [166]. For m^5^C, RNA bisulfite sequencing provides base‐resolution detection by selectively converting unmodified cytidines [182]. A number of well‑constructed databases such as RMBase v3.0 [521], RM2Target v2.0 [522], MeT‐DB v2.0 [523], REPIC [524], and PRMD [525], aggregating RNA modifications from diverse epitranscriptome sequencing data sets across multiple species, provide an abundant resource for training models on specific RNA modification types.

Nevertheless, these reference maps have important limitations that must be considered when used as ground truth. First, most orthogonal assays are population‐averaged and do not preserve single‐molecule information, whereas nanopore sequencing measures modification signals at the level of individual RNA molecules. As a result, discrepancies may arise between site‐level enrichment detected by biochemical methods and per‐read heterogeneity observed in nanopore data. Second, many assays rely on antibody enrichment or chemical reactivity, which can introduce biases related to antibody specificity, cross‐reactivity, or incomplete derivatization. For instance, miCLIP can preferentially detect high‐stoichiometry sites and may miss lowly modified or structurally occluded positions, while bisulfite conversion efficiency in RNA can be affected by secondary structure, leading to false negatives or incomplete conversion [526]. In addition, RNA modifications are often dynamic and context‐dependent, varying across cell types, developmental stages, or environmental conditions [527].

Despite these caveats, orthogonal assays remain an essential resource for nanopore model development. When carefully matched in biological context and combined with IVT‐based synthetic controls and genetic perturbation data sets, they provide critical external validation and help anchor computational predictions to experimentally supported modification sites. A practical strategy for robust model development is to combine multiple data sources, using IVT data sets to learn modification‐specific signal signatures and orthogonal assays to provide biological context and validation, thereby leveraging the complementary strengths of each approach while mitigating their individual limitations.

### Isoform identification and quantification

Nanopore DRS provides full‐length, single‐molecule reads that span entire transcripts, allowing direct observation of exon connectivity, AS sites, TSS, and PAS. Because sequencing occurs on native RNA rather than cDNA, DRS avoids RT and PCR biases and preserves transcript structure together with molecular features such as RNA modifications and poly(A) tails [528]. This makes DRS particularly powerful for isoform‐resolved transcriptome analysis, where accurate reconstruction of complete transcript structures is essential.

Several specialized computational tools have been developed to reconstruct and quantify isoforms from long‐read RNA data (Table S4). IsoQuant [90] uses an intron‐graph framework to model splice junction connectivity and performs well in balancing sensitivity and precision across diverse transcript structures. However, its performance may be affected when reads are highly truncated at both ends. StringTie2 [89], originally developed for short reads, includes a long‐read mode that builds splice graphs from aligned reads to assemble transcript models and estimate abundances. StringTie2 demonstrates high computational efficiency and strong sensitivity across data sets, though its performance can be affected by high sequencing depth or read truncation at both ends. Bambu [91] applies statistical modeling and adaptive thresholds to control false discovery while enabling novel isoform detection, making it particularly suitable when a reference annotation is available but incomplete. Bambu heavily relies on splice junctions for transcript discovery, which limits its ability to identify transcripts that differ only in alternative start or end sites, and its default exclusion of subset transcripts may lead to missed valid isoforms while biasing quantification toward nonsubset transcripts [91]. Additional graph‐ and clustering‐based approaches have also been proposed, including Freddie [529], which detects AS patterns through read clustering without relying on existing annotations, and ESPRESSO [530], which emphasizes accurate splice junction correction and isoform quantification from long‐read RNA‐seq data despite its substantial memory consumption.

Other frameworks emphasize correction and filtering of long‐read‐specific artifacts. FLAIR [88] integrates splice‐junction correction and can incorporate short‐read evidence to refine isoform boundaries. TALON [531] focuses on annotation consistency across replicates and is often used in multi‐sample studies to track known and novel transcripts. FLAME [532] models read truncation and transcript length biases, which is helpful for distinguishing true isoforms from partial reads. SQANTI3 [533] provides a comprehensive quality‐control and classification framework for long‐read transcript models, enabling systematic assessment of splice junction validity, structural novelty, and artifact enrichment relative to reference annotations. Recently developed tools such as Longcell [534] further extend isoform‐resolved analysis by integrating long‐read transcript structures with single‐cell resolution. Benchmarking studies using spike‐in controls and nanopore data sets show that graph‐based methods such as IsoQuant and StringTie2 generally achieve strong sensitivity for complex genes, while model‐based approaches like Bambu can better control false positives when discovering new isoforms [215, 535].

Using these tools, DRS data enable direct identification of diverse isoform features, including exon skipping, intron retention, alternative donor and acceptor sites, promoter switching, and alternative last exons. Because each read represents a single RNA molecule, isoform abundance can be directly estimated from read counts without fragment reconstruction, while isoform identity can be analyzed together with RNA modification profiles, poly(A) tail length, and transcriptional termination variability. This enables integrated investigation of transcript architecture, RNA processing, and posttranscriptional regulation at single‐molecule resolution [52, 86, 189, 328].

Despite these advantages, isoform reconstruction from DRS remains sensitive to long‐read‐specific technical factors. Residual basecalling errors, incomplete 5′ end coverage, and alignment ambiguity in repetitive regions can lead to false splice junctions or truncated isoforms [35, 81]. Increasing sequencing depth improves detection of low‐abundance transcripts but can also elevate false‐positive isoforms if filtering is not applied [84]. Therefore, practical analyses usually apply minimum junction support thresholds, transcript read support filters, and optional hybrid correction using srRNA‐seq data [91].

In a typical DRS isoform analysis workflow, raw POD5 signal files are first basecalled with neural network models such as Dorado from ONT. The resulting reads are aligned to a reference genome using splice‐aware long‐read aligners such as Minimap2, configured for native RNA. Aligned reads are then processed by long‐read transcriptome assemblers to collapse reads into isoform models and quantify expression. Subsequent filtering, annotation comparison, and differential isoform analyses produce a high‐confidence, isoform‐resolved view of the transcriptome. Benchmarking analysis revealed that IsoQuant consistently achieved the highest precision and sensitivity across most simulation scenarios, sequins data sets, and experimental data sets [215]. It performed particularly well in complex settings such as high isoform diversity and incomplete annotations, and it maintained stable performance across varying sequencing depths and error rates. Therefore, IsoQuant is recommended for accurate isoform detection in long‐read RNA sequencing data.

### Poly(A) and APA analysis

In most eukaryotes, the poly(A) tail is a non‑templated adenosine tract at the mRNA 3′ end that contributes to mRNA maturation and export and shapes cytoplasmic mRNA stability [385, 536]. Historically, transcriptome‑wide poly(A) profiling has relied on RT and PCR‑based methods, which can introduce amplification‑driven distortions and decouple tail features from the native RNA molecules being inferred [226]. Nanopore DRS addresses these limitations by sequencing native RNA molecules directly, enabling single‑molecule linkage between isoform identity and poly(A) tail information within the same read [35].

In ONT DRS, RNA molecules are physically translocated through the nanopore in the 3′‑to‑5′ direction, while basecalling software reports sequences in the 5′‑to‑3′ orientation, placing the poly(A) segment at a stereotyped location in the raw signal [35, 228]. Consequently, most poly(A) length estimators operate at the signal level, segmenting the low‑variance poly(A) current plateau, quantifying its duration in raw samples, and converting this value to nucleotides using a per‑read estimate of the translocation rate, typically expressed as samples per nucleotide [9, 35, 226, 228, 229, 492].

Several tools have become widely used for poly(A) localization and length estimation from DRS data, differing mainly in how they segment the signal and normalize for read‑specific kinetics. Nanopolish [491] implements a segmentation HMM and uses Viterbi decoding to infer region boundaries that include the poly(A) segment, estimating tail length from the inferred poly(A) duration and a read‑specific rate [35]. tailfindr provides an alignment‑free alternative that uses smoothed signal heuristics to locate the tail boundary and derives per‑read normalization from basecaller‑derived information [228]. Benchmarking against synthetic RNA standards demonstrated that tailfindr achieves high accuracy for poly(A) estimation, performing comparably to Dorado across various tail lengths [537]. However, its runtime is slower than that of Dorado and Nanopolish, and it requires basecalled FAST5 files, which adds preprocessing complexity and limits compatibility with newer sequencing workflows. BoostNano [537] employs a deep‑learning sequence‑labeling strategy to segment adapter‑ and tail‑associated regions directly from raw signal [492, 537]. BoostNano exhibits pronounced multimodal distributions and tends to overestimate very short poly(A) tails [537]. Dorado, ONT's official basecaller, offers built‑in poly(A) and poly(T) length estimation and records the estimate as a BAM tag. It identifies a primer anchor for the tail, uses the move table to delimit the tail interval in raw signal, and converts signal span into nucleotide length [537]. Recent benchmarking using synthetic RNA standards recommended Dorado as a preferred approach due to its fast runtime and low mean error [537]. For downstream comparison and visualization, TAILcaller [538] operates directly on Dorado BAM files, whereas NanoTail provides analysis utilities focused on Nanopolish outputs [229]. Benchmarking using synthetic RNA standards (Sequins) with known poly(A) tail lengths demonstrated that nanopolish, tailfindr, Dorado, and BoostNano all recover mean tail lengths within ~12% of the ground truth, although they differ in variance, computational efficiency, and stringency of read filtering [537].

Beyond tail length measurement, nanopore DRS also supports analysis of APA. Alignment of full‐length reads reveals transcript 3′ end heterogeneity, allowing identification of distinct PAS within the same gene. APALORD [539] provides an integrated pipeline that quantifies poly(A) sites usage and performs differential APA analysis between conditions, enabling proximal‐distal switching to be assessed within a unified framework. LAPA [540] similarly clusters transcript termini into PAS peaks and estimates site usage, offering a flexible PAS‑calling approach applicable to long‑read data sets, including DRS, although differential testing is typically conducted downstream according to study‑specific designs.

In viral and other compact genomes, end‑site discovery tools are often used to define TSS and cleavage and polyadenylation sites (CPAS) catalogs that serve as proxies for PAS in APA‑like quantification. NAGATA [209] identifies CPAS by clustering enriched DRS 3′ ends while applying filters to reduce artifactual termini, whereas LoRTIA [541] detects statistically enriched transcript end sites from long‑read alignments using end‑enrichment signals and alignment features such as soft clipping. These approaches are frequently employed to reconstruct complex termination landscapes and can be readily extended to comparative APA analyses by quantifying reads assigned to alternative end‑site clusters across samples.

Most DRS studies still infer APA indirectly through isoform reconstruction followed by boundary standardization. DRS‑capable transcriptome analysis tools (IsoQuant [90], FLAIR [88], FLAMES [383], Bambu [91], TALON [531]) generate quantified transcript isoforms whose 3′ ends can be clustered on a per‑gene basis into PAS and compared across conditions. IsoTools [542] and IsoTools2 [543] further incorporate gene‑wise peak calling of 3′ ends to define candidate PAS within a long‑read transcriptome analysis framework, producing PAS catalogs that can be quantified across samples, although differential APA testing is typically performed downstream.

In addition to 3′ end analysis, transcript boundary definition at the 5′ end is conceptually related but technically more challenging in DRS data. Nanopore DRS enables observation of native RNA molecules but introduces systematic 5′ truncation due to incomplete capture of transcript termini during sequencing, which complicates TSS detection. NAGATA [209] and LoRTIA [544] identify TSS by analyzing the genomic distribution of read start positions in aligned DRS reads. Other approaches, such as TranscriptomeReconstructoR [545] and Telos [546], refine transcript boundaries using existing transcript annotations or external data sets, including cap‐associated short‐read data, rather than performing de novo TSS discovery from DRS reads.

Importantly, poly(A) tail length and APA analyses can be integrated with other DRS‐derived features at the single‐molecule level. For example, tail length distributions can be examined in the context of specific isoforms, RNA modification status, or splicing patterns, enabling investigation of coordinated regulatory mechanisms that link RNA processing events. This integrative capability represents a major conceptual advance over traditional poly(A) assays, which typically measure tail length independently of transcript identity.

Nevertheless, poly(A) tail estimation and APA analysis based on nanopore DRS remain sensitive to several technical factors, including signal noise, variable RNA translocation speed through the pore, basecalling model assumptions, and RNA degradation [109, 226, 228, 481]. Very short or partially degraded poly(A) tails may be difficult to resolve accurately, and systematic differences can arise between analytical tools or sequencing chemistries. Dorado, integrated directly into ONT's basecalling workflow, is recommended for most applications due to its fast runtime, low mean error, and conservative filtering. For scenarios where accuracy across highly variable tail lengths is prioritized over speed, such as low‐throughput studies, tailfindr serves as a strong alternative. In addition, internal priming at A‐rich genomic regions can mimic genuine cleavage sites, while alignment artifacts near transcript ends may shift apparent polyadenylation positions, making cleavage site clustering and filtering essential [52]. Consequently, best practices for DRS‐based poly(A) and APA analysis include stringent quality control and filtering of low‐confidence reads, removal of internal priming artifacts, comparison across biological replicates, and emphasis on relative or distributional differences rather than absolute tail length estimates when interpreting biological trends.

### Advanced analysis and visualizations

**Figure 7:**
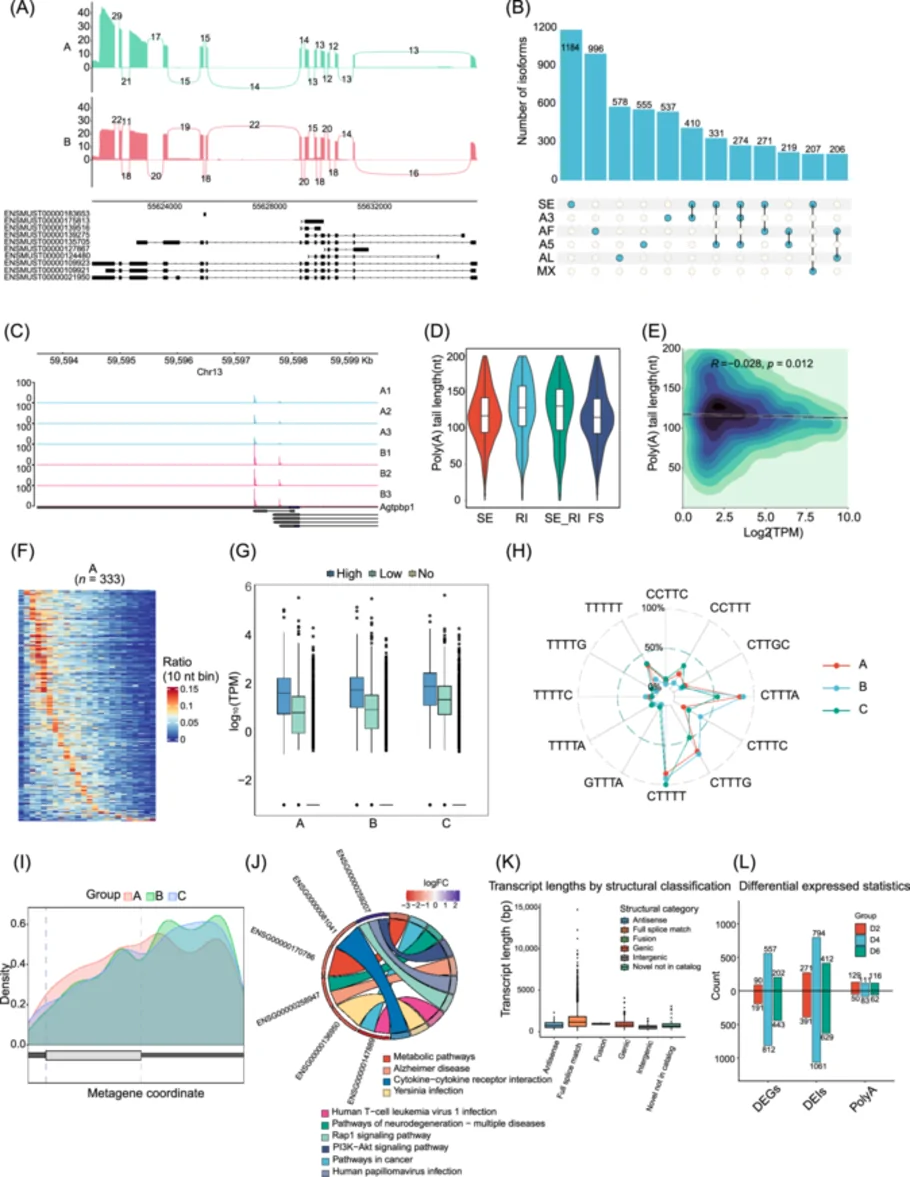
Examples of analytical visualizations commonly used in DRS studies. (A) Visualization of alternative splicing and transcript coverage using ggsashimi. Sashimi plot showing exon coverage and splice junction usage for a representative gene, with annotated transcript isoforms displayed below. (B) Combinatorial patterns of AS isoforms. UpSet plot showing the number of isoforms involving different alternative splicing types and their combinations (C) IGV visualization of APA at a representative gene revealed by DRS data. (D) Poly(A) tail length distributions associated with distinct AS event types. (E) Two‐dimensional density plot showing the association of transcript abundance and poly(A) tail length. The *x*‐axis represents transcript abundance as Log_2_(TPM), and the *y*‐axis shows poly(A) tail length (nt). Color intensity indicates the density of data points. (F) For each group (example shown for group A, *n* = 333 transcripts), the *x*‐axis represents poly(A) tail length, and each row corresponds to one transcript, with color indicating the proportion of reads for that transcript in each length bin. (G) Relationship between RNA methylation status and transcript expression. The *x*‐axis denotes sample groups (A–C), and the *y*‐axis shows transcript abundance (log_10_(TPM)). (H) Top enriched pseudouridine (Ψ)‐associated motifs across groups A, B, and C. Each axis corresponds to one motif, radial values indicate its percentage occurrence, and colored lines represent groups. (I) Modification profiles of Ψ site density across mRNA features in groups A, B, and C. The *x*‐axis represents the relative metagene coordinate from the 5′ UTR through the CDS to the 3′ UTR, and the *y*‐axis indicates Ψ site density. Curves correspond to different sample groups (J) Chord diagram linking selected genes to enriched pathways. The color scale (logFC) indicates the direction and magnitude of gene expression changes, while ribbons illustrate which genes contribute to each pathway. (K) Transcript length distributions across SQANTI3 structural transcript categories. The *x*‐axis indicates SQANTI3 structural categories, and the *y*‐axis shows transcript length. (L) Summary of differential expression and poly(A) changes across time points. Barplot showing the numbers of differentially expressed genes (DEGs), differentially edited isoforms (DEIs), and transcripts with differential poly(A) sites. A3, alternative 3′ splice site; A5, alternative 5′ splice site; AF, alternative first exon; AL, alternative last exon; APA, alternative polyadenylation; AS, alternative splicing; DEGs, differentially expressed genes; DEIs, differentially edited isoforms; FS, frameshift; IGV, Integrative Genomics Viewer; logFC, log2 fold change; MX, mutually exclusive exons; nt, nucleotide; RI, retained intron; SE, skipped exon; SE_RI: combined skipped‐exon/retained‐intron events; TPM, transcripts per million; Ψ, pseudouridine.

Existing DRS data analysis tools are often functionally fragmented and tend to focus on a single analysis layer, which creates a practical barrier for users without a strong bioinformatics background. Based on published DRS studies and common needs in real‐world projects, we summarized a set of advanced but frequently used analysis and visualization patterns for DRS data (Figure 7), and we provide the corresponding visualization example and code (https://zhangtianyuan666.github.io/DRS_doc). These visualization templates cover multiple layers of DRS analysis, including AS, APA, poly(A) tail length, RNA modifications, transcript structural categories, functional enrichment, and differential expression. They are intended to facilitate rapid construction of customized DRS analysis workflows tailored to specific biological questions. At the AS level, our example generates sashimi plots that summarize exon coverage and splice junction usage of representative genes across conditions (Figure 7A). UpSet plots further quantify how many transcripts are affected by each splicing category and by their overlaps (Figure 7B), helping to pinpoint condition‐dependent and co‐occurring splicing patterns.

For APA and poly(A) analyses, the example exports IGV‐compatible tracks that display poly(A) read coverage at the 3′ends of APA genes together with their annotated isoforms (Figure 7C), enabling direct visual comparison of 3′‐end usage between samples. Poly(A) tail length can be profiled by splicing event type (Figure 7D), and examined either globally using a two‐dimensional density plot of transcript abundance (Log_2_(TPM)) vs*.* tail length (Figure 7E) or at per‐transcript resolution using 10‐nt binned heatmaps of tail‐length distributions (Figure 7F).

For RNA modification, the pipeline links modification calls to expression and sequence context. Using methylation as an example, transcripts are grouped into High‐, Low‐, and No‐ modification classes, and their expression distributions are compared across sample groups (Figure 7G) to assess how modification status relates to transcript abundance. Then, enriched sequence motifs and their group‐specific frequencies are summarized with radar plots (Figure 7H), whereas modification profiles show the density of Ψ sites along 5′ UTR, CDS, and 3′ UTR (Figure 7I), revealing both shared and condition‐specific patterns of Ψ localization along mRNA. At the functional and structural levels, this example provides several global overview visualizations. The chord diagram links differentially expressed genes to enriched pathways, with color encoding the direction and magnitude of expression changes (logFC) and ribbon width reflecting each gene's contribution to individual pathways (Figure 7J), thereby highlighting key gene‐pathway modules. Transcript length can be compared across SQANTI3 structural categories, which characterizes the complexity of transcript structures in the sample and their length distributions (Figure 7K). In addition, by summarizing differential statistics across time points, the barplot shows the numbers of differentially expressed genes (DEGs), differentially expressed isoforms (DEIs), and transcripts with differential poly(A) site usage (Figure 7L). Together, these example data sets and scripts lower the technical barrier for nonspecialists to perform in‐depth DRS analyses, accelerate the selection and combination of appropriate analysis strategies, and promote more standardized and reproducible interpretation of DRS data.

## DESIGNING, VALIDATING, AND BENCHMARKING DRS: AN INTEGRATED FRAMEWORK

### Experimental design and benchmarking strategies for DRS

DRS is transitioning from a niche methodology to a general‑purpose platform for transcriptomic and epitranscriptomic research, supported by rapid advances in experimental workflows, specialized computational algorithms, and curated databases that collectively enable more comprehensive, accurate, and scalable analyses of RNA biology [51, 120]. Accordingly, the adoption of standardized experimental and algorithmic frameworks is essential to achieve robust, reproducible, and comparable DRS algorithm development and benchmarking [547, 548, 549, 550]. Extensive benchmarking and methodological studies have been conducted and thoroughly discussed for cDNA‑based ONT and PacBio sequencing platforms [81, 83, 84, 215, 551]. In contrast, this section focuses on experimental design considerations and benchmarking strategies specifically relevant to the development and evaluation of DRS algorithms.

### External spike‑in controls as experimental ground truths in DRS

Biological data sets vary substantially in sequencing depth, library chemistry, and genetic background, making sensitivity and specificity estimates difficult to compare directly across studies [552]. This challenge is further compounded by the inherent complexity of the epitranscriptome, which encompasses diverse RNA modification types, strong sequence‑ and context‑dependent effects, and interactions among proximal modifications [241]. Consequently, spike‑in‑based controls and other well‑defined ground‑truth resources, which provide known sequences, concentrations, and modified or unmodified states, have become indispensable tools for method development, benchmarking, and comparative evaluation. These resources enable more stringent, reproducible, and comparable assessment of DRS approaches [241, 553, 554].

#### ERCC spike‐ins

The External RNA Controls Consortium (ERCC) created ERCC RNA spike‐in controls to reduce experimental variability and provide a standardized reference for calibrating and quality‑controlling RNA quantification across platforms [555, 556, 557, 558]. These controls comprise 92 synthetic, polyadenylated transcripts intended for incorporation into an RNA analysis experiment postsample isolation, to evaluate against established performance standards. The ERCC transcripts vary in length from 250 to 2000 nt, resembling native eukaryotic mRNAs, and are provided as mixtures with specified sequences, lengths, and input concentrations across multiple orders of magnitude. Owing to their well‐characterized and broad dynamic range of absolute input quantities, ERCCs are used as traceable rulers for assessing quantification sensitivity, accuracy, and detection limit analysis across microarrays and RNA‑seq [552, 557, 558, 559, 560, 561, 562].

However, ERCC spike‑ins consist of mono‑exonic, single‑isoform, unmodified transcripts and therefore primarily probe dynamic range, linearity, and limits of detection [553, 558]. Their lack of splicing and isoform diversity precludes evaluation of isoform‑level quantification and AS performance [553, 563]. Moreover, ERCCs do not recapitulate RNA secondary structure, degradation dynamics, or cellular RNA processing, making them poor surrogates for assessing degradation‑aware quantification or context‑dependent biases [481]. Consequently, while ERCCs are well suited for benchmarking error correction and dynamic range, they are inadequate for evaluating higher‑order features central to DRS, including TSS and TTS, isoform complexity, and RNA modifications, all of which represent key strengths and distinguishing advantages of DRS [564, 565].

#### SIRV spike‑ins

To capture transcriptional and posttranscriptional complexity, the Spike‐in RNA Variants (SIRV) controls offer tunable, transcript‑like RNAs with known ground truth that overcome several limitations of ERCC‑style controls [566, 567]. The SIRVs consist of 69 synthetic, polyadenylated artificial transcripts modeled on seven human reference genes (https://doi.org/10.1101/080747). These multi‐exonic and predefined isoform variants can be mixed at known concentrations, enabling controlled benchmarking of alternative splicing, alternative TSS and TES, overlapping genes, antisense transcripts, and poly(A) length analysis workflows [81, 215, 537, 561, 563, 566, 568, 569, 570, 571, 572, 573, 574, 575]. Furthermore, SIRVs from human genes further yield modification‑free but sequence‑matched transcriptomes that act as negative controls for RNA‑modification detection in DRS, including k‑mer resolved false‑positive rate estimation [576, 577].

Unlike ERCC controls, SIRVs spike‑ins can be generated from real cDNA clones to recapitulate authentic splice isoforms and exon‐intron structures [568], or from multi‑cell‑line cDNA pools to approximate the diversity of the human transcriptome [576]. Consequently, SIRVs serve as more faithful surrogates for endogenous RNAs while retaining exact sequence identity and input‑amount ground truth. Despite their advantages, SIRV spike‐ins carry several inherent limitations. First, because SIRVs are synthesized via in vitro transcription, they lack native RNA modifications. Second, SIRVs do not recapitulate endogenous RNA secondary structures, limiting their utility for assessing structure‐aware assessment. Third, the predefined isoform architecture of SIRVs does not capture the full diversity of RNA processing, such as noncanonical splicing, or intron retention observed in real transcriptomes. Therefore, while SIRVs are indispensable for isoform‐level validation, they should be complemented with other orthogonal controls and computational methods when assessing native RNA features in DRS workflows.

### Modification‑encoded IVT spike‑ins

By enabling direct interrogation of native RNA molecules and circumventing biases introduced by RT and amplification, DRS offers an unprecedented opportunity to map the spatial distribution and dynamics of RNA modifications, including m^6^A, m^5^C, Ψ, and others [9, 26, 34, 56, 58, 114, 118, 119, 120, 162, 179, 185, 186, 189, 192, 246, 388, 578, 579]. This capability has propelled epitranscriptomics into an era of full‑length transcript analysis with single‑molecule, single‑base resolution of RNA modifications. However, for DRS‑based modification detection, there remains no universally accepted gold standard that provides both single‑nucleotide resolution and accurate stoichiometry for most modification types [162, 241, 580].

To rigorously evaluate and compare the performance of DRS platforms and computational algorithms for RNA modification detection, the establishment of robust and reliable benchmarking standards is essential [577]. At present, the prevailing gold standard relies on synthetic spike‑in controls generated by IVT of RNAs containing site‑specific or motif‑defined modifications, introduced either through incorporation of modified nucleotides or via posttranscriptional enzymatic treatment [567, 581, 582, 583, 584, 585, 586, 587, 588, 589, 590, 591, 592, 593]. These synthetic constructs, each paired with an isogenic unmodified control, provide explicit ground truth and enable systematic assessment at both site‑level and read‑level resolution, which is essential for training and validating supervised machine‑learning models for RNA modification identification [181, 241, 576, 577, 594].

**Figure 8:**
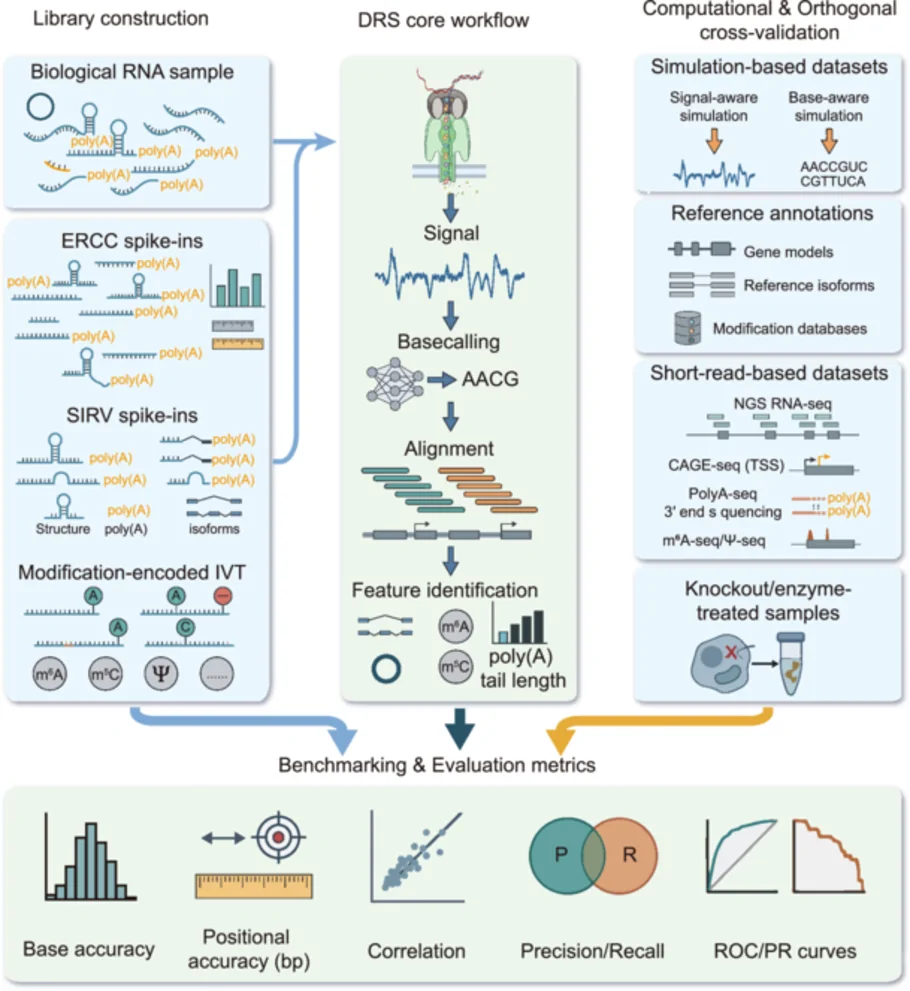
Experimental design and multi‐layer benchmarking framework for DRS. Synthetic spike‑in controls, including ERCC RNAs and customizable IVT RNAs provide defined ground truth for benchmarking quantification, isoform structure, transcript boundaries, and poly(A) tail length. Modification‐encoded IVT spike‑ins carrying site‑specific or motif‑defined RNA modifications enable supervised training and evaluation of modification‑calling algorithms. Simulation‑based data sets further complement experimental controls by enabling fully programmable, scalable benchmarking of sequence‑ and signal‑level features. Orthogonal biological resources, including curated genome annotations, epitranscriptomic databases, matched short‑read sequencing, and 5′/3′ end profiling assays, provide independent validation of transcript structure and modification calls. Genetic and enzymatic perturbations, such as writer/eraser knockouts or modification‑specific chemical treatments, offer causal validation of DRS‑derived features. Together, these resources support multilayer benchmarking from raw ionic current and basecalling accuracy to alignment, transcript reconstruction, quantification, and RNA modification detection, enabling robust, reproducible, and biologically grounded evaluation of DRS methods. ERCC, External RNA Controls Consortium; IVT, in vitro‐transcribed.

Nevertheless, these engineered, modification‑encoded spike‑ins often fail to recapitulate endogenous modification stoichiometry, may not fully capture the native sequence and structural contexts that shape modification‑specific signal patterns, and typically evaluate only a single modification type per construct [116, 145, 161, 163, 182, 554, 595]. Consequently, they do not reflect the inherent complexity of the epitranscriptome, in which multiple modifications frequently co‑occur on the same RNA molecule. Addressing these limitations through the development of more physiologically relevant and structurally complex spike‑in standards, together with community‑endorsed benchmark data sets, will be critical for standardizing the field and advancing toward truly quantitative epitranscriptomic profiling (Figure 8 and Table S5).

### Simulation‑based benchmark data sets for DRS

Beyond the experimental use of exogenous RNA spike‑in as ground truth, simulation‑based benchmark data sets complement experimental controls by providing a fully programmable, scalable, and reproducible framework for algorithm validation [39, 596, 597, 598, 599, 600]. These synthetic data sets were generated by modeling the entire sequencing workflow, from RNA molecule sequences and nanopore current signals to basecalling and downstream analyses, offering several unique advantages. Specialized tools can simulate not only nucleotide sequences but also the characteristic electrical signal perturbations induced by base‑level modifications and structural variations [596, 598, 599]. Together, these capabilities provide complete control over ground truth, ensure scalability and reproducibility, and enable isolated investigation of specific sources of error [599]. Consequently, simulation‑based data constitute a standardized, fully controllable, and cost‑effective resource for sequencing algorithm development, analytical workflow validation, and platform optimization [599]. This approach typically follows two complementary tracks, encompassing base‑aware sequence simulation and electrical signal‑aware current simulation.

Base‑aware simulation focuses on generating nucleotide sequences that closely approximate real sequencing data while reproducing both intrinsic biological features and technical biases introduced by experimental workflows. NanoSim is a data‑driven ONT read simulator that learns empirical models of read‑length distributions, error profiles, and sequence‑context‐dependent biases from real ONT data sets to generate highly realistic DNA, cDNA, and RNA reads [601]. Trans‑NanoSim extends this framework to transcriptome sequencing by explicitly modeling transcript abundance, AS, and RNA‑specific characteristics, enabling accurate simulation of ONT RNA‑seq data [600]. PBSIM3 supports sequence simulation for whole‑genome and transcriptome sequencing on both PacBio and ONT platforms, including ONT direct RNA and direct cDNA protocols, by applying long‑read error models to transcriptome references [602]. However, it offers limited customization for RNA‑specific features such as poly(A) tail length variation. Badread is a flexible long‐read simulator designed to generate long reads (PacBio and ONT) with user‐controlled error rates, read lengths, chimeras, and sequencing artifacts. Unlike data‐driven simulators, it prioritizes speed and stress‐testing of analysis pipelines over learning detailed empirical error models from real data [603].

DeepSimulator is a deep learning‐based ONT sequencing simulator that performs end‑to‑end modeling from DNA sequences to basecalled reads by generating synthetic electrical signals and associated base‑calling errors [596]. It captures context‑dependent relationships between nucleotide sequences and ionic current signals through pore‑aware models and incorporates mixed alpha distributions and Gaussian noise to realistically model signal resampling and experimental noise. However, it does not explicitly model RNA‑specific or DRS‐specific properties. Squigulator is a lightweight signal‐level simulator that converts nucleotide sequences into synthetic nanopore current traces using configurable noise and dwell‐time models [599]. It is sequence‐agnostic and fast, making it useful for algorithm testing and signal‐processing development, but it lacks data‐driven modeling of RNA biology and native DRS signal characteristics. Seq 2Squiggle is a neural‐network‐based framework that predicts nanopore current signals directly from nucleotide sequences using learned sequence‐to‐signal mappings. While it can produce realistic squiggles when trained appropriately, existing models are typically trained on DNA or cDNA data, so accurate ONT DRS simulation requires retraining with native RNA signal data [597]. Compared with traditional k‑mer‐based statistical models, its simulated signals exhibit substantially higher similarity to experimentally observed data, providing high‑fidelity training resources for the development and benchmarking of DRS algorithms.

Simulating RNA remains more challenging than DNA due to its secondary structure, diverse chemical modifications, slower nanopore translocation, and limited modeling of modification‑specific signal perturbations. Consequently, no fully validated end‑to‑end DRS simulator currently provides base‑sequence realism, RNA‑modification awareness, and isoform‑resolved ground truth. Fully realizing the potential of DRS for epitranscriptomics and single‑molecule RNA biology will require deeper integration of RNA‑specific biological features and advances in multimodal simulation frameworks (Figure 8 and Table S5).

### Integrating annotations, short‑read data, and perturbations for DRS validation

While ERCCs and IVT spike‑ins provide precise experimental ground truth, comprehensive reference annotations and epitranscriptomic databases supply essential orthogonal biological context for cross‑validation and functional interpretation. For example, standard gene annotations in GTF/GFF format from RefSeq [604], Ensembl [605], or GENCODE [606] define canonical exon‐intron structures, UTR, TSS, and TTS, enabling accurate evaluation of feature identification accuracy and performance in DRS data. In addition, specialized databases of transcription start and termination sites, such as refTSS [607] and PolyA_DB [608], integrate evidence from multiple experimental assays and provide critical references for determining whether DRS‑derived transcript boundaries are biologically plausible or instead reflect technical artifacts. For RNA modification analysis, curated public databases, such as RMBase [521], DirectRMDB [240], MODOMICS [609], and REDIportal [610], aggregate millions of modification sites identified by diverse experimental technologies. Overlap between DRS‑called sites and high‑confidence entries in these resources provides a strong positive prior, whereas systematic calls in genomic regions lacking modification evidence across large‑scale compendia may indicate model bias or systematic error. Moreover, integration with regulatory information (e.g., RNA‑binding protein sites, miRNA targets, and histone marks) enables assessment of whether putative false positives preferentially occur in structurally complex or regulatory‑dense regions, providing an additional layer of biological plausibility checking for DRS analyses [60, 611]. Together, these reference resources enable a more nuanced and biologically grounded evaluation of DRS performance.

Furthermore, short‑read sequencing data generated from the same samples as DRS provide high‑resolution, low‑error orthogonal evidence, making them powerful cross‑validation layers for benchmarking. For instance, conventional RNA‑seq robustly identifies exon‐intron boundaries, splice junctions, and exon usage at high depth, and comparative analyses consistently show that short reads detect substantially more splice junctions than long reads, whereas long reads better resolve full‑length isoforms, highlighting their complementarity for evaluating transcript models and differential exon usage [35, 296, 382, 612, 613, 614, 615, 616]. Cap‑dependent 5′‑end assays, including CAGE [617], SLIC‑CAGE [618], ReCappable‑seq [619], csRNA‑seq [620], and TT‑TSS‑seq [621], provide single‑nucleotide‐resolution maps of TSS. When integrated with DRS data, these resources enable systematic benchmarking of TSS detection accuracy by validating inferred start sites, identifying missed alternative promoters, and flagging spurious 5′ ends arising from truncation or mis‑priming artifacts. Similarly, 3′‑end‐focused methods and computational pipelines (e.g., PAS‐Seq [622], Term‑seq [623], RNAtag‑seq‐based approaches [624, 625], and RNA‑seq‐derived TTS inference) generate genome‑wide maps of TTS and 3′ UTRs. These enable systematic validation of DRS‑derived TTSs and PAS, as well as identification of alternative termination events that may be misassigned or missed by long reads. For RNA modification, short‑read immunoprecipitation‑ or chemistry‑based assays (e.g., m^6^A‑seq [17, 18], miCLIP [20], m^6^A‐LAIC‐Seq [130], GLORI [155], and related epitranscriptomic databases [521, 626]) provide population‑level enrichment maps. Overlap between DRS‑called modification sites and high‑confidence short‑read peaks increases confidence, whereas systematic DRS calls outside any enrichment regions help quantify false positives. Together, matched short‑read data sets provide independent, low‑error evidence for exon‐intron structure, TSS, TTS, and modification‑enriched regions or base‐pairs, enabling context‑stratified benchmarking and the identification of systematic DRS biases such as coverage dropouts, end truncation, or context‑specific miscalls. Combined with spike‑ins, simulation, and reference annotations, they form a robust, multilayer benchmarking framework for DRS.

Finally, appropriate genetic and enzymatic perturbations provide causal validation of DRS‐derived features. Including gene knockouts and enzyme‐treated samples alongside wild‐type greatly strengthens verification of DRS analyses, especially for RNA modification detection and interpretation. For example, knockdown or knockout RNA‑modifying enzymes (writers like METTL3/METTL5, or corresponding erasers like FTO, ALKBH5) should abolish or reduce the DRS modification signal at their target sites, providing direct causal evidence that a DRS‑called site is genuine [627, 628, 629, 630]. METTL3 and ALKBH5 perturbations have been used to confirm m^6^A sites called from DRS or short‑read‐based models, with loss/gain of signal at DRACH motifs supporting true sites [52, 627]. Targeted nanopore DRS of rRNA, coupled to CRISPR‑engineered loss of specific rRNA modification enzymes, allows direct comparison of WT versus KO DRS signatures at nucleotide resolution, enabling confident assignment of multiple rRNA modification classes without prior site knowledge [631]. Similarly, enzyme‑based removal or oxidative tagging approaches, such as FTO‑assisted m^6^A selective chemical labeling (m^6^A‑SEAL) and DNAzyme‑based methylation profiling of RNA (DAMP‑RNA), generating controlled gain, loss, or relabeling of specific RNA modifications [22, 116, 632, 633]. These assays provide orthogonal measurements that can be directly compared with DRS‑inferred modification presence and stoichiometry, enabling validation and refinement of nanopore‑based epitranscriptome maps. In brief, using gene knockouts and enzyme‑treated controls alongside DRS offers causal, site‑specific evidence that strongly verifies modification calls and improves confidence in DRS‑based epitranscriptome maps (Figure 8 and Table S5).

Taken together, ERCCs, IVT controls, and simulation‑based data sets provide precise experimental and computational ground truth for DRS benchmarking, but cannot fully capture endogenous RNA complexity. Complementing these with knockout or inhibitor perturbations, orthogonal NGS assays, and curated resources such as DirectRMDB supplies the independent biological context needed for robust cross‑validation and biologically grounded interpretation of DRS performance.

### Multilayer benchmarking metrics for DRS

Because nanopore DRS spans multiple analytical layers, from raw ionic current to high‑level transcriptomic features, rigorous benchmarking is essential for evaluating DRS methods. As no single reference provides absolute ground truth across all stages, effective benchmarking relies on cross‑validation using experimental spike‑ins, simulation‑based standards, and orthogonal sequencing technologies, with metrics tailored to each analytical layer.

At the raw current signal analytical level, benchmarking focuses on signal fidelity and basecalling accuracy. Core metrics include per‑base error rates, substitution, insertion, and deletion frequencies, and error profiles stratified by nucleotide context, homopolymer length, and neighboring base composition [40, 41, 54, 241, 334, 634]. In DRS, length‑dependent error profiling is particularly important, as systematic 3′ bias, premature read truncation, and signal decay toward the 5′ end directly affect transcript completeness and downstream feature calling [54, 480, 635, 636]. Modified and unmodified isogenic spike‑ins provide essential controls for evaluating modification‑associated basecalling errors, including systematic substitutions, elevated indel rates, or local confidence drops around modified residues. Additional benchmarking approaches include comparing per‑read quality scores with empirical error rates, assessing calibration of basecaller confidence estimates, and evaluating the robustness of basecalling across RNA length, sequence complexity, and modification density [54, 114].

Alignment benchmarking evaluates how accurately basecalled reads are mapped to reference sequences and how mapping uncertainty propagates to downstream analyses. Standard metrics include overall mapping rate, unique versus multimapping fractions, mismatch and indel rates in aligned regions, soft‑clipping frequency, particularly at 5′ ends, and the incidence of chimeric or split alignments [637, 638, 639]. Because DRS reads often exhibit truncation and variable error profiles, alignment accuracy should be evaluated as a function of read length, sequence context [496, 640, 641]. Synthetic controls and cross‑species spike‑ins are frequently used to estimate false‑positive alignment rates and reference ambiguity [563, 642, 643, 644]. Benchmarking also commonly assesses positional accuracy at transcript boundaries, such as deviations between aligned read ends and known transcription start or termination sites [645, 646, 647]. Comparative evaluation of splice‑aware and splice‑agnostic aligners, as well as RNA‑specific versus DNA‑derived alignment heuristics, is particularly important, as alignment choices can strongly influence isoform reconstruction, junction recall, and false‑junction rate [645, 647, 648].

Feature calling represents the primary analytical value of DRS and therefore requires task‑specific benchmarking strategies. For TSS and TTS detection, predicted sites are compared with orthogonal references such as CAGE‑seq or PolyA‑seq using strand‑aware distance thresholds (e.g., ±10−50 bp) [621, 649, 650, 651]. Performance is typically summarized using precision, recall, F1 score, positional bias, and clustering consistency across replicates [619, 649, 652]. Isoform reconstruction accuracy is evaluated by structural concordance with known transcript models or IVT standards, reporting sensitivity and precision at the transcript, exon, or splice‑junction level [13, 90, 219, 496, 653, 654]. Quantitative benchmarking compares observed expression estimates with known input concentrations from spike‑ins or synthetic mixtures. Pearson or Spearman correlation coefficients are commonly reported but are insufficient alone and should be complemented by metrics such as mean absolute log fold‑change, dynamic range, detection limits, and coefficient of variation across replicates.

RNA modification detection is typically evaluated using modified and unmodified isogenic controls, reporting receiver operating characteristic (ROC) and precision‐recall (PR) curves, alongside false‑positive rates estimated from negative controls, and stoichiometry estimation accuracy, positional uncertainty, and sensitivity to neighboring sequence context [56, 62, 114, 116, 241, 655]. Similar task‑specific frameworks apply to poly(A) tail length estimation, circular RNA detection, and RNA structure inference, each requiring tailored ground truth and orthogonal validation [537, 656, 657, 658, 659].

Importantly, no single metric or benchmarking layer is sufficient to characterize DRS performance. Effective evaluation therefore requires consistent performance across analytical layers and validation sources, with explicit reporting of failure modes such as truncation bias, context‑specific miscalls, or systematic alignment errors. Together, this multimetric, cross‑validated benchmarking framework enables biologically grounded assessment of DRS methods, facilitates fair comparison between tools, and ensures that reported advances reflect genuine methodological progress rather than data set‑specific optimizations (Figure 8 and Table S5).

## APPLICATION‑ORIENTED INTEGRATION OF NANOPORE DRS WITH MULTI‑OMICS

A comprehensive understanding of gene regulation across various conditions requires integration across multiple molecular layers, spanning genetics, chromatin accessibility, transcription, RNA processing, RNA modification, translation, metabolism, and protein abundance. While genomics and transcriptomics provide information on genetic potential and RNA expression, they often fail to capture the posttranscriptional and post‐translational regulatory processes that ultimately determine cellular phenotype. DRS occupies a unique position within this landscape by enabling native, full‐length interrogation of RNA molecules, thereby serving as a critical bridge between upstream regulatory inputs and downstream functional outputs. Integrating DRS with other omics modalities offers a powerful framework for constructing coherent, mechanistic models of cellular regulation.

### Genome‐transcriptome integration and regulatory input‐output mapping

**Figure 9:**
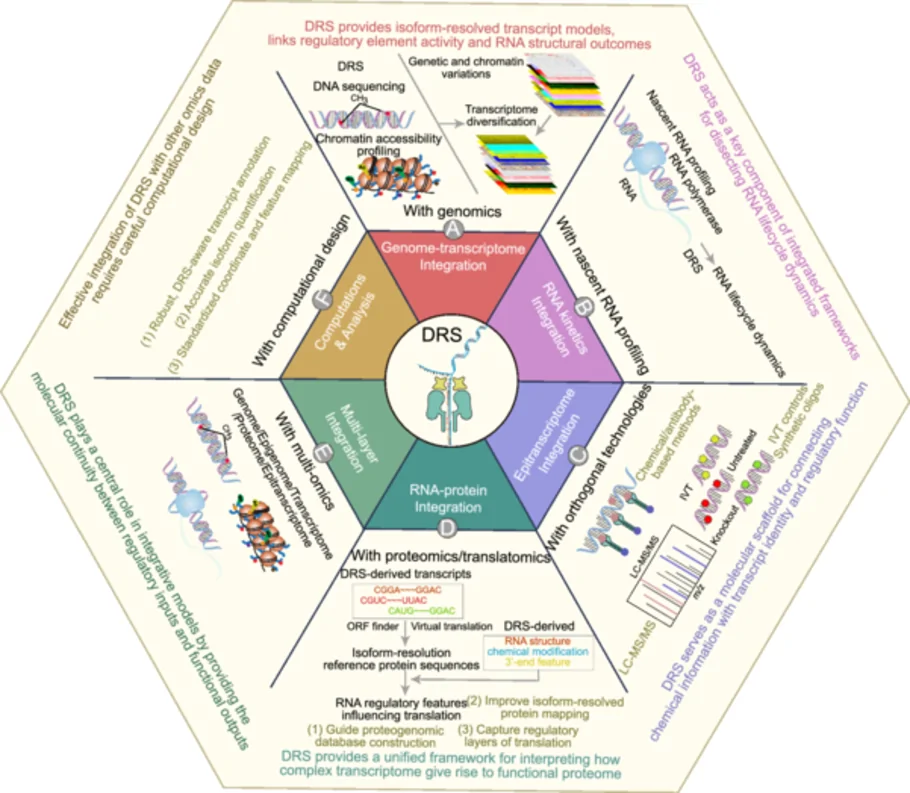
Nanopore direct RNA sequencing as a central hub for multiomics integration. (A) In the integration with genomics, DRS provides isoform‐resolved transcript models, links regulatory element activity and RNA structural outcomes. (B) In the integration with nascent RNA profiling, DRS acts as a key component of integrated frameworks for dissecting RNA lifecycle dynamics. (C) In the integration with orthogonal technologies for RNA chemical modifications, DRS serves as a molecular scaffold for connecting chemical information with transcript identity and regulatory function. (D) In the integration with proteomics, DRS provides a unified framework for interpreting how complex transcriptome gives rise to functional proteome. (E) In the integration with multiomics, DRS plays a central role in integrative models by providing the molecular continuity between regulatory inputs and functional outputs. (F) Effective integration of DRS with other omics data requires careful computational design.

One of the earliest demonstrations of DRS‐enabled multiomics integration was at the genome‐transcriptome interface. By combining ONT DRS with long‐read DNA sequencing, it became possible to simultaneously resolve genome architecture and native transcriptome regulation within a single analytical framework (Figure 9A). In yeast, the integration of DRS with PacBio long‐read genome assembly enabled concurrent decoding of genomic structure, differential gene expression, DNA methylation, and RNA modification landscapes, illustrating how genetic variation and chromatin‐level features are translated into mature RNA outputs [660]. This study established a conceptual blueprint for genome‐to‐transcriptome mapping in which DRS provides the missing link between DNA sequence and RNA regulatory state.

Building on this foundation, more recent multiomics strategies have integrated DRS with chromatin accessibility profiling and srRNA‐seq to interrogate transcriptional regulation in complex mammalian systems [367]. By combining assay for transposase‐accessible chromatin using sequencing (ATAC‐seq) with native full‐length RNA sequencing, these approaches systematically delineated tissue‐specific RNA landscapes across mouse organs, uncovering thousands of previously unannotated transcripts and organ‐specific isoforms. Importantly, these studies revealed that chromatin accessibility patterns alone are insufficient to predict mature transcriptomes, as extensive transcriptome diversification arises from downstream RNA processing. DRS was essential in this context, providing isoform‐resolved transcript models that could not be reconstructed from srRNA‐seq data and enabling direct linkage between regulatory element activity and RNA structural outcomes.

### Integration with RNA lifecycle kinetics and nascent RNA profiling

Beyond static transcriptome annotation, DRS has become a key component of integrated frameworks for dissecting RNA lifecycle dynamics. RNA processing, stability, and decay are inherently temporal processes, yet conventional transcriptomic methods capture only steady‐state snapshots. By coupling DRS with metabolic RNA labeling and machine learning‐based signal analysis (Figure 9B), recent studies have enabled direct identification of newly synthesized RNA molecules and quantitative assessment of nascent RNA processing at isoform resolution [197, 370, 661, 662, 663]. These integrated approaches demonstrated that DRS can distinguish newly transcribed RNAs from pre‐existing molecules based on characteristic signal features, allowing direct measurement of splicing kinetics, RNA maturation rates, and stability determinants without indirect inference. In particular, the ability of DRS to simultaneously capture transcript structure and poly(A) tail length has proven critical for understanding how isoform‐specific 3′‐end features influence RNA lifespan across cellular conditions.

Further expansion of this kinetic framework has been achieved through integration of DRS with complementary time‐resolved sequencing strategies such as TimeLapse‐seq [664]. By combining DRS with chemically encoded temporal information, these multicompartment approaches provide a systems‐level view of RNA flow across nuclear and cytoplasmic compartments. Such analyses reveal coordinated regulation of RNA processing, export, and decay, highlighting how transcript architecture and epitranscriptomic features jointly shape RNA fate in space and time.

### Epitranscriptomic and chemical‐layer integration

DRS‐based integration has increasingly extended into the epitranscriptomic domain, where RNA chemical modifications introduce an additional and highly dynamic layer of posttranscriptional regulation. When used alone, nanopore DRS robustly detects modification‐associated perturbations in ionic current signals on native RNA molecules, enabling transcriptome‐wide discovery of candidate modification sites. However, signal‐based detection by itself is inherently limited in chemical specificity and in controlling false positives arising from sequence context, RNA structure, or basecalling noise.

These limitations are effectively addressed through integration of DRS with orthogonal biochemical, genetic, and mass‐spectrometric approaches, yielding comprehensive, isoform‐resolved, and chemically defined epitranscriptomic maps (Figure 9C). In particular, coupling DRS with chemical‐ or antibody‐based NGS methods, such as MeRIP‐seq, miCLIP, bisulfite sequencing, or ac^4^C‐seq, enables validation of candidate modification sites and assignment of modification classes at single‐nucleotide resolution [33, 116, 387]. These approaches provide critical base‐level specificity that complements the long‐range contextual information captured by DRS. Mass spectrometry‐based methods, including LC‐MS/MS, further strengthen epitranscriptomic integration by directly measuring the chemical identity and global abundance of RNA modifications. Such measurements serve as gold‐standard validation for DRS‐inferred modification sites and regulatory circuits [256, 631], particularly for heavily modified RNA species such as tRNAs, where defined modification patterns (e.g., within the T loop) can be independently confirmed. Together, DRS and mass spectrometry bridge molecular‐scale chemical precision with transcriptome‐scale structural context.

Genetic and experimental controls are equally essential for robust integration. IVT unmodified RNAs, as well as writer knockouts or knockdowns, provide critical negative controls that distinguish true modification signals from sequence‐ or context‐dependent artifacts and enable calibration of detection thresholds [162, 177, 246, 256, 387]. These strategies are particularly important for training and benchmarking modification‐calling algorithms, where false positives can otherwise propagate systematically. More recently, synthetic oligonucleotides and engineered epitranscriptomes containing defined combinations of RNA modifications have emerged as valuable sources of ground‐truth training data. Such resources have enabled the development of multimodification detection frameworks, including TandemMod and updated Dorado models, improving the accuracy of simultaneous detection of multiple modification types from single DRS data sets [177, 189]. These advances highlight the importance of combining experimental design with algorithmic innovation in epitranscriptomic analysis. A key advantage of DRS‐based integration lies in its ability to preserve transcript‐level context. By maintaining full‐length RNA information, DRS allows chemical modification states to be associated with specific transcript isoforms, processing states, and expression levels, relationships that are largely inaccessible to short‐read or site‐centric approaches. This capability enables direct interrogation of how epitranscriptomic modifications intersect with AS, APA, RNA stability, and translation.

Collectively, these integrated strategies exemplify how DRS functions as a molecular scaffold that connects chemical information with transcript identity and regulatory function. Rather than replacing established epitranscriptomic methods, DRS complements them by embedding chemical modification data within data transcript architectures, thereby enabling a mechanistically grounded and systems‐level understanding of RNA regulation.

### RNA‐to‐protein integration through DRS: From proteogenomic databases to translational regulation

Bridging transcriptomic complexity with proteomic output remains a central challenge in functional genomics and life science research. Although high‐throughput proteomics has matured rapidly, its interpretability is fundamentally constrained by the quality of transcript‐derived protein sequence databases and by incomplete resolution of transcript isoform diversity. DRS offers a transformative framework for RNA‐to‐protein integration by providing native, full‐length transcript information that directly informs proteogenomic database construction, isoform‐resolved protein mapping, and mechanistic dissection of translational regulation (Figure 9D).

Accurate proteogenomic analysis depends critically on the completeness and correctness of the protein sequence database used for mass spectrometry searches. Conventional proteogenomic pipelines typically rely on reference transcript annotations or srRNA‐seq‐derived assemblies, which often fail to capture condition‐specific isoforms, truncated transcripts, alternative reading frames, or noncanonical translation events. As a result, many peptides detected in proteomic experiments remain unmapped or are ambiguously assigned. DRS fundamentally improves proteogenomic database construction by directly defining full‐length transcript isoforms without reliance on computational assembly. In *Arabidopsis*, incorporation of transcript isoforms derived from PacBio Iso‐seq and ONT DRS into proteogenomic search databases substantially increased proteoform identification in bottom‐up mass spectrometry compared with databases built from curated transcript annotations alone [665], underscoring the added value of DRS‐resolved transcript diversity for proteomic analyses. Importantly, accumulating evidence from eukaryotic, viral, and bacterial systems indicates that protein search databases derived from DRS data can explicitly encode alternative translation initiation sites [52, 207, 666], premature termination events [667, 668], intron‐retention‐derived coding sequences [97, 668], and previously unannotated [666, 669, 670], including small, open reading frames, many of which are increasingly supported by proteomic evidence of active translation. By anchoring proteogenomic searches to native transcript structures, DRS increases peptide identification rates and reduces false‐negative discoveries, thereby enhancing the depth and accuracy of proteomic analyses.

Beyond database construction, DRS enables more precise mapping of detected peptides to their transcript of origin, addressing a major limitation of traditional RNA‐protein integration. Many protein‐coding genes produce multiple transcript isoforms that encode highly similar or partially overlapping protein products. Short‐read transcriptomics often cannot distinguish these isoforms reliably, leading to ambiguity in assigning peptides to specific transcript variants. DRS resolves this ambiguity by providing isoform‐resolved transcript definitions that can be directly linked to protein sequences [81, 86, 90, 209]. When integrated with mass spectrometry data, this enables isoform‐level protein inference, allowing peptides to be mapped to specific transcript variants rather than collapsed at the gene level [207, 665, 671]. Isoform‐complete transcript definitions can be directly translated into protein sequences, enabling shared peptides to be correctly assigned to the expressed isoform while facilitating discovery and targeted analysis of isoform‐unique peptides [209, 665, 671]. This approach directly resolves the ambiguity arising from genes that produce highly similar and overlapping protein products. Such resolution is critical for understanding functional diversification, as isoform‐specific differences can affect protein domains, interaction motifs, subcellular localization signals, or post‐translational modification sites.

A further strength of DRS‐based RNA‐to‐protein integration lies in its ability to capture RNA regulatory features that directly influence translation but are largely invisible to conventional transcriptomic approaches. Translation is not determined solely by coding sequence abundance; it is modulated by untranslated regions, RNA secondary structure, chemical modifications, and 3′‐end features that collectively shape ribosome recruitment and translational efficiency. DRS provides access to several of these regulatory layers simultaneously. DRS simultaneously resolves alternative PAS, 3′ UTR isoforms, and poly(A) tail length at the level of individual RNA molecules [52, 60]. APA remodels the 3′ UTR regulatory landscape, modulating access of RNA‐binding proteins and microRNAs and thereby influencing mRNA stability, subcellular localization, and translational efficiency. In parallel, poly(A) tail length, often inversely correlated with steady‐state mRNA abundance and sensitive to epitranscriptomic perturbations such as METTL3 depletion [60], directly shapes the efficiency with which transcripts are translated into protein. The 5′ and 3′ UTRs harbor powerful cis‐regulatory elements that govern translational control. Studies using synthetic and native 5′ UTR libraries have demonstrated more than 100‐fold variation in ribosome recruitment and translation initiation, driven by short sequence motif, RNA secondary structures, and upstream open reading frames (uORFs). Likewise, 3′ UTRs, particularly their structural features, exert a strong influence on translation efficiency and serve as major hubs of posttranscriptional regulation. By preserving complete UTR context, DRS enables these regulatory features to be directly linked to isoform‐specific proteomic output. m^6^A deposition within 3*′* untranslated regions influences transcript abundance, 3*′*‐end formation, and downstream phenotypes, such as circadian period regulation. Transcriptome‐wide DRS analyses further reveal coordinated relationships among RNA methylation, stability, poly(A) tail dynamics, and AS, collectively reshaping translational efficiency and protein output. Transcriptome‐wide RNA structure probing and modeling demonstrate that secondary structure, particularly within 5′ and 3′ UTRs, is a strong determinant of translational efficiency. Integrating such structural information with isoform‐resolved DRS and quantitative proteomics enables direct evaluation of how defined RNA structural states modulate protein output.

Together, these advances position DRS as a central enabling technology for RNA‐to‐protein integration. By guiding proteogenomic database construction, improving isoform‐resolved protein mapping, and capturing regulatory layers relevant for translation, DRS provides a unified framework for interpreting how complex transcriptomes give rise to functional proteomes. As proteomic depth and translatomic resolution continue to improve, DRS‐based integration is expected to play an increasingly important role in elucidating post‐transcriptional regulation and its contribution to disease mechanisms and therapeutic responses.

### Multilayer integration for regulatory network reconstruction

Beyond pairwise integration, the full potential of nanopore DRS emerges when it is integrated with multiple omics modalities to reconstruct RNA‐centered regulatory networks (Figure 9E). Single‐molecule DRS enables joint analysis of AS, epitranscriptomic decoration (such as m^6^A and m^5^C), RNA stability, and transcript abundance within the same read, revealing coordinated crosstalk among RNA regulatory layers that are otherwise analyzed in isolation [33, 39, 52, 60, 179]. Within such multilayer frameworks, different omics modalities contribute complementary regulatory information, including chromatin accessibility defines transcriptional potential and regulatory input space, DRS captures mature transcript architecture and epitranscriptomic state, and proteomics quantifies protein output. Anchoring these layers on native RNA molecules enables mechanistic tracing of regulatory information flow from genome to phenotype. Empirical studies illustrate the power of this approach. In mouse organs subjected to fasting and feeding, integration of DRS with ATAC‐seq and srRNA‐seq uncovered thousands of previously unannotated transcript isoforms, along with tissue‐specific shifts in poly(A) tail length and m^6^A patterns linked to metabolic pathways [367]. These findings demonstrate how chromatin accessibility and RNA processing are coordinated at the organ level to drive metabolic adaptation. Similarly, in bacterial and plant systems, integration of DRS‐based modification calls with MeRIP‐seq and Illumina RNA‐seq has refined transcriptome‐wide m^6^A maps and revealed relationships between RNA modification, operon organization, transcript abundance, and RNA decay dynamics [33, 52, 208]. Computational advances further extend these integrative frameworks. Deep learning‐based models, such as SingleMod and pum^6^a, infer single‐molecule RNA modification patterns directly from DRS signal data and use these features to explain effects on splicing, RNA stability, and stress‐responsive gene expression programs, including those relevant to cancer [179, 405]. These approaches exemplify how DRS‐derived chemical and structural information can be incorporated into predictive models that bridge RNA chemistry with gene expression phenotypes.

Across these applications, DRS plays a central role by providing molecular continuity between regulatory inputs and functional outputs. Because DRS preserves transcript identity across regulatory layers, it enables consistent mapping of information from DNA and chromatin state to RNA processing, translation, protein production, and metabolic outcome. This continuity is particularly valuable in complex regulatory scenarios where perturbations at one layer may be buffered, amplified, or rewired at another, obscuring causal relationships. Current multiomics gene regulatory network (GRN) and enhancer‐GRN frameworks typically integrate chromatin accessibility, transcription factor binding, transcriptomics, and occasionally proteomics to infer regulatory interactions. A recurring theme in these studies is the need to extend such models with additional molecular layers and more quantitative, causally informative measurements. DRS naturally fulfills this role by serving as the RNA continuity layer that connects chromatin and transcriptional regulation with downstream proteomic states. Incorporation of DRS into multiomics network reconstruction therefore represents a key advance toward mechanistically grounded and predictive models of gene regulation.

### Computational and analytical considerations

Effective multiomics integration involving nanopore DRS critically depends on DRS‐aware preprocessing and rigorous harmonization across a shared, isoform‐resolved coordinate system (Figure 9F). Substantial differences in data resolution, noise characteristics, and sampling depth across omics modalities pose nontrivial analytical challenges. DRS data are inherently long‐read and signal‐rich, capturing molecule‐level information on transcript structure, poly(A) tails, and RNA modifications, whereas most complementary omics assays, including srRNA‐seq, ATAC‐seq, ribosome profiling, and proteomics, produce fragmented or feature‐based measurements. Bridging these fundamentally different data representations requires robust transcript annotation, accurate isoform quantification, and standardized feature mapping. Several characteristics of DRS complicate direct integration. Compared with short‐read sequencing, DRS exhibits higher base‐level error rates and generates a substantial fraction of truncated or non‐full‐length reads, which can obscure transcript boundaries and complicate isoform assignment and quantification [84, 405, 640]. These challenges are particularly pronounced in complex mammalian transcriptomes and for lowly expressed isoforms, where read coverage is sparse [672]. In addition, long‐read data sets often show variability in TSS and PAS detection, making it difficult to define consistent transcript boundaries that are directly comparable across omics layers [646]. Limited sequencing throughput further constrains statistical power for differential analyses and complicates matching depth‐rich short‐read, ATAC‐seq, or proteomics data sets [84, 86, 640]. Finally, DRS reports per‐molecule signals and modification states, whereas most other omics data are summarized as aggregated genomic features, such as peak intensities or count matrices, creating an additional layer of abstraction that must be reconciled during integration [84, 614, 640, 665].

Robust, DRS‐aware transcript annotation therefore represents a foundational design principle for harmonized multiomics analysis. Long‐read‐optimized tools, including IsoQuant, Bambu, NAGATA, and SQANTI3, are essential for constructing high‐quality isoform catalogs that accurately capture transcript structure, splice junctions, and transcript ends [84, 209, 646, 672]. Where possible, these annotations should be supported by orthogonal evidence from short‐read RNA sequencing or specialized end‐mapping assays. Hybrid short‐ and long‐read pipelines are particularly effective, as they improve definition of transcript termini, strand assignment, and isoform structures, yielding a more reliable and biologically meaningful reference annotation [84, 613, 646]. This isoform catalog then serves as a common coordinate system onto which all other omics measurements can be projected. Accurate isoform quantification constitutes a second critical principle. DRS‐specific quantification tools, such as NanoCount, IsoQuant, and NanoTrans modules, are designed to exploit long‐read properties while filtering ambiguous alignments and partial reads [84, 86, 90, 206]. Benchmarking efforts, including the long‐read RNA‐seq genome annotation assessment project (LRGASP) framework, consistently demonstrate that reference‐based approaches using high‐quality isoform annotations yield the most reliable abundance estimates for integrative analyses [84]. Reliable quantification is essential not only for transcript‐level comparisons but also for linking RNA features to downstream translation and protein output. A third principle is standardized coordinate and feature mapping across omics layers. Rather than collapsing measurements at the gene level, integration pipelines should map ATAC‐seq peaks, chromatin immunoprecipitation sequencing (ChIP‐seq) signals, ribosome footprints, proteomics peptides, and metabolite‐associated genes onto the same isoform‐resolved annotation [84, 614, 665]. This strategy fully leverages the strengths of DRS by preserving proteoform‐specific and regulatory information that would otherwise be obscured by gene‐level aggregation.

Collectively, integrative DRS‐based multi‐omics analyses require analytical pipelines that first address DRS‐specific challenges in transcript annotation and quantification, and then systematically project all complementary omics data into a shared, isoform‐resolved coordinate space. By doing so, these frameworks mitigate differences in resolution, noise structure, and sampling depth across data types, enabling coherent and mechanistically interpretable reconstruction of RNA‐centered regulatory programs.

## CHALLENGES AND TECHNICAL IMPROVEMENTS OF DRS

Despite its ability to deliver a variety of high‐value analytical applications, the performance of DRS remains subject to sample type, library chemistry, nanopore properties, motor enzymes and basecalling models. Most related applications continue to rely heavily on model‐driven inference and can be significantly affected by sequencing coverage, signal quality and available training data. DRS should therefore be considered not as an all‐around or universally better substitute for traditional transcriptomic methods, but as a rapidly developing platform that shows clear strengths when native long‐read data are specifically required.

In this section, we summarize the major technical constraints that currently limit DRS, with emphasis on sample input, transcript coverage, signal interpretation, reproducibility, and computational generalizability. We then discuss recent improvement directions in chemistry, hardware, algorithms, and standardization. To avoid overinterpretation, we also propose a task‐oriented view of DRS outputs, distinguishing between readouts that are already relatively robust, features that remain strongly model‐dependent, and biological conclusions that still require extensive orthogonal validation.

### Current technical challenges in DRS

#### Sample input, library preparation, and transcriptome representation

DRS library preparation still requires relatively large amounts of input RNA. For example, the ONT SQK‐RNA004 workflow typically requires approximately 300 ng of poly(A)‐selected RNA or about 1 μg of total RNA, which remains challenging for many low‐yield samples such as plasma RNA, extracellular vesicle RNA, needle biopsies, rare clinical specimens, or highly specific micro‐dissected tissues [60]. Although multiplexing and targeted enrichment may partially alleviate this constraint, these solutions are not yet standardized across laboratories or application settings.

This limitation is even more pronounced in single‐cell and spatially resolved applications. The RNA content of a single cell is orders of magnitude below the input requirements of current commercial DRS kits, and low‐input processing introduces substantial risks of adsorption loss, degradation, contamination, and stochastic transcript dropout [534, 673, 674]. As a result, the application of DRS in single cell has not been developed to date.

#### Limited performance for short RNAs

DRS remains poorly suited for the short RNAs. Molecules shorter than roughly 100−150 nt are often inefficiently captured or confidently basecalled in standard workflows [60], even though many biologically important and clinically informative RNAs fall within or below this range. While recent updates to the MinKNOW software have improved detection sensitivity for RNA molecules longer than 50 nt, biologically relevant short RNAs, such as 22−45 nt cell‐free RNAs in liquid biopsies that serve as potential disease biomarkers, remain largely undetectable [63]. Currently, laboratory‐developed small‐RNA DRS protocols are promising, but they are not yet broadly standardized and continue to face challenges in reproducibility, adapter discrimination, and general applicability. While laboratory‐based non‐poly(A) enrichment methods exist, their lack of commercialization restricts transcriptome comprehensiveness and highlights the need for inclusive capture strategies [36, 192].

#### Limitations of DRS in throughput, accuracy, and completeness

Although DRS has certain advantages, benchmarking this approach against mainstream transcriptomic technologies, such as Illumina short‐read platforms, nanopore cDNA sequencing, and PacBio Iso‐Seq exposes inherent technical bottlenecks [81]. The most notable limitations revolve around base‐calling fidelity, 5*'* transcript integrity, and sequencing throughput [162, 189].

An elevated base error rate remains a fundamental constraint of the DRS platform. Although the incorporation of the SQK‐RNA004 sequencing kit has improved the overall read accuracy, the systematic errors remain largely identical compared to the previous kits. Some sequencing errors are attributable to signal insufficiency rather than algorithmic (basecalling) artefacts [54]. This performance contrasts starkly with NGS methodologies, nanopore cDNA sequencing, and modern PacBio Iso‐Seq that utilizes Circular Consensus Sequencing (CCS/HiFi) to suppress error rates [39]. The high frequency of stochastic errors in DRS severely complicates single‐nucleotide variant (SNV) calling [54, 675]. Insertion and deletion artifacts in homopolymeric regions are particularly problematic and generate a prohibitive background of false‐positive variants [54, 534, 676]. Therefore, for studies relying on high‐precision detection, such as SNV identification, it is still recommended to use high‐accuracy methods including NGS, nanopore cDNA sequencing, and PacBio Iso‐seq [191, 468, 675, 677]. DRS should be used in conjunction with these high‐precision approaches.

Transcript completeness presents another major hurdle. DRS elegantly circumvents artifacts introduced by reverse transcription and PCR amplification, yet it paradoxically struggles to capture true full‐length transcripts when compared to nanopore cDNA sequencing and PacBio Iso‐Seq [81, 84]. Researchers have well documented the systematic loss of coverage at the 5*'* terminus during DRS runs [646, 678]. This structural truncation occurs because the motor protein driving the RNA strand dissociates or accelerates prematurely as the tail end of the molecule translocates through the pore. The sensor consequently misses approximately 10 to 15 nucleotides at the 5*′* end [35, 60, 109, 191]. Pervasive in vitro RNA degradation during library preparation further exacerbates this physical limitation [481]. The resulting 5*′* incompleteness critically impairs downstream transcriptomic interpretations [84]. Assembly algorithms frequently misclassify degraded or systematically truncated 5*'* ends as novel alternative TSS [533]. This misinterpretation artificially inflates the perceived complexity of the transcriptome and hinders accurate isoform quantification. Moreover, although poly(A) tail profiling represents one of the more mature and accurate applications of DRS, particularly when using the Dorado basecaller (v0.9.0 or later), its reliability can still be affected by factors such as RNA degradation and transcript end integrity [468, 537]. Consequently, current poly(A) tail‐length estimates should not be regarded as a fully comprehensive readout for interpreting posttranscriptional regulation across all transcript contexts.

In addition, the overall sequencing depth of DRS is also relatively lower compared with NGS [81]. For tasks requiring high coverage, such as transcriptome assembly and annotation in novel species, cDNA‐based long‐read sequencing supplemented with NGS for error correction may be more appropriate [84, 612].

Taken together, these comparisons demonstrate that while DRS holds unique advantages in retaining native RNA information its higher base error rate and relatively lower transcript completeness compared with NGS, nanopore cDNA, and PacBio Iso‐seq restrict its utility in high‐precision applications such as SNV identification and comprehensive transcriptome profiling. In these scenarios, NGS‐based error correction would be helpful to enhance the reliability of DRS.

#### Ground truth limitations and unresolved challenges in RNA modification detection

Currently, there is a significant gap between expectations and routine practice in the field of epitranscriptome interpretation. Most DRS‐based modification callers infer modifications indirectly from deviations in ionic current intensity, dwell time, or basecalling behavior relative to learned expectations [655]. This creates several sources of uncertainty.

A fundamental challenge is the lack of robust, broadly accepted ground truth data sets for many RNA modifications. Unlike canonical sequence benchmarking, where reference genomes and validated variant sets are often available, modification calling typically depends on synthetic constructs, enzyme perturbation experiments, antibody‐based enrichment, or site‐specific orthogonal assays, each of which captures only part of the issue and introduces its own biases [116, 119]. As a result, these reported methods' performance may not transfer well across species, transcript contexts, chemistries, or laboratories. The second challenge is signal confounding. Modification‐associated signatures are influenced by k‐mer context, neighboring modifications, RNA secondary structure, translocation kinetics, and local noise [655]. Multiple nearby modifications may compress or distort the current profile, making deconvolution difficult [182]. This is particularly problematic for de novo discovery, where the search space is large and false positives can accumulate rapidly if candidate signals are not filtered against appropriate negative controls and matched reference backgrounds [34, 372]. The third challenge is quantification. Even when a site can be detected, accurate estimation of modification stoichiometry remains difficult, especially for low‐abundance transcripts or heterogeneous transcript isoforms [62, 372]. Current methods vary in whether they classify reads, positions, or transcript‐level events, and these analytical choices affect the biological conclusions that can be drawn [180]. This problem is even more pronounced for low‐abundance or low‐stoichiometry modifications, where limited read support and weak signal perturbation can jointly reduce sensitivity and precision, making confident detection particularly difficult in complex transcriptomes [62, 145]. Overall, the challenges that need to be addressed for the identification and quantification of RNA modifications using DRS include the construction of authentic and reliable training sets, the screening of effective prediction results, and the enhancement of comparability among different tools and models.

The rapid development of quantitative methods for RNA m^6^A modification based on chemical or enzymatic conversion combined with NGS, such as GLORI‐seq, has generated extensive and reliable quantitative data sets of m^6^A sites [155, 679]. These resources enable model training for DRS‐based m^6^A detection to avoid manual labeling of training sets. Models can be trained directly using m^6^A modification levels at corresponding sites as seen in methods like SingleMod [155, 179]. This progress has greatly improved the predictive performance of related models and serves as an important reason for the relatively high accuracy of DRS in m^6^A prediction [680]. Future development of identification and quantification models for RNA modifications using DRS should therefore prioritize the establishment of precise experimental approaches. Such methods can supply accurate and dependable training sets for model training and also act as an important means to validate model outputs. Effective filtering is required for prediction results generated by DRS models [179]. Preliminary screening can be performed following threshold combinations recommended by corresponding tools [62, 162]. Common filtering indicators include modification levels and sequencing coverage depth [162]. Stricter filtering criteria may also be applied according to specific research demands to achieve more reliable outcomes [119, 326]. Additional validation with the support of relevant databases and experimental methods is also helpful [521, 681]. GLORI‐seq for instance can be adopted to verify predicted m^6^A modification signals [21, 155].

To improve the comparability of prediction outcomes across different tools or models researchers suggest integrating results from multiple tools for site‐level identification [162]. Consistent sites identified through intersection analysis can be regarded as confident modification positions [162]. In addition, modification ratio from different models and tools vary greatly. Such discrepancies are closely associated with model training data sets [162, 580, 655]. Currently, this issue is tough to resolve. To avoid this issue, groups should utilize the same tool in the same experiment to get comparable results.

### Technological improvement directions of DRS

#### Hardware optimization

Hardware optimization containing sequencing chemistry, pore design, and motor enzymes, remains essential for DRS because many current analytical limitations originate upstream at the level of signal generation rather than downstream interpretation alone [54, 120].

In DRS, irregular motor stepping, incomplete control of RNA translocation, pore blockage by structured molecules, and chemistry‐dependent signal drift can all distort the ionic current profile before basecalling begins [39, 194]. These effects reduce signal‐to‐noise ratio, compress differences between similar k‐mers, and increase local instability at transcript ends or in structurally complex regions, thereby contributing to read truncation, base miscalls, and reduced confidence in modification‐associated signal shifts.

Improvements in pore proteins, motor enzymes, and sequencing chemistry are therefore needed not simply to raise nominal accuracy, but to produce cleaner and more uniform signal traces. More stable pore‐motor coupling and better‐controlled translocation can reduce dwell‐time variability and improve resolution of adjacent nucleotides, whereas motors with greater processivity and tolerance for structured RNA may lower blockage rates and improve read continuity across difficult regions [39]. In practical terms, these changes can enhance splice junction mapping, transcript boundary assignment, detection of subtle sequence variants such as RNA editing events, and discrimination among highly similar alleles, paralogs, or copy‐number‐related transcript copies [81, 682]. They may also improve the separability of modification signals from background noise, although this remains highly context dependent. Notably, these improvements should be interpreted as reducing specific technical bottlenecks rather than conferring uniformly high nucleotide‐level accuracy across all applications, and conclusions involving subtle variants or epitranscriptomic features still need orthogonal and cross‐platform validation [34]. For nucleotide‐level applications in which even small residual errors can alter biological interpretation, short‐read sequencing and other long‐read approaches remain essential reference frameworks.

### Algorithm optimization: From basecalling to scalable, uncertainty‐aware, and integrative modeling

The next stage of DRS development will depend not merely on adding new base or modification callers, but on establishing analysis frameworks that can (i) efficiently prioritize true signals from a large candidate space; (ii) distinguish chemically similar modifications under heterogeneous sequence contexts; (iii) quantify uncertainty explicitly; and (iv) connect read‐level molecular features to RNA structure, function, and regulatory networks [518]. These challenges are particularly acute for native RNA, where the observed current at any given position reflects not only the focal nucleotide but also its surrounding k‐mer context, neighboring modifications, translocation dynamics, and run‐specific noise.

The first priority is the development of scalable strategies for candidate screening and false‐positive control [62]. In practice, many current modification analyses still rely on broad signal scanning followed by threshold‐based calling, an approach that becomes increasingly unstable when the number of candidate sites is large, coverage is uneven, or multiple modification types may coexist within the same transcript [175, 683]. A more robust computational workflow will likely require multi‐stage filtering. One practical strategy is to combine a high‐sensitivity first‐pass detector with downstream evidence integration, in which candidate sites are retained only if they satisfy predefined criteria across several dimensions, such as read depth, replicate concordance, local signal consistency, transcript‐context plausibility, and contrast against matched negative controls or perturbation data sets. Hierarchical statistical models, empirical Bayes shrinkage, and replicate‐aware false discovery control may be especially useful here, because they can borrow information across sites or transcripts while preventing low‐coverage outliers from being overinterpreted [34, 185]. Such frameworks would be particularly valuable for large‐scale studies, where the main computational question is no longer whether some anomalous sites can be detected, but how to rank, filter, and validate thousands of weak candidate events without inflating false positives.

The second challenge is the discrimination of modifications with subtle or partially overlapping signal signatures. For example, closely related marks such as m^6^A and m^6^Am, or context‐dependent signatures that resemble pseudouridylation‐ or m^5^C‐associated perturbations, may not be separable by a single signal feature alone [390, 684]. Recent multi‐modification models, such as ORCA [503], DirectRM [180], and NanoSpeech [180], have advanced DRS analysis beyond one‐modification‐at‐a‐time calling by enabling unified prediction of multiple RNA modification classes from shared nanopore signal features. Their main strength is that they capture common and modification‐specific signal perturbations within a single framework, thereby improving the feasibility of multi‐class inference on native RNA. However, their performance remains limited by the restricted diversity and scale of available training data, which often fail to represent endogenous transcript contexts, variable stoichiometries, neighboring modifications, and chemistry‐dependent signal shifts. As a result, distinguishing closely related or partially overlapping modification states, such as m^6^A and m^6^Am, remains challenging, particularly when local RNA structure or adjacent modifications distort the signal. In addition, most current models still behave largely as closed‐set classifiers and may overassign ambiguous or out‐of‐distribution signals to known classes [518]. Future progress will therefore require context‐aware and uncertainty‐aware frameworks that integrate local signal features with transcript position, motif environment, cap proximity, and RNA structural information, while also incorporating calibrated probability outputs or abstention categories for unresolved cases. Equally important, these models should be evaluated not only by classification accuracy, but also by cross‐chemistry robustness, reproducibility across biological replicates, and consistency with orthogonal validation. Moreover, reliable information on modification sites obtained by methods based on NGS should be fully utilized in the construction of model training sets [179, 181].

The third requirement is explicit uncertainty modeling and cross‐platform validation. Because many DRS modification calls remain model‐dependent, algorithm outputs should ideally include confidence estimates at the read, site, and transcript levels [685]. This could be implemented through Bayesian formulations, ensemble prediction, conformal prediction, or calibration procedures that distinguish confident calls from ambiguous ones [162, 175, 579]. Such uncertainty estimates are not only statistically desirable; they are essential for determining which candidates should advance to orthogonal validation. In this context, computational analysis should be coupled more tightly to experimental design. For high‐confidence sites, validation may involve writer/eraser perturbation, synthetic or in vitro‐transcribed reference RNAs, class‐level mass spectrometry, or site‐directed biochemical assays, depending on the modification under study [181, 685]. Equally important is validation across analytical environments: a candidate signal that disappears after chemistry change, basecaller update, or remapping is unlikely to be a robust biological event. Future best‐practice pipelines should therefore treat reproducibility across biological replicates, software versions, and orthogonal assays as part of the calling framework itself rather than as an optional downstream step.

The fourth frontier lies in modeling combinatorial and context‐dependent RNA regulation at the single‐molecule level. At present, single‐molecule integrative modeling with DRS remains largely at the proof‐of‐principle stage rather than a routine analytical reality. Although DRS can in principle link multiple transcript features within the same native RNA molecule, this integration is still limited by the limitations outlined above, such as basecalling errors, systematic loss of 5*′* terminal information and relatively low sequencing throughput. In addition, these features are rarely measured with comparable coverage or confidence on the same molecules, making downstream network inference highly sensitive to missing data and error propagation [445]. Near‐term progress will therefore depend less on simply adding more data layers and more on improving the reliability of each layer and their cross‐modal alignment [189, 685]. Practically, this will require higher‐fidelity basecalling, transcript‐end recovery, and confidence‐aware modification calling with replicate and perturbation support. A realistic path forward is stepwise integration, beginning with better‐supported combinations, such as isoform‐poly(A) or isoform‐modification coupling, and then extending to RNA structure, RBP occupancy, translation, and stability through matched multi‐omics data sets and perturbation‐based validation.

Finally, multiomics integration will be essential for constructing hierarchical maps of RNA regulation. In future studies, DRS data should not be modeled only as an alternative transcriptomic readout, but as one molecular layer within a broader regulatory system that also includes genome variation, chromatin state, transcriptional activity, RNA processing, translation, and proteome output [503]. A practical analytical framework may involve three levels: first, read‐level inference of transcript structure, poly(A) tail features, and candidate modification states [179]; second, transcript‐ or gene‐level integration with abundance, allele, and structural information; and third, network‐level modeling that links these RNA features to upstream regulators and downstream phenotypes. This hierarchy may be especially important in complex biological settings such as development, stress adaptation, tumor evolution, or host‐pathogen interactions, where the functional significance of an RNA modification cannot be inferred from its presence alone [285].

Overall, the most pressing computational need is not only higher predictive accuracy, but a shift toward scalable candidate prioritization, uncertainty‐aware classification, reproducibility‐centered filtering, and mechanistically informed integration.

#### Standardization and benchmarking

Standardization and benchmarking are essential in DRS not only for technical harmonization, but because many biologically important outputs, especially modification calls, stoichiometry estimates, allele‐specific analyses, and even some transcript‐end assignments, remain highly sensitive to chemistry version, basecaller, alignment strategy, and training data [516, 686]. The central problem is therefore not the absence of a single universal benchmark, but the lack of task‐specific truth standards and evidence frameworks. Future benchmarking should be organized by analytical task, since transcript structure reconstruction, 5*′* and 3*′* end definition, poly(A) measurement, modification detection, stoichiometry estimation, and allele‐aware inference differ substantially in their error modes and validation requirements [81, 84]. A useful next step would be to establish community reference sets that combine synthetic RNAs, perturbation‐derived controls, and biologically matched samples processed across laboratories, chemistries, and software versions, so that both accuracy and robustness can be assessed explicitly. Just as importantly, DRS studies would benefit from an evidence hierarchy in which relatively direct readouts are distinguished from model‐dependent inferences and from higher‐level biological claims that require orthogonal validation. Such a framework would make cross‐study comparisons more meaningful, reduce overinterpretation of version‐specific results, and provide a more realistic basis for future clinical or regulatory adoption.

#### A task‐oriented evidence framework for interpreting DRS data

Given the uneven maturity of different DRS outputs, we suggest that interpretation should follow a task‐oriented evidence framework.

Tier 1: relatively robust readouts (High Confidence). These include long‐read‐supported transcript structures, broad APA patterns, poly(A) tail estimates with dorado software, and quantification of gene expression in sufficient depth and well‐behaved transcript regions [84, 200, 537].

Tier 2: model‐dependent inferences (Moderate Confidence). These include identification and quantification of well‐characterized RNA modifications (e.g., m^6^A), low‐abundance isoform quantification, splicing events, prediction of RNA structure, and RNA editing sites.

Tier 3: partial single‐base‐level output. These applications encompass SNV detection, transcription start site identification [506], as well as the identification and quantification of emerging RNA modifications that cannot be confidently resolved using NGS‐based methods [685].

This framework does not undermine the value or the substantial technological advancements represented by DRS; instead, it helps align data interpretation with current technical maturity and may mitigate overinterpretation in both basic and translational research. Moreover, the framework is not absolute. For example, alternative splicing sites identified by DRS show significantly higher confidence when cross‐validated with reliable platforms such as Illumina sequencing [84, 365]. Notably, this framework is only suitable for the present stage, and the corresponding classification will need to be adjusted accordingly as DRS technology continues to improve.

## OUTLOOK: TOWARD INTEGRATIVE, HAPLOTYPE‐AWARE RNA BIOLOGY

### From feature catalogues to multilayer regulatory logic

**Figure 10:**
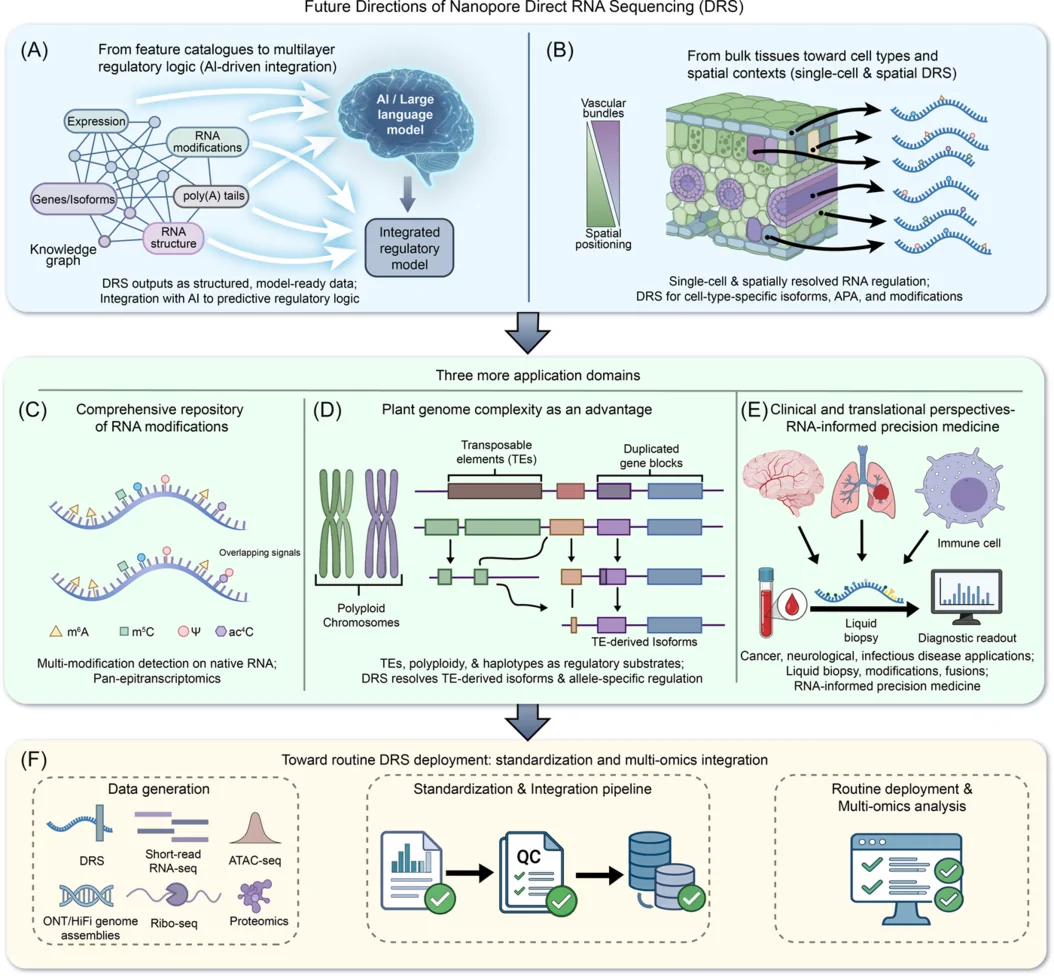
Outlook of nanopore DRS applications. (A) From feature catalogues to multilayer regulatory logic: DRS integrates isoforms, poly(A) tails, and RNA modifications into structured data frameworks compatible with AI and knowledge‐graph models. (B) From bulk to single‐cell and spatial contexts: emerging strategies aim to resolve cell‐type‐specific RNA regulation. (C) Expanding epitranscriptomics: multimodification detection beyond single marks enables integrative RNA state analysis. (D) Genome complexity as opportunity: DRS resolves TE‐derived isoforms and haplotype‐specific regulation. (E) Clinical translation: workflow harmonization and multiomics integration support scalable research and RNA‐informed precision medicine. (F) Standardization and multiomics integration form the foundation for routine and scalable DRS deployment. AI, artificial intelligence.

DRS has rapidly moved beyond proof‐of‐concept demonstrations toward integrative discovery, fulfilling a key early promise of the technology: the ability to read transcript structure, poly(A) properties, and multiple RNA modification signals simultaneously on native RNA molecules [51, 120]. This integrated readout can reduce experimental fragmentation compared with running parallel assays for isoforms, 3*′* ends, and epitranscriptomics, although the maturity and reliability of these individual readouts are not yet equivalent across applications [202]. Accordingly, DRS is increasingly being considered not only as an alternative to conventional RNA‐seq, but also as a potentially useful framework for studying multilayer RNA regulation in animal and plant transcriptomics. A practical application of this transition is timely as the output of DRS is unusually model‐ready. Each read naturally yields structured attributes, isoform identity, cleavage site choice, tail length estimates, and modification probabilities, that can be assembled into transcript‐ or gene‐level knowledge graphs. Such graphs may provide a useful substrate for representation learning and, in some settings, may be compatible with large language model (LLM)‐assisted biological reasoning, as has begun to be explored in crop‐focused knowledge graph frameworks [38, 687]. Standardized DRS‐derived feature sets could therefore serve as one possible bridge between experimental transcriptomics and AI‐assisted hypothesis generation, although the biological validity and practical utility of such frameworks will require careful benchmarking (Figure 10A).

### From bulk tissues toward cell types and spatial contexts

Bulk DRS studies consistently imply that RNA regulation is highly context‐specific, particularly for isoform switching, APA remodeling, and RNA modification dynamics. However, most current mechanistic inference still relies on tissue averages. Early long‐read single‐nucleus approaches in plants, although based on cDNA rather than native RNA, have already shown that AS and APA patterns differ sharply across cell populations, underscoring the value of long reads for resolving regulatory heterogeneity [688]. True single‐cell DRS remains constrained by input requirements, signal noise, and cost, particularly when modification inference is included (Figure 10B) [54, 120]. In the near term, a more realistic path may be hybrid strategies: use short‐read single‐cell or single‐nucleus data to define cell states and candidate regulatory programs, followed by bulk or targeted DRS in enriched tissues to characterize multilayer RNA features [689, 690]. Even without native single‐cell DRS, integrating bulk DRS with spatial transcriptomics or cell‐type marker frameworks may help associate regulatory programs with anatomical contexts, although such assignments will often remain indirect [691, 692].

### Accessing a comprehensive repository of RNA modifications

Epitranscriptomics has so far been dominated by m^6^A and m^5^C, yet accumulating evidence indicates that additional modifications, such as hm^5^C and ac^4^C, contribute to development, stress responses, and long‐distance RNA transport [693, 694]. DRS is particularly attractive in this context because it may, in principle, detect multiple modification signatures on the same native RNA molecules and relate them to isoform usage, APA decisions, and stability‐associated features. A key future direction is to move from single‐accession snapshots toward pan‐epitranscriptomes, mirroring the conceptual shift introduced by pangenomes [202]. If sufficiently robust and transferable analytical pipelines become available, population‐scale DRS could help distinguish conserved modification sites that form regulatory backbones from variable, genotype‐ or environment‐specific marks that may tune phenotypes (Figure 10C). Methodologically, this expansion depends on robust and transferable modification callers. Recent benchmarking and transfer‐learning studies suggest that nanopore signal perturbations induced by RNA modifications are sufficiently conserved to support cross‐species model adaptation [175, 177, 180]. However, whether this will generalize across modification classes, sequence contexts, and chemistries remains to be established. These developments therefore point toward, rather than yet deliver, a broader repository of RNA modifications across species. In particular, low‐abundance modifications and condition‐specific marks are likely to remain difficult to catalogue systematically until both signal sensitivity and orthogonal reference data sets improve substantially.

### Plant genome complexity as an advantage: TEs, haplotypes, and evolution

Plants are enriched for the two genome features that most challenge transcriptomics, TEs and polyploidy, but these same features make DRS particularly powerful. Long‐read DRS can directly resolve TE‐derived isoforms, chimeric gene‐TE transcripts, and alternative termination within repetitive contexts that confound short reads [276, 352]. These studies reveal TE‐derived transcripts are not annotation artifacts but regulatory entities shaped by RNAPII elongation, epigenetic state, and alternative poly(A) signal usage. In polyploid crops and diverse germplasm panels, DRS may also provide an allele‐ and haplotype‐aware readout, supporting analysis of ASE and, potentially, allele‐specific RNA modification and APA [202, 359]. If these applications become more robust, they could enable an evolutionary transcriptomics framework in which TE‐derived isoforms and allele‐specific RNA regulation are treated not merely as analytical noise, but also as possible substrates for adaptation (Figure 10D). Because polyploidization is pervasive across the plant kingdom, particularly in wild and nonmodel species [695], DRS may be especially useful for studying transcriptomic regulation in complex genomes where short‐read approaches are often limited.

### Clinical and translational perspectives: Possibilities of RNA‐informed precision medicine

DRS offers a powerful framework for future clinical diagnostics by enabling direct, amplification‐free profiling of full‐length native RNAs together with their modification states (Figure 10E). However, its translational relevance remains contingent on overcoming key challenges, including analytical sensitivity, clinical‐grade accuracy, reproducibility, and standardized workflows compatible with regulatory requirements [54, 189]. If these barriers can be addressed, DRS could contribute to a gradual shift in some settings from static, gene‐centric measurements toward more dynamic, RNA‐centered molecular profiling [696, 697].

In oncology and liquid biopsy applications, DRS may eventually help resolve oncogenic fusion transcripts, tumor‐specific splice isoforms, and possibly aspects of RNA modification variation from limited circulating RNA inputs, although these use cases still require substantial validation and sensitivity improvements [698, 699]. In neurological and infectious diseases, DRS also offers a potential route for direct interrogation of pathogen and host transcriptomes, including RNA processing changes and candidate modification dynamics that may prove informative for diagnosis, disease monitoring, or therapeutic decision‐making [700, 701]. Beyond diagnostics, DRS is being explored as a possible platform for quality control of RNA therapeutics, where single‐molecule assessment of sequence integrity, poly(A) tail length, and engineered nucleotide content may be valuable for mRNA vaccines and RNA drugs. Taken together, these examples suggest translational potential, but routine clinical implementation will require rigorous analytical validation, standardized reporting, and demonstration of added value over existing assays.

### Toward broader deployment: Standardization and multiomics integration

Despite its potential, the widespread adoption of DRS remains constrained by cost, signal noise, and analytical complexity, particularly for modification inference [51]. A realistic path forward may be task‐specific standardization, with best‐practice workflows for transcript structure and APA analysis, poly(A) tail profiling, and RNA modification calling, each accompanied by explicit quality‐control metrics and computational expectations (Figure 10F). Community benchmarks and shared reference data sets will be essential for ensuring reproducibility across laboratories and application domains. In parallel, DRS may have the greatest impact when integrated with complementary omics rather than standalone use. Another promising direction is the implementation of simultaneous DNA/RNA sequencing within a unified workflow, enabling the concurrent profiling of genomic variation, epigenetic features, transcript isoforms, poly(A) tail dynamics, and RNA modifications from matched samples while minimizing batch effects. Such integrated native‐molecule sequencing approaches could provide a more direct framework for linking genotype, chromatin and DNA modification states, transcript processing, and posttranscriptional regulation within the same biological context. Positioning DRS as the native‐RNA layer alongside srRNA‐seq, high‐quality genome assemblies, chromatin accessibility assays, ribosome profiling, proteomics, and metabolomics has helped explain why transcript abundance alone does not always predict protein output and how post‐transcriptional regulation reshapes biological responses [346, 352, 702]. In biomedical contexts, similar integrative strategies may support emerging applications in liquid biopsy [418], infectious disease surveillance [703], and RNA therapeutic quality control [704], especially where preserving the native linkage between sequence, processing state, and modification is advantageous for interpretation. Realizing the broader value of DRS will require continued advances in protocol optimization, computational efficiency, benchmarking, and regulatory‐grade validation. As these barriers are progressively addressed, DRS may evolve from a powerful research technology into an increasingly important platform for mapping RNA regulation with greater molecular resolution.

## CONCLUSIONS

In summary, nanopore DRS has developed from a specialized tool for isoform discovery into a versatile, integrative platform capable of jointly profiling transcript structure, APA, RNA modifications, and haplotype context at the level of native RNA molecules. Across diverse biological systems, this unified molecular readout has enabled mechanistic insights that extend beyond descriptive transcript catalogues, revealing how multiple layers of RNA regulation are coordinated on individual transcripts. We present DRS not as a standalone solution to RNA biology, but as a molecule‐level integrative scaffold for interrogating native RNA features across transcript structure, tailing states, and chemical signals. Within this conceptual framework, we organize the field around three key questions: (i) which biological problems are best addressed by DRS, (ii) what levels of confidence can be assigned to different classes of conclusions, and (iii) which benchmarking and validation strategies are required to ensure robustness and reproducibility. Under this trajectory, DRS is likely to become an important layer for evolutionary transcriptomics and for the development of data‐driven models of RNA regulation across biological domains.

## AUTHORS CONTRIBUTIONS

**Tianyuan Zhang**: Conceptualization; project administration; supervision; data curation; writing—original draft; writing—review and editing. **Jia Li**: Data curation; writing—original draft; writing—review and editing. **Chao Tang**: Data curation; writing—original draft. **You Wu**: Data curation; writing—original draft. **Hao Wu**: Data curation; writing—original draft. **Xi‐Tong Zhu**: Data curation; writing—original draft. **Ziyang Luo**: Data curation; writing—original draft. **Hang Qin**: Data curation; writing—original draft. **Lishan Ding**: Data curation; writing—original draft; writing—review and editing. **Yu Zeng**: Investigation; writing—review and editing. **Shiou Yih Lee**: Investigation; writing—review and editing. **Xiaotao Shen**: Investigation; writing—review and editing. **Shiwen Gao**: Investigation; writing—review and editing. **Zhaoyang Tian**: Investigation; writing—review and editing. **Qian Tang**: Investigation; writing—review and editing. **Mian Li**: Investigation; writing—review and editing. **Muhammad Tahir Ul Qamar**: Investigation; writing—review and editing. **Yang Dong**: Investigation; writing—review and editing. **Komivi Dossa**: Investigation; writing—review and editing. **Yaxuan Zhang**: Investigation; writing—review and editing. **Hu Chen**: Conceptualization; resources; supervision; writing—review and editing. **Sanqi An**: Conceptualization; funding acquisition; writing—review and editing. **Xiang Yu**: Conceptualization; funding acquisition; writing—review and editing. **Lu Chen**: Conceptualization; funding acquisition; writing—review and editing. **Dingjie Wang**: Conceptualization; funding acquisition; writing—review and editing. **Shengli Li**: Funding acquisition; writing—review and editing. **Ling‐Ling Chen**: Conceptualization; funding acquisition; writing—review and editing. **Yanqiang Li**: Conceptualization; funding acquisition; writing—review and editing. All authors have read the final manuscript and approved it for publication.

## CONFLICT OF INTEREST STATEMENT

Tianyuan Zhang and Hu Chen are employees of Benagen Institute. Zhaoyang Tian is an employee of Sailgene Technology Co., Limited. The remaining authors declare no conflict of interest.

## ETHICS STATEMENT

No animals or humans were involved in this study.

## Supporting information


**Table S1:** Scenario‐oriented comparison of nanopore DRS and other RNA sequencing approaches.
**Table S2:** Practical comparison of major RNA sequencing and RNA modification detection approaches.
**Table S3:** Computational tools for RNA modification detection and analysis using Oxford Nanopore DRS.
**Table S4:** Computational tools for Oxford Nanopore DRS data analysis beyond RNA modification detection.
**Table S5:** Benchmarking resources and validation strategies for ONT DRS.

**Note S1:** Detailed experimental procedures for direct RNA sequencing of diverse RNA species.

## Data Availability

No new biological data were generated in this review. The source codes are available at https://zhangtianyuan666.github.io/DRS_doc and in the archived online repository at https://doi.org/10.24433/CO.3183037.v1. Supplementary materials (notes, tables, graphical abstract, slides, videos, Chinese translated version and update materials) may be found in the online DOI or iMeta Science http://www.imeta.science/.
