# Supplementary material for: Nanopore direct RNA sequencing and the epitranscriptome: Advances in mapping native RNA landscapes
Source: Imeta. 2026 Jul 1;5(3):e70136. doi: 10.1002/imt2.70136 (PMC13614359; doi:10.1002/imt2.70136)
Supplement: Supplementary file 1 [file IMT2-5-e70136-s001.docx]

Supporting Information to

**Nanopore direct RNA sequencing and the epitranscriptome: Advances in mapping native RNA landscapes**

**Running title:** An encyclopedic guide to nanopore DRS

Tianyuan Zhang^1, 2#, *^, Jia Li^3#^, Chao Tang^4, 5#^, You Wu^6#^, Hao Wu^7#^, Xi-Tong Zhu^8#^, Ziyang Luo^9#^, Hang Qin^1#^, Lishan Ding^10, 11#^, Yu Zeng^7^, Shiou Yih Lee^12^, Xiaotao Shen^13, 14^, Shiwen Gao^3^, Zhaoyang Tian^15^, Qian Tang^8^, Mian Li^16^, Muhammad Tahir Ul Qamar^17^, Yang Dong^1^, Komivi Dossa^18, 19^, Yaxuan Zhang^1^, Hu Chen^2*^, Sanqi An^9*^, Xiang Yu^6*^, Lu Chen^4*^, Dingjie Wang^3, 20*^, Shengli Li^7*^, Ling-Ling Chen^8, 21*^, Yanqiang Li^1*^

^1^Department of Urology, The First Affiliated Hospital of Xi’an Jiaotong University, Xi’an 710061, China

^2^Benagen Institute, Wuhan 430073, China

^3^School of Mathematics and Statistics, Wuhan University, Wuhan 430072, China

^4^Department of Laboratory Medicine, West China Second University Hospital, Key Laboratory of Birth Defects and Related Diseases of Women and Children, Ministry of Education, State Key Laboratory of Biotherapy, Sichuan University, Chengdu 610041, China

^5^Institute of Basic Medicine, School of Basic Medical Sciences and Forensic Medicine, North Sichuan Medical College, Nanchong 637000, China

^6^School of Life Sciences and Biotechnology, Shanghai Jiao Tong University, Shanghai 200240, China

^7^Precision Research Center for Refractory Diseases, Shanghai Jiao Tong University Pioneer Research Institute for Molecular and Cell Therapies, Shanghai General Hospital, Shanghai Jiao Tong University School of Medicine, Shanghai 201620, China

^8^College of Life Science and Technology, Guangxi University, Nanning 530004, China

^9^Institute of Transplant Medicine, The Second Affiliated Hospital of Guangxi Medical University, Guangxi Clinical Research Center for Organ Transplantation, Guangxi Key Laboratory of Organ Donation and Transplantation, Guangxi Key Laboratory of AIDS Prevention and Treatment, Guangxi Medical University, Nanning 530007, China

^10^School of Pharmacy, Chengdu University of Traditional Chinese Medicine, Chengdu 611137, China

^11^Institute of Herbgenomics, Chengdu University of Traditional Chinese Medicine, Chengdu 611137, China

^12^Health and Life Sciences, INTI International University, Negeri Sembilan 71800, Malaysia

^13^Lee Kong Chian School of Medicine, Nanyang Technological University, Singapore 636921, Singapore

^14^School of Chemistry, Chemical Bioneering, and Biotechnology, Nanyang Technological University, Singapore 637371, Singapore

^15^Sailgene Technology Co., Limited, Hong Kong 999077, China

^16^Biomedical Sciences College & Shandong Medicinal Biotechnology Centre, Shandong First Medical University & Shandong Academy of Medical Sciences, Jinan 250000, China

^17^Department of Bioinformatics and Biotechnology, Government College University, Faisalabad 38000, Pakistan

^18^CIRAD, UMR AGAP Institut, Petit Bourg, Guadeloupe 97170, France
^19^UMR AGAP Institut, CIRAD, INRAE, Univ Montpellier, Institut Agro, Montpellier 34398, France

^20^Institute for Math & AI, Wuhan University, Wuhan 430072, China

^21^Yazhouwan National Laboratory, Sanya 572025, China

^#^These authors contributed equally: Tianyuan Zhang, Jia Li, Chao Tang, You Wu, Hao Wu, Xi-Tong Zhu, Ziyang Luo, Hang Qin, Lishan Ding

^*^Correspondence: [zhangtianyuan@foxmail.com](mailto:zhangtianyuan@foxmail.com) (Tianyuan Zhang); [chenhu@benagen.com](mailto:chenhu@benagen.com) (Hu Chen); 1721967953@qq.com (Sanqi An); yuxiang2021@sjtu.edu.cn (Xiang Yu); luchen@scu.edu.cn (Lu Chen); djwang@whu.edu.cn (Dingjie Wang); shengli.li@sjtu.edu.cn (Shengli Li); chenlingling@yzwlab.cn (Ling-Ling Chen); [yanqiang.li@xjtu.edu.cn](mailto:yanqiang.li@xjtu.edu.cn) (Yanqiang Li).

**Supplemental Note 1 Detailed Experimental Procedures for Direct RNA Sequencing of Diverse RNA Species**

**SUMMARY**

This supplemental note describes complete step-by-step experimental protocols for direct RNA sequencing library preparation of six types of RNA molecules, including mRNA, rRNA, tRNA, circular RNA (circRNA), microRNA (miRNA) and non-poly(A) RNA, all based on the Oxford Nanopore Technologies (ONT) sequencing platform. The protocols cover the full workflow from total RNA extraction, RNA enrichment or pretreatment, library construction, flow cell priming and loading, to sequencing parameter configuration and core data processing procedures. For each RNA type, we provide targeted pretreatment strategies (e.g., polyadenylation for rRNA, splint adapter ligation for tRNA, RNase R treatment for circRNA) to overcome the limitations of conventional direct RNA sequencing workflows, and specify critical quality control (QC) checkpoints at each experimental stage to ensure library quality and sequencing stability.

**KEY RESOURCES TABLE**

| **REAGENT or RESOURCE** | **SOURCE** | **IDENTIFIER** |
| --- | --- | --- |
| **Recombinant enzymes** |  |  |
| Murine RNase Inhibitor | New England Biolabs (NEB), Ipswich, MA, USA | M0314 |
| T4 DNA Ligase | NEB | M0202 |
| Induro Reverse Transcriptase | NEB | M0681 |
| E. coli Poly(A) Polymerase | NEB | M0276 |
| Maxima H Minus Reverse Transcriptase | Thermo Fisher Scientific, Waltham, MA, USA | EP0751 |
| T4 RNA Ligase 2 | NEB | M0239 |
| Turbo DNase | Thermo Fisher Scientific | AM2238 |
| RNase R | NEB | M0100S |
| T4 Polynucleotide Kinase (PNK) | NEB | M0201V |
| **Kits and columns** |  |  |
| Agencourt RNAClean XP Beads | Beckman Coulter, Brea, CA, USA | A63987/A66514 |
| Qubit dsDNA HS Assay Kit | Thermo Fisher Scientific | Q32851 |
| Qubit RNA HS Assay Kit | Thermo Fisher Scientific | Q32852 |
| Monarch® Spin RNA Cleanup Kit | NEB | #T2050 |
| Zymo RNA Clean and Concentrator-5 kit | Zymo Research, Irvine, CA, USA | R1013 |
| RNeasy MinElute Cleanup Kit | Qiagen, Hilden, Germany | 74204 |
| RiboMinus Eukaryote System V2 | Thermo Fisher Scientific | A15020/A15026 |
| miRNeasy Mini Kit | Qiagen | 217004 |
| ONT Direct RNA Sequencing Kit (SQK-RNA002) | Oxford Nanopore Technologies (ONT), Oxford, UK | SQK-RNA002 |
| ONT Direct RNA Sequencing Kit (SQK-RNA004) | ONT | SQK-RNA002 |
| P3 Primary Cell 4D-Nucleofector X Kit | Lonza, Basel, Switzerland | V4XP-3024 |
| **Chemicals and buffers** |  |  |
| NEBNext Quick Ligation Reaction Buffer | NEB | B6058S |
| Nuclease-free water | - | - |
| 70% ethanol (freshly prepared) | - | - |
| Wash Buffer (WSB) | ONT | SQK-RNA004 |
| RNA Elution Buffer (REB) | ONT | SQK-RNA004 |
| Library Solution (LIS) | ONT | SQK-RNA004 |
| RNA Flush Tether (RFT) | ONT | SQK-RNA004 |
| RNase-free resuspension buffer (RRB) | ONT | SQK-RNA004 |
| TRIzol Reagent | Thermo Fisher Scientific | 15596018 |
| Acid-washed glass beads (425–600 µm) | Sigma-Aldrich, St. Louis, MO, USA | G8772 |
| Chloroform | Sigma | C2432 |
| Isopropanol | Sigma | I9516 |
| GlycoBlue co-precipitant | Thermo Fisher Scientific | AM9515 |
| 100 mM Tris-HCl (pH 9.0) | - | - |
| 10 mM Tris-HCl (pH 7.5) | - | - |
| 50 mM NaCl | - | - |
| 20% PEG 8000 | - | - |
| 1× T4 RNA Ligase 2 Buffer | NEB | M0239 |
| 10× *Escherichia*  *coli* Poly(A) Polymerase Reaction Buffer | NEB | M0276 |
| 10 mM ATP | NEB | P0756S |
| EDTA | Sigma | E9884 |
| Betaine | Sigma | B0300 |
| 10 mM dNTPs | NEB | N0447S/N0447L |
| 5× Maxima H Minus Reverse Transcriptase Buffer | Thermo Fisher Scientific | EP0751/EP0752/EP0753 |
| **Oligonucleotides** |  |  |
| RT Adapter (RTA) | ONT | SQK-RNA004 |
| RNA Ligation Adapter (RLA) | ONT | SQK-RNA004 |
| RNA CS | ONT | SQK-RNA004 |
| U6-Fwd sequencing primer | - | 5′ GACTATCATATGCTTACCGT 3′ |
| 5′ splint adapter for tRNA | - | 5′ rCrCrUrArArGrArGrCrArArGrArArGrArArGrCrCrUrGrGrN 3′ |
| 3′ splint adapter (RNA:DNA hybrid) for tRNA | - | 5′ /5Phos/rGrGrCrUrCrUrCrUrCrUrGrCrUrCrUrArGrGrArArArArArArArArArArArArArArArArArArArArA + 3′ poly(A) DNA tail |
| ONT RTA oligo A | ONT | SQK-RNA004 |
| ONT RTA oligo B | ONT | SQK-RNA004 |
| RMX sequencing adapters | ONT | SQK-RNA002/004 |
| **Experimental models and samples** |  |  |
| Total RNA extraction kit for animal tissues | Benagen, Wuhan, China | RT113 |
| Total RNA extraction kit for plant tissues | Benagen | RS111 |
| **Instrument and software** |  |  |
| Agilent 2100 TapeStation/Bioanalyzer | Agilent Technologies, Santa Clara, CA, USA | G2939BA |
| Qubit 3.0/4.0 Fluorometer | Thermo Fisher Scientific | Q33216 for Qubit 3.0; Q33238 for Qubit 4 Fluorometer |
| 0.2 mL PCR tubes | Axygen/Corning, Corning, NY, USA | PCR-02-C |
| 1.5 mL microcentrifuge tubes | Axygen | MCT-150-C |
| Magnetic rack | Thermo Fisher Scientific | 12321D |
| Rotator mixer | - | - |
| Thermal cycler | Bio-Rad Laboratories, Hercules, CA, USA | T100 Thermal Cycler, 1861096 |
| Fluorescent Viability Cell Counter | Nexcelom Bioscience, Lawrence, MA, USA | K2 |
| Water bath | Fisher Scientific Waltham, MA, USA | Isotemp general-purpose water bath |
| Agarose gel electrophoresis equipment | Apogee Designs, USA | - |
| ONT MinION/GridION/PromethION Sequencer | ONT | - |
| RNA flow cell (FLO-MIN004RA/FLO-PRO004RA) | ONT | - |
| 4D-Nucleofector X Unit | Lonza, Basel, Switzerland | AAF-1002X |
| NanoDrop spectrophotometer | Thermo Fisher Scientific | NanoDrop One |
| MinKNOW Software | ONT | - |
| Dorado Basecaller | ONT | - |
| BWA-MEM | https://github.com/lh3/bwa | - |
| minimap2 | https://github.com/lh3/minimap2 | - |
| DESeq2 | Bioconductor | - |
| CRISPResso2 | https://github.com/pinellolab/CRISPResso2 | [-](https://crispresso.pinellolab.partners.org/submission) |
| CIRI-long | https://github.com/bioinfo-biols/CIRI-long | - |
| CIRCexplorer | https://github.com/YangLab/CIRCexplorer2 | - |
| NanoRMS | https://github.com/novoalab/nanoRMS | - |
| xPore | https://github.com/GoekeLab/xpore | - |
| Nanocompore | https://github.com/tleonardi/nanocompore | - |
| ELIGOS | https://gitlab.com/piroonj/eligos2 | - |
| CircRM | https://github.com/jiayiAnnie17/CircRM | - |

**-：indicates that no catalog number is specified for reagents, or no version number is specified for software.**

**BEFORE YOU BEGIN**

1. **RNA quality requirements**

All RNA samples used for direct RNA sequencing library preparation must meet the following quality criteria to ensure sequencing results:

- For mRNA, rRNA, circRNA and non-poly(A) RNA sequencing: RNA Integrity Number (RIN) ≥ 8.0, with RIN ≥ 8.5 recommended;
- For tRNA and miRNA sequencing: RNA samples must be free of severe degradation, with clear enrichment of target small RNA fragments verified by TapeStation/Bioanalyzer;
- RNA purity: A260/280 ratio between 1.9 and 2.2, A260/230 ratio ≥ 2.0, verified by NanoDrop spectrophotometer.

2. **Reagent and consumable preparation**

- All reagents must be nuclease-free, and all consumables (PCR tubes, pipette tips, etc.) must be RNase-free to avoid RNA degradation;
- Prepare 70% ethanol fresh on the day of the experiment using nuclease-free water and absolute ethanol;
- Thaw all enzyme reagents on ice before use, mix gently by flicking the tube, and perform a brief spin-down; do not vortex enzymes to avoid loss of activity.

3. **Instrument pre-inspection**

- Verify that the thermal cycler, magnetic rack, Qubit fluorometer, and Agilent TapeStation/Bioanalyzer are functioning properly and calibrated;
- For ONT sequencing, perform a flow cell check to assess pore count before library loading, and ensure that the MinKNOW software is correctly configured.

4. **Institutional permissions**

All experiments involving human/animal samples must comply with relevant ethical regulations and obtain approval from the institutional ethics committee.

5. **Critical precautions for RNA handling**

To preserve native RNA modification sites and secondary/tertiary structures, avoid thermal denaturation, vigorous vortexing, and repeated freeze-thaw cycles of RNA samples throughout the experimental process.

**STEP-BY-STEP METHOD DETAILS**

**Protocol 1 mRNA Direct RNA Sequencing Library Preparation**

**Timing**: 4–5 h

This protocol describes the complete workflow for mRNA direct RNA sequencing library construction, including reverse transcription adapter ligation, reverse transcription, bead purification, sequencing adapter ligation, final library purification, and flow cell priming and loading.

**1.1 Starting Material Preparation**

**Timing**: 10 min

a. Input requirements: 200 ng poly(A) RNA or 2.0 μg total RNA.

b. Adjust the RNA sample to a final volume of 8 μL with nuclease-free water in a 0.2 mL PCR tube.

**1.2 RT-Adapter Ligation**

**Timing**: 10–20 min

a. Prepare the ligation reaction mix in the 0.2 mL PCR tube containing the RNA sample according to Supplemental Table 1.

b. Mix gently by pipetting, and incubate the reaction at room temperature for 10–20 minutes.

Supplemental Table 1 RT-Adapter Ligation Reaction System

| **Component** | **Volume** |
| --- | --- |
| RNA sample | 8 μL |
| NEBNext Quick Ligation Reaction Buffer | 3 μL |
| RNA CS (or nuclease-free water) | 0.5 μL |
| Murine RNase Inhibitor | 1 μL |
| RT Adapter (RTA) | 1 μL |
| T4 DNA Ligase | 1.5 μL |
| **Total volume** | **15 μL** |

**1.3 Reverse Transcription**

**Timing**: 40–70 min

a. Prepare the reverse transcription reaction mix according to Supplemental Table 2, and add it directly to the ligation reaction from Step 1.2.

b. Mix gently by pipetting, and perform the reaction in a thermal cycler with the following program: 60°C for 30–60 minutes, 70°C for 10 minutes, then hold at 4°C.

Supplemental Table 2 Reverse Transcription Reaction System

| Component | Volume |
| --- | --- |
| Nuclease-free water | 13 μL |
| Induro Reverse Transcriptase | 2 μL |
| **Total volume added** | **15 μL** |
| **Final reaction volume** | **30 μL** |

**1.4 Purification with Agencourt RNAClean XP Beads**

**Timing**: 20–25 min

a. Add 72 μL of Agencourt RNAClean XP Beads to the reverse transcription product, mix thoroughly by pipetting.

b. Rotate the tube at room temperature for 5 minutes on a rotator mixer.

c. Place the tube on a magnetic rack to separate the beads, and discard the supernatant after the solution is clear.

d. Wash the beads twice with 150 μL of freshly prepared 70% ethanol, keeping the tube on the magnetic rack during washing.

e. Air-dry the beads at room temperature until the ethanol is completely evaporated.

f. Elute the purified product with 23 μL nuclease-free water, and retain the full 23 μL of eluate for the next step.

**1.5 RNA Ligation Adapter Ligation**

**Timing**: 10 min

a. Prepare the ligation reaction mix according to Supplemental Table 3, and add it to the 23 μL eluate from Step 1.4.

b. Mix gently by pipetting, and incubate the reaction at room temperature for 10 minutes.

Supplemental Table 3 RNA Ligation Adapter Ligation Reaction System

| **Component** | **Volume** |
| --- | --- |
| Purified sample from Step 1.4 | 23 μL |
| NEBNext Quick Ligation Reaction Buffer | 8 μL |
| RNA Ligation Adapter (RLA) | 6 μL |
| T4 DNA Ligase | 3 μL |
| **Total volume** | **40 μL** |

**1.6 Second Purification and Elution**

**Timing**: 20–25 min

a. Add 16 μL of Agencourt RNAClean XP Beads to the ligation product from Step 1.5, mix thoroughly by pipetting.

b. Rotate the tube at room temperature for 5 minutes on a rotator mixer.

c. Place the tube on a magnetic rack to separate the beads, and discard the supernatant after the solution is clear.

d. Wash the beads twice with 150 μL Wash Buffer (WSB), keeping the tube on the magnetic rack during washing.

e. Air-dry the beads at room temperature briefly.

f. Elute the final library with 13 μL RNA Elution Buffer (REB), and retain the full 13 μL of eluate for quantification.

**1.7 Library Quality Control**

**Timing**: 10 min

a. Quantify the final library using the Qubit dsDNA HS assay.

b. **Note**: Target library recovery >30 ng for subsequent sequencing.

**1.8 Flow Cell Priming and Loading**

**Timing**: 30–40 min

This section describes the flow cell preparation, priming, and library loading procedures for ONT direct RNA sequencing.

**1.8.1 Flow Cell Preparation**

a. Remove the RNA flow cell (FLO-MIN004RA/FLO-PRO004RA) from the refrigerator, and leave it at room temperature for 20 minutes to equilibrate.

b. Prepare the priming mix: RNA Flush Tether (RFT) 30 μL.

c. Open the priming port of the flow cell, and gently withdraw 20–30 μL of buffer to remove air bubbles from the flow cell channel.

d. Load 800 μL of the priming mix into the flow cell via the priming port, close the port, and wait for 5 minutes.

**1.8.2 Library Loading**

a. In a 1.5 mL tube, prepare the library for loading: mix 75 μL of the final library with 25 μL Library Solution (LIS) if applicable, to a final volume of 100 μL.

b. Open the SpotON sample port of the flow cell.

c. Load an additional 200 μL of priming mix into the priming port.

d. Add the prepared library dropwise into the SpotON sample port, ensuring the library enters the flow cell channel correctly.

e. Close the SpotON cover and priming port, install the light shield, and start the sequencing run in the MinKNOW software.

**Protocol 2 rRNA Direct RNA Sequencing Library Preparation (Based on SQK-RNA004)**

**Timing**: 6–8 h

This protocol describes the complete workflow for rRNA direct RNA sequencing library construction, including total RNA extraction, rRNA enrichment, library construction, sequencing parameter optimization, and data applications.

**2.1 Total RNA Extraction and Quality Assessment**

**Timing**: 1–2 h

a. Extract total RNA from samples using a standard RNA extraction method.

b. Assess RNA integrity using an Agilent 2100 TapeStation or equivalent system.

c. **CRITICAL**: Only RNA samples with RIN ≥ 8.5 are acceptable for library preparation.

d. **CRITICAL**: To preserve native modification sites and secondary/tertiary structures of rRNA, avoid thermal denaturation or vigorous vortexing of RNA samples throughout the experimental process.

**2.2 rRNA Enrichment Strategy (Choose One of the Two Options)**

**Timing**: 1.5–2 h

**Option 1: In Vitro Polyadenylation**

This strategy adds a poly(A) tail to the 3′ end of rRNA, enabling compatibility with conventional mRNA library adapter systems.

a. Gently vortex the 10× reaction buffer and 10 mM ATP, and gently flick the *E. coli* Poly(A) Polymerase tube to mix, followed by a brief spin-down.

b. Prepare a 20 µL polyadenylation reaction mixture in a 0.2 mL thin-walled PCR tube according to Supplemental Table 4.

c. Incubate the reaction at 37 °C for 30 minutes.

d. Stop the reaction by adding EDTA to a final concentration of 10 mM. Alternatively, directly proceed to cleanup using a column-based RNA cleanup kit (e.g., Monarch® Spin RNA Cleanup Kit, NEB #T2050).

e. Elute the polyadenylated RNA in nuclease-free water, and quantify using a Qubit fluorometer or equivalent instrument.

Supplemental Table 4 In Vitro Polyadenylation Reaction System

| **Component** | **Volume** |
| --- | --- |
| Nuclease-free water | Variable (adjust to final 20 µL) |
| Total RNA (1–10 µg) | Variable |
| 10× E. coli Poly(A) Polymerase Reaction Buffer | 2 µL |
| 10 mM ATP | 2 µL |
| E. coli Poly(A) Polymerase (5 units) | 1 µL |
| Murine RNase Inhibitor (20 units, optional) | 0.5 µL |
| **Total volume** | **20 µL** |

**Option 2: Specific Reverse Transcription Adapter (RTA) Ligation**

Design RTAs complementary to the 3′ termini of rRNA, enabling specific recognition and ligation without polyadenylation.

**2.3 Library Construction (Based on SQK-RNA004 Workflow)**

**Timing**: 3.5–4 h

**2.3.1 RT-Adapter Ligation**

**Timing**: 10 min

a. Prepare the ligation reaction mix in a 0.2 mL PCR tube according to Supplemental Table 5.

b. Mix gently by pipetting, and incubate at room temperature for 10 minutes.

Supplemental Table 5 RT-Adapter Ligation Reaction System for rRNA

| **Component** | **Volume** |
| --- | --- |
| Total RNA (200 ng poly(A) RNA or 2 μg total RNA) | 8 μL |
| NEBNext Quick Ligation Reaction Buffer | 3 μL |
| RNA CS (or nuclease-free water) | 0.5 μL |
| Murine RNase Inhibitor | 1 μL |
| RT Adapter (RTA) | 1 μL |
| T4 DNA Ligase | 1.5 μL |
| **Total volume** | **15 μL** |

**2.3.2 Reverse Transcription (with Structural Unfolding Enhancement)**

**Timing**: 40 min

a. Prepare the reverse transcription reaction mix according to Supplemental Table 6, and add it directly to the ligation reaction from Step 3.1.

b. **Note**: Betaine may be optionally added to a final concentration of 1 M to enhance the ability of reverse transcriptase to traverse structured regions of rRNA.

c. Mix gently by pipetting, and perform the reaction in a thermal cycler with the following program: 60°C for 30 minutes, 70°C for 10 minutes, then hold at 4°C.

Supplemental Table 6 Reverse Transcription Reaction System for rRNA

| Component | Volume |
| --- | --- |
| Nuclease-free water | 13 μL |
| Induro Reverse Transcriptase | 2 μL |
| **Total volume added** | **15 μL** |
| **Final reaction volume** | **30 μL** |

**2.3.3 Purification with Agencourt RNAClean XP Beads**

**Timing**: 20–25 min

a. Add 72 μL of Agencourt RNAClean XP Beads to the reverse transcription product, mix thoroughly by pipetting.

b. Rotate the tube at room temperature for 5 minutes on a rotator mixer.

c. Place the tube on a magnetic rack to separate the beads, and discard the supernatant after the solution is clear.

d. Wash the beads twice with 150 μL of freshly prepared 70% ethanol, keeping the tube on the magnetic rack during washing.

e. Air-dry the beads at room temperature until the ethanol is completely evaporated.

f. Elute the purified product with 23 μL nuclease-free water, and retain the full 23 μL of eluate for the next step.

**2.3.4 RNA Ligation Adapter Ligation**

**Timing**: 10 min

a. Prepare the ligation reaction mix according to Supplemental Table 7, and add it to the 23 μL eluate from Step 3.3.

b. Mix gently by pipetting, and incubate the reaction at room temperature for 10 minutes.

Supplemental Table 7 RNA Ligation Adapter Ligation Reaction System for rRNA

| **Component** | **Volume** |
| --- | --- |
| Purified sample from Step 3.3 | 23 μL |
| NEBNext Quick Ligation Reaction Buffer | 8 μL |
| RNA Ligation Adapter (RLA) | 6 μL |
| T4 DNA Ligase | 3 μL |
| **Total volume** | **40 μL** |

**2.3.5 Second Purification and Elution**

**Timing**: 20–25 min

a. Add 16 μL of Agencourt RNAClean XP Beads to the ligation product from Step 3.4, mix thoroughly by pipetting.

b. Rotate the tube at room temperature for 5 minutes on a rotator mixer.

c. Place the tube on a magnetic rack to separate the beads, and discard the supernatant after the solution is clear.

d. Wash the beads twice with 150 μL Wash Buffer (WSB), keeping the tube on the magnetic rack during washing.

e. Air-dry the beads at room temperature briefly.

f. Elute the final library with 13 μL RNA Elution Buffer (REB), and retain the full 13 μL of eluate for quantification.

**2.3.6 Library Quality Control**

**Timing**: 10 min

a. Quantify the final library using the Qubit dsDNA HS assay.

b. **Note**: Target library recovery >30 ng for subsequent sequencing.

**2.4 Sequencing Parameter Optimization (for Long rRNA Fragments)**

**Timing**: 20 min

a. Use an RNA-specific flow cell (e.g., FLO-MIN004RA/FLO-PRO004RA) for sequencing.

b. Install the light shield after library loading to minimize photobleaching and maintain pore stability during the sequencing run.

**2.5 Data Applications**

The rRNA direct RNA sequencing data generated by this protocol can be used for the following applications:

a. Detection of diverse modifications in bacterial and eukaryotic rRNAs (e.g., m⁷G, Ψ);

b. Characterization of dynamic changes in rRNA modification patterns under stress conditions.

**Protocol 3 Direct tRNA Sequencing Library Preparation**

**Timing**: 2–3 days

This protocol describes the complete workflow for direct tRNA sequencing library construction, including total RNA extraction, small RNA enrichment, tRNA deacylation, splint adapter design and ligation, reverse transcription, final library preparation, sequencing, and data processing.

**3.1 Total RNA Extraction and Small RNA Enrichment**

**Timing**: 3–4 h

**3.1.1 Total RNA Extraction**

a. Extract total RNA from cell pellets using TRIzol Reagent. Resuspend cell pellets in 660 µL TRIzol with 340 µL acid-washed glass beads (425–600 µm).

b. Disrupt cells by vortexing for seven cycles of 15 seconds each, with 30-second ice incubations between cycles.

c. Add 200 µL chloroform, vortex briefly, incubate at room temperature for 5 minutes, and centrifuge at 14,000 × g for 15 minutes at 4°C.

d. Transfer the upper aqueous phase to a new tube. Add 1 volume of isopropanol and 1 µL GlycoBlue co-precipitant, mix thoroughly, and incubate at room temperature for 10 minutes.

e. Centrifuge at 14,000 × g for 15 minutes at 4°C. Wash the pellet with ice-cold 70% ethanol, air-dry, and resuspend in nuclease-free water.

f. Treat the RNA sample with Turbo DNase according to the manufacturer's instructions to remove genomic DNA contamination.

**3.1.2 Small RNA Enrichment (17–200 nt)**

**Timing**: 1.5–2 h

a. Use the Zymo RNA Clean and Concentrator-5 kit for small RNA enrichment. Adjust the RNA sample to a volume of 50 µL with nuclease-free water.

b. Add 1 volume of RNA Binding Buffer and 1 volume of 100% ethanol to the RNA sample, and mix thoroughly.

c. Transfer the mixture to a Zymo-Spin IC column and centrifuge at ≥ 12,000 × g for 1 minute. Retain the flow-through, which contains small RNAs (17–200 nt).

d. Add 1 volume of 100% ethanol to the flow-through, mix thoroughly, and transfer to a new Zymo-Spin IC column. Centrifuge at ≥ 12,000 × g for 1 minute.

e. Add 400 µL RNA Prep Buffer to the column and centrifuge, discard the flow-through.

f. Add 800 µL RNA Wash Buffer to the column and centrifuge for 2 minutes. Transfer the column to a fresh collection tube and centrifuge for 1 minute to remove residual buffer.

g. Elute small RNA in 11 µL nuclease-free water. Quantify using the Qubit RNA HS Assay, and assess RNA quality using TapeStation or Bioanalyzer.

**3.2 tRNA Deacylation**

**Timing**: 1 h

a. Adjust the small RNA fraction to 10 µL with nuclease-free water.

b. Add 90 µL of 100 mM Tris-HCl (pH 9.0) to the sample, mix gently.

c. Incubate the reaction at 37 °C for 30 minutes to remove aminoacyl groups from tRNA.

d. Purify the deacylated RNA using the Zymo RNA Clean and Concentrator-5 kit as described in Step 1.2, with an increased ethanol concentration to 1.3× after the RNA Prep Buffer step.

e. Elute the deacylated tRNA in 8 µL nuclease-free water, and quantify using the Qubit RNA HS Assay.

f. **Note**: A minimum of 250 ng deacylated tRNA is recommended for subsequent library construction.

**3.3 Splint Adapter Design and Annealing**

**Timing**: 30 min

**3.3.1 Adapter Design**

The following splint adapters are used for tRNA library construction:

- 5′ splint adapter: Complementary to the 3′ CCA overhang of mature tRNAs. Sequence: 5′ rCrCrUrArArGrArGrCrArArGrArArGrArArGrCrCrUrGrGrN 3′
- 3′ splint adapter (RNA:DNA hybrid): Contains a 5′ phosphate and a 3′ poly(A) segment for RTA adapter binding. Sequence: 5′ /5Phos/rGrGrCrUrCrUrCrUrCrUrGrCrUrCrUrArGrGrArArArArArArArArArArArArArArArArArArArArA + 3′ poly(A) DNA tail

**3.3.2 Adapter Annealing**

a. Prepare the annealing mix in a 0.2 mL PCR tube: 5′ and 3′ splint adapters at a 1:1 molar ratio, 10 mM Tris-HCl (pH 7.5), 50 mM NaCl, 1 µL RNase Inhibitor (final concentration 50 ng/µL).

b. Perform the annealing reaction in a thermal cycler with the following program: heat to 75°C for 15 seconds, then cool to 25°C at a rate of 0.1°C per second.

c. Store the annealed adapters on ice until use.

**3.4 Splint Adapter Ligation to tRNA**

**Timing**: 2.5 h (overnight incubation optional)

a. Combine deacylated tRNA (250–500 ng) with pre-annealed splint adapters at a molar ratio of 1.2:1 (assuming average tRNA length of 90 nt) in a 0.2 mL PCR tube.

b. Prepare the ligation reaction mix according to Supplemental Table 8, to a total volume of 50 µL.

c. Mix gently by pipetting, and incubate the reaction at room temperature for 2 hours.

d. Add 2 volumes of room-temperature-equilibrated AMPure RNAClean XP beads to the ligation product, mix gently.

e. Incubate the tube on a rotator mixer for 15 minutes at room temperature.

f. Pellet the beads on a magnetic rack, wash twice with freshly prepared 70% ethanol, and air-dry briefly.

g. Elute the ligated product in nuclease-free water, and quantify using the Qubit RNA HS Assay.

Supplemental Table 8 Splint Adapter Ligation Reaction System for tRNA

| Component | Volume |
| --- | --- |
| Deacylated tRNA + pre-annealed splint adapters | Variable |
| 20% PEG 8000 | Variable (final 20%) |
| 1× T4 RNA Ligase 2 Buffer | Variable (final 1×) |
| Recombinant T4 RNA Ligase 2 (6 mg/mL) | 4 µL |
| RNase Inhibitor | 1 µL |
| **Total volume** | **50 µL** |

**3.5 Reverse Transcription Adapter (RTA) Ligation and Reverse Transcription**

**Timing**: 2–2.5 h

**3.5.1 RTA Adapter Annealing**

a. Anneal ONT RTA oligo A and oligo B (or custom DNA oligos with identical sequences) at a 1:1 molar ratio using the same annealing conditions as in Step 3.2.

**3.5.2 RTA Ligation**

**Timing**: 30 min

a. Combine 200 ng of splint-ligated tRNA with pre-annealed RTA adapter at a 1:2 molar ratio in a 0.2 mL PCR tube.

b. Prepare the ligation reaction mix according to Supplemental Table 9, to a total volume of 15 µL.

c. Mix gently by pipetting, and incubate the reaction at room temperature for 30 minutes.

Supplemental Table 9 RTA Ligation Reaction System for tRNA

| Component | Volume |
| --- | --- |
| Splint-ligated tRNA + pre-annealed RTA adapter | Variable |
| 1× Quick Ligation Reaction Buffer | Variable (final 1×) |
| T4 DNA Ligase | 1.5 µL |
| RNase Inhibitor | 0.5 µL |
| **Total volume** | **15 µL** |

**3.5.3 Reverse Transcription**

**Timing**: 1 h 10 min

a. Prepare the reverse transcription master mix per sample according to Supplemental Table 10.

b. Add the master mix directly to the RTA ligation reaction from Step 5.2, and mix gently by pipetting.

c. Perform the reaction in a thermal cycler with the following program: 60°C for 1 hour, 85°C for 5 minutes, then hold at 4°C.

d. Purify the linearized tRNA using 2 volumes of AMPure RNAClean XP beads as described in Step 4, eluting in 8.5 µL nuclease-free water.

Supplemental Table 10 Reverse Transcription Master Mix for tRNA

| **Component** | **Volume per reaction** |
| --- | --- |
| Nuclease-free water | 13 µL |
| 10 mM dNTPs | 2 µL |
| 5× Maxima H Minus Reverse Transcriptase Buffer | 8 µL |
| Maxima H Minus Reverse Transcriptase | 2 µL |
| **Total volume** | **25 µL** |

**3.6 Sequencing Adapter Ligation and Final Library Preparation**

**Timing**: 1.5 h

a. Combine the purified linearized tRNA from Step 5.3 with the reaction components according to Supplemental Table 11, to a total volume of 40 µL.

b. Mix gently by pipetting, and incubate the reaction at room temperature for 30 minutes.

c. Add 2 volumes of AMPure RNAClean XP beads to the reaction product, mix thoroughly.

d. Incubate the tube on a rotator mixer for 10 minutes at room temperature.

e. Pellet the beads on a magnetic rack, wash twice with 150 µL Wash Buffer (WSB), resuspending the beads by flicking the tube each time.

f. Elute the final library in 13 µL RNA Elution Buffer (REB), and incubate at room temperature for 10 minutes.

g. Transfer the eluate to a clean tube. Prepare the final library for loading by adding nuclease-free water and RNase-free resuspension buffer (RRB) according to the ONT protocol.

Supplemental Table 11 Sequencing Adapter Ligation Reaction System for tRNA

| Component | Volume |
| --- | --- |
| Purified linearized tRNA | Variable |
| 1× Quick Ligation Reaction Buffer | Variable (final 1×) |
| T4 DNA Ligase | 3 µL |
| RMX sequencing adapters (from ONT SQK-RNA002 kit) | 6 µL |
| **Total volume** | **40 µL** |

**3.7 Flow Cell Priming and Sequencing**

**Timing**: 30–40 min

**3.7.1 Flow Cell Preparation**

a. Use an RNA-specific flow cell (FLO-MIN004RA/FLO-PRO004RA). Allow the flow cell to equilibrate at room temperature for 20 minutes.

b. Perform a flow cell check to assess pore count.

c. Prepare the priming mix according to the ONT Direct RNA Sequencing Kit protocol.

d. Load the priming mix via the priming port, and wait 5 minutes.

**3.7.2 Library Loading**

a. Mix the final library gently by pipetting.

b. Load the library dropwise into the SpotON sample port.

c. Install the light shield and close the device lid.

**3.7.3 MinKNOW Configuration for tRNA Sequencing**

Configure the MinKNOW software with the following settings:

- Flow cell type: FLO-MIN004RA/FLO-PRO004RA
- Kit selection: Direct RNA Sequencing Kit (SQK-RNA004)
- Disable live basecalling to preserve raw signal data
- Enable saving of .POD5 file format
- Custom read classification settings to capture short reads

**3.8 Data Processing and Analysis**

**Timing**: Variable (depending on sequencing data volume)

**3.8.1 Basecalling and Read Filtering**

a. Basecall raw .POD5 files using Dorado with high-accuracy (hac) mode.

b. Convert all uracils (U) to thymines (T) in the output FASTQ files.

**3.8.2 Read Alignment**

a. Align reads using BWA-MEM with optimized parameters: bwa mem -W 13 -k 6 -x ont2d -T 20

b. Include the sequences of the 5′ and 3′ RNA adapters in the reference FASTA file to guide alignment.

c. Use a non-redundant tRNA reference set containing one representative per isoacceptor (e.g., 42 sequences for S. cerevisiae, with ≥5% sequence divergence).

**3.8.3 Quantification of tRNA Abundance**

a. Count uniquely aligned reads (mapping quality >0, primary alignments).

b. Normalize counts using DESeq2 or similar methods.

c. Differential expression analysis threshold: adjusted P < 0.01 and |log₂ fold change| > 0.6.

**3.8.4 Detection of tRNA Modifications**

a. Calculate basecalling errors (mismatch, insertion, deletion) per nucleotide position using the summed basecalling error frequency: 1 − (frequency of reference base).

b. Generate heat maps of error frequencies across tRNA isoacceptors to visualize modification-associated signatures.

c. For pseudouridine (Ψ) stoichiometry, use NanoRMS or similar supervised algorithms incorporating signal intensity and trace features.

**Protocol 4 circRNA Direct RNA Sequencing Library Preparation**

**Timing**: 2–3 days

This protocol describes the complete workflow for circRNA direct RNA sequencing library construction, including total RNA extraction, rRNA depletion, linear RNA depletion via RNase R treatment, circRNA fragmentation and linearization, adapter ligation, reverse transcription, library preparation, sequencing, and data processing.

**4.1 Total RNA Extraction and Quality Assessment**

**Timing**: 1–2 h

a. Extract total RNA from samples using TRIzol Reagent or a standard RNA extraction kit.

b. **Note**: A minimum input of 5 µg total RNA is recommended to ensure adequate capture of low-abundance circRNAs.

c. Assess RNA integrity using an Agilent Bioanalyzer or TapeStation with the RNA ScreenTape System.

d. **CRITICAL**: RNA Integrity Number (RIN) ≥ 8.5 is recommended to preserve RNA quality and minimize degradation.

**4.2 Ribosomal RNA Depletion**

**Timing**: 1.5–2 h

a. Deplete ribosomal RNA from total RNA using a rRNA depletion kit (e.g., RiboMinus Eukaryote System V2) following the manufacturer's instructions.

b. **Note**: This step reduces the background from abundant rRNA species that would otherwise interfere with circRNA detection.

**4.3 Linear RNA Depletion (RNase R Treatment)**

**Timing**: 2–3 h

a. Take the rRNA-depleted RNA and treat with RNase R to selectively degrade linear RNAs while preserving circular RNAs.

b. Add 5 U of RNase R per microgram of RNA, and incubate the reaction at 37°C for 1 hour.

c. Following RNase R treatment, purify the enriched circRNA fraction using an RNA cleanup kit (e.g., RNeasy MinElute Cleanup Kit or Zymo RNA Clean and Concentrator-5) according to the manufacturer's instructions.

d. **Note**: Optionally, a secondary RNase R treatment can be performed to further remove residual linear RNAs and ensure high-purity circRNA enrichment.

e. Quantify the enriched circRNA using a Qubit RNA HS Assay, and assess RNA quality using an Agilent 2100 Bioanalyzer or TapeStation.

**4.4 CircRNA Fragmentation and Linearization**

**Timing**: 1–1.5 h

a. Since circular RNAs lack free ends required for adapter ligation, subject the purified circRNAs to controlled fragmentation to generate linearized RNA fragments with accessible termini.

b. Fragmentation can be achieved using chemical fragmentation (e.g., divalent cation buffer at elevated temperature) or physical shearing methods.

c. Optimize fragmentation conditions to obtain RNA fragments of appropriate length (typically 200–1000 nt) for nanopore sequencing.

d. Purify the fragmented RNA using RNA Clean XP beads or a column-based cleanup kit to remove short fragments and reaction components.

**4.5 Adapter Ligation and Reverse Transcription**

**Timing**: 4–5 h (overnight incubation optional)

**4.5.1 3′ Adapter Ligation**

**Timing**: 2 h (or overnight at 4°C)

a. Ligate a double-stranded RNA adapter to the 3′ ends of the linearized RNA fragments using T4 RNA Ligase 2.

b. Prepare the ligation reaction in a total volume of 20–50 µL according to Supplemental Table 12.

c. Incubate the reaction at room temperature for 2 hours or at 4°C overnight for optimal ligation efficiency.

d. Purify the ligated RNA using 2 volumes of AMPure RNAClean XP beads, wash twice with 70% ethanol, and elute in nuclease-free water.

Supplemental Table 12 3′ Adapter Ligation Reaction System for circRNA

| **Component** | **Final concentration/amount** |
| --- | --- |
| Linearized circRNA fragments | Variable |
| 20% PEG 8000 | 20% |
| 1× T4 RNA Ligase 2 Buffer | 1× |
| Recombinant T4 RNA Ligase 2 (6 mg/mL) | 4 µL |
| RNase Inhibitor | 1 µL |
| **Total volume** | 20–50 µL |

**4.5.2 Reverse Transcription Adapter (RTA) Ligation**

**Timing**: 30 min

a. Anneal the ONT RTA oligo A and oligo B (or custom DNA oligos with complementary sequences) by heating to 75°C for 15 seconds and cooling to 25°C at 0.1°C per second.

b. Combine the 3′ adapter-ligated RNA with pre-annealed RTA adapter at a 1:2 molar ratio in a 0.2 mL PCR tube.

c. Prepare the ligation reaction mix according to Supplemental Table 13, to a total volume of 15 µL.

d. Mix gently by pipetting, and incubate the reaction at room temperature for 30 minutes.

Supplemental Table 13 RTA Ligation Reaction System for circRNA

| Component | Volume |
| --- | --- |
| 3′ adapter-ligated RNA + pre-annealed RTA adapter | Variable |
| 1× Quick Ligation Reaction Buffer | Variable (final 1×) |
| T4 DNA Ligase (2,000,000 U/mL) | 1.5 µL |
| RNase Inhibitor | 0.5 µL |
| **Total volume** | **15 µL** |

**4.5.3 Reverse Transcription**

**Timing**: 1 h 10 min

a. Prepare the reverse transcription master mix per sample according to Supplemental Table 14.

b. Add the master mix directly to the RTA ligation reaction from Step 5.2, and mix gently by pipetting.

c. Perform the reaction in a thermal cycler with the following program: 60 °C for 1 hour, 85 °C for 5 minutes, then hold at 4 °C.

d. Purify the cDNA-RNA hybrid using 2 volumes of AMPure RNAClean XP beads, wash twice with 70% ethanol, and elute in 8.5 µL nuclease-free water.

Supplemental Table 14 Reverse Transcription Master Mix for circRNA

| **Component** | **Volume per reaction** |
| --- | --- |
| Nuclease-free water | 13 µL |
| 10 mM dNTPs | 2 µL |
| 5× Maxima H Minus Reverse Transcriptase Buffer | 8 µL |
| Maxima H Minus Reverse Transcriptase | 2 µL |
| **Total volume** | **25 µL** |

**4.6 Sequencing Adapter Ligation and Final Library Preparation**

**Timing**: 1.5 h

a. Combine the purified cDNA-RNA hybrid from Step 5.3 with the reaction components according to Supplemental Table 15, to a total volume of 40 µL.

b. Mix gently by pipetting, and incubate the reaction at room temperature for 30 minutes.

c. Add 2 volumes of AMPure RNAClean XP beads to the reaction product, mix thoroughly.

d. Incubate the tube on a rotator mixer for 10 minutes at room temperature.

e. Pellet the beads on a magnetic rack, wash twice with 150 µL Wash Buffer (WSB), resuspending the beads by flicking the tube each time.

f. Elute the final library in 13 µL RNA Elution Buffer (REB), and incubate at room temperature for 10 minutes.

g. Transfer the eluate to a clean tube. Prepare the final library for loading by adding nuclease-free water and RNase-free resuspension buffer (RRB) according to the ONT protocol.

Supplemental Table 15 Sequencing Adapter Ligation Reaction System for circRNA

| Component | Volume |
| --- | --- |
| Purified cDNA-RNA hybrid | Variable |
| 1× Quick Ligation Reaction Buffer | Variable (final 1×) |
| T4 DNA Ligase (2,000,000 U/mL) | 3 µL |
| RMX sequencing adapters (from ONT SQK-RNA002/004 kit) | 6 µL |
| **Total volume** | **40 µL** |

**4.7 Flow Cell Priming and Sequencing**

**Timing**: 30–40 min

a. Use an RNA-specific flow cell (FLO-MIN004RA/FLO-PRO004RA). Allow the flow cell to equilibrate at room temperature for 20 minutes.

b. Perform a flow cell check to assess pore count.

c. Prepare the priming mix according to the ONT Direct RNA Sequencing Kit protocol.

d. Load the priming mix via the priming port, and wait 5 minutes.

e. Mix the final library gently by pipetting, and load the library dropwise into the SpotON sample port.

f. Install the light shield and close the device lid.

g. Configure the MinKNOW software with the following settings:

- Flow cell type: FLO-MIN004RA/FLO-PRO004RA

- Kit selection: Direct RNA Sequencing Kit (SQK-RNA004)

- Disable live basecalling to preserve raw signal data

- Enable saving of .POD5 file format

**4.8 Data Processing and Analysis**

**Timing**: Variable (depending on sequencing data volume)

**4.8.1 Basecalling and Read Processing**

a. Basecall raw .POD5 files using Dorado basecaller with high-accuracy (hac) mode.

b. Convert all uracils (U) to thymines (T) in the output FASTQ files.

**4.8.2 CircRNA Identification**

a. Align reads to the reference genome using minimap2 with parameters optimized for long-read alignment.

b. Identify back-splice junction (BSJ) reads: reads that contain a splice junction where a downstream 5′ splice site is joined to an upstream 3′ splice site.

c. Use tools such as CIRI-long or CIRCexplorer for circRNA detection from nanopore reads.

d. **Note**: Require at least two unique BSJ reads to support circRNA identification.

**4.8.3 RNA Modification Detection**

a. For m^6^A and other modification profiling, use computational tools such as CircRM, xPore, or ELIGOS that analyze basecalling error patterns and signal intensity deviations.

b. Calculate modification stoichiometry at single-base resolution across circRNA transcripts.

c. Compare modification patterns between circRNAs and their linear counterparts to identify RNA-type-specific modification signatures.

d. **Note**: Modifications such as m^6^A in circRNAs are often enriched near back-splice junctions and donor/acceptor splice sites.

**4.9 Quality Control Checkpoints**

The following QC checkpoints are implemented throughout the experimental workflow:

- RNA integrity: RIN ≥ 8.5 assessed by Bioanalyzer or TapeStation.
- RNase R treatment efficiency: Verify enrichment of circRNAs by comparing treated vs. untreated samples using qPCR or Bioanalyzer.
- Library quantification: Final library concentration should be sufficient for sequencing (typically >20 ng).
- Sequencing yield: Monitor real-time pore occupancy and read length distribution during the sequencing run.

**Protocol 5 miRNA Direct RNA Sequencing Library Preparation**

**Timing**: 2–3 days

This protocol describes the complete workflow for miRNA direct RNA sequencing library construction, including total RNA extraction, two-step small RNA/miRNA enrichment, custom adapter ligation, optional reverse transcription, library preparation, sequencing, and data processing.

**5.1 Total RNA Extraction and Quality Assessment**

**Timing**: 1–2 h

a. Extract total RNA from samples using TRIzol Reagent or a standard RNA extraction kit.

b. **Note**: A minimum input of 5 µg total RNA is recommended to ensure adequate capture of low-abundance miRNAs.

c. Assess RNA purity using a NanoDrop spectrophotometer. Acceptable purity criteria: A260/280 ratio between 1.9 and 2.2, and A260/230 ratio ≥ 2.0.

d. Assess RNA integrity using an Agilent Bioanalyzer or TapeStation with the Small RNA ScreenTape System to evaluate miRNA size distribution and exclude heavily degraded samples.

**5.2 Small RNA Enrichment (First-Step Enrichment)**

**Timing**: 1.5–2 h

a. Enrich RNA molecules ≤ 200 nt to deplete long transcripts and concentrate the small RNA fraction.

b. Use a commercial small RNA enrichment kit (e.g., Zymo RNA Clean and Concentrator-5 or miRNeasy Mini Kit) following the manufacturer's instructions.

c. Elute the small RNA fraction in nuclease-free water, and quantify using a Qubit RNA HS Assay Kit.

**5.3 miRNA-Specific Enrichment (Second-Step Enrichment)**

**Timing**: 18–20 h

a. Design biotinylated probes complementary to conserved regions of target miRNA families or to all known miRNAs in the organism of interest.

b. Hybridize the biotinylated probes to the small RNA fraction according to optimized hybridization conditions (e.g., 16 hours at 37 °C with gentle rotation).

c. Capture probe-bound miRNA using streptavidin-coated magnetic beads. Incubate at room temperature for 30 minutes with gentle mixing.

d. Wash the beads thoroughly with wash buffer to remove non-specifically bound RNAs.

e. Elute the enriched miRNA fraction in nuclease-free water.

f. **Note**: This two-step enrichment strategy can increase the miRNA fraction from < 0.1% in the small RNA fraction to > 50% in the enriched pool.

g. Quantify the enriched miRNA using a Qubit RNA HS Assay Kit.

**5.4 Custom Adapter Design and Ligation**

**Timing**: 12–18 h

**5.4.1 Adapter Design**

Design customized single-end adapters specifically for short inserts:

- The 5′ end carries a barcode for multiplexing;
- The 3′ end contains a short sequence complementary to the miRNA 3′ terminus;
- This design reduces adapter dimer formation and non-specific ligation.

**5.4.2 Adapter Ligation**

a. Combine the enriched miRNA with the custom-designed adapters at an optimized molar ratio in a 0.2 mL PCR tube.

b. Prepare the ligation reaction mix according to Supplemental Table 16, to a total volume of 20–50 µL.

c. Mix gently by pipetting, and extend the ligation reaction to 12–16 hours at 4°C to maximize ligation efficiency.

d. **Note**: Optionally, supplement the reaction with ligase enhancers to improve ligation efficiency, achieving efficiencies above 70%.

e. Purify the ligated product using 2 volumes of AMPure RNAClean XP beads, wash twice with 70% ethanol, and elute in nuclease-free water.

Supplemental Table 16 Adapter Ligation Reaction System for miRNA

| **Component** | **Final concentration/amount** |
| --- | --- |
| Enriched miRNA + custom adapters | Variable |
| 20% PEG 8000 | 20% |
| 1× T4 RNA Ligase Buffer | 1× |
| T4 RNA Ligase 2 | Variable |
| RNase Inhibitor | 1 µL |
| **Total volume** | 20–50 µL |

**5.5 Optional Reverse Transcription for Sample Stabilization**

**Timing**: 1 h 10 min

a. For degraded or clinical samples where RNA stability is a concern, an optional reverse transcription step can be introduced to generate cDNA-RNA hybrids, stabilizing miRNA-derived molecules.

b. Prepare a reverse transcription master mix containing: nuclease-free water, 10 mM dNTPs, 5× reverse transcription buffer, and reverse transcriptase (e.g., Maxima H Minus or SuperScript IV).

c. Add the master mix to the adapter-ligated miRNA, and incubate at 60 °C for 1 hour, then 85 °C for 5 minutes.

d. Purify the cDNA-RNA hybrid using 2 volumes of AMPure RNAClean XP beads.

**5.6 Sequencing Adapter Ligation and Final Library Preparation**

**Timing**: 1.5 h

a. Combine the adapter-ligated miRNA (or cDNA-RNA hybrid) with the reaction components according to Supplemental Table 17, to a total volume of 40 µL.

b. Mix gently by pipetting, and incubate the reaction at room temperature for 30 minutes.

c. Add 2 volumes of AMPure RNAClean XP beads to the reaction product, mix thoroughly.

d. Incubate the tube on a rotator mixer for 10 minutes at room temperature.

e. Pellet the beads on a magnetic rack, wash twice with 150 µL Wash Buffer (WSB), resuspending the beads by flicking the tube each time.

f. Elute the final library in 13 µL RNA Elution Buffer (REB), and incubate at room temperature for 10 minutes.

g. Transfer the eluate to a clean tube. Prepare the final library for loading by adding nuclease-free water and RNase-free resuspension buffer (RRB) according to the ONT protocol.

Supplemental Table 17 Sequencing Adapter Ligation Reaction System for miRNA

| Component | Volume |
| --- | --- |
| Adapter-ligated miRNA (or cDNA-RNA hybrid) | Variable |
| 1× Quick Ligation Reaction Buffer | Variable (final 1×) |
| T4 DNA Ligase | Variable |
| RMX sequencing adapters (from ONT SQK-RNA002/004 kit) | 6 µL |
| **Total volume** | **40 µL** |

**5.7 MinKNOW Configuration for Short RNA Sequencing**

**Timing**: 10 min

**CRITICAL**: Reconfigure basecaller thresholds to prevent miRNA reads from being discarded as artifacts, with the following key settings:

- Lower the minimal read length retention cutoff to approximately 15 nt.
- Adjust adapter detection thresholds to accommodate short RNA molecules.
- Disable live basecalling to preserve raw signal data for downstream reprocessing.
- Enable saving of .POD5 file format.

**5.8 Flow Cell Priming and Sequencing**

**Timing**: 30–40 min

a. Use an RNA-specific flow cell (FLO-MIN004RA/FLO-PRO004RA). Allow the flow cell to equilibrate at room temperature for 20 minutes.

b. Perform a flow cell check to assess pore count.

c. Prepare the priming mix according to the ONT Direct RNA Sequencing Kit protocol.

d. Load the priming mix via the priming port, and wait 5 minutes.

e. Mix the final library gently by pipetting, and load the library dropwise into the SpotON sample port.

f. Install the light shield and close the device lid.

g. In the MinKNOW software, select:

- Flow cell type: FLO-MIN004RA/FLO-PRO004RA

- Kit selection: Direct RNA Sequencing Kit (SQK-RNA004)

- Apply the custom short-read configuration settings described in Step 7.

**5.9 Data Processing and Analysis**

**Timing**: Variable (depending on sequencing data volume)

**5.9.1 Basecalling and Read Filtering**

a. Basecall raw .POD5 files using Dorado basecaller with high-accuracy (hac) mode.

b. Apply the adjusted minimal read length filter (approximately 15 nt) to retain miRNA reads.

c. Convert all uracils (U) to thymines (T) in the output FASTQ files.

**5.9.2 Read Alignment and Quantification**

a. Align reads to the miRNA reference database (e.g., miRBase) using a short-read aligner such as BWA-MEM with parameters optimized for short sequences.

b. Use miRNA-oriented mapping pipelines that take into account the short length and high sequence similarity of miRNAs.

c. Incorporate secondary structure information where appropriate to better distinguish homologous family members.

d. Count uniquely aligned reads for each miRNA.

e. Normalize counts and perform differential expression analysis using DESeq2 or similar methods.

**5.9.3 Modification Detection**

For m^6^A and other base modification profiling, analyze basecalling error patterns and signal intensity deviations using computational tools such as xPore, Nanocompore, or ELIGOS.

**5.10 Quality Control Checkpoints**

The following QC checkpoints are implemented throughout the experimental workflow:

- RNA purity: A260/280 = 1.9–2.2, A260/230 ≥ 2.0.
- miRNA size distribution: Verify enrichment of 18–22 nt species by Bioanalyzer or TapeStation.
- Small RNA enrichment efficiency: Confirm depletion of long transcripts (>200 nt).
- miRNA-specific enrichment efficiency: Verify increased proportion of target miRNAs by qPCR.
- Ligation efficiency: Aim for ≥70% ligation efficiency for adapter attachment.
- Library quantification: Final library concentration should be sufficient for sequencing (typically >20 ng).
- MinKNOW configuration: Confirm that minimal read length cutoff has been reduced to approximately 15 nt.

**Protocol 6 Non-poly(A) RNA Direct RNA Sequencing Library Preparation**

**Timing**: 1–2 days

This protocol describes the complete workflow for non-poly(A) RNA direct RNA sequencing library construction, including total RNA extraction, PNK treatment, RNA size fractionation, polyadenylation of short RNA fractions, library construction, sequencing, and data processing.

**6.1 Total RNA Extraction and Quality Assessment**

**Timing**: 1–2 h

a. Extract total RNA from samples using TRIzol Reagent or a standard RNA extraction kit.

b. **Note**: A minimum input of 3 µg total RNA is recommended to ensure adequate capture of non-poly(A) transcripts.

c. Assess RNA integrity using an Agilent Bioanalyzer or TapeStation with the RNA ScreenTape System.

d. **CRITICAL**: RNA Integrity Number (RIN) ≥ 8.0 is recommended to preserve RNA quality and minimize degradation.

e. Assess RNA purity using a NanoDrop spectrophotometer. Acceptable purity criteria: A260/280 ratio between 1.9 and 2.2, and A260/230 ratio ≥ 2.0.

**6.2 Polynucleotide Kinase (PNK) Treatment**

**Timing**: 45 min

a. Treat total RNA with T4 Polynucleotide Kinase (PNK) to convert 5′-hydroxyl and 5′-phosphate ends to 5′-phosphate, enabling efficient adapter ligation in downstream steps.

b. Incubate the reaction at 37°C for 30 minutes according to the manufacturer's instructions.

c. Purify the PNK-treated RNA using an RNA cleanup kit (e.g., Zymo RNA Clean and Concentrator-5), and elute in nuclease-free water.

**6.3 Size Fractionation: Separation of Short and Long RNA Fractions**

**Timing**: 1–1.5 h

a. Use a column-based size enrichment approach to separate PNK-treated total RNA into two fractions: short RNA fraction (approximately <200 nt) and long RNA fraction (approximately >200 nt).

b. Apply the RNA sample to a size-selection spin column. Collect the flow-through containing short RNAs, and retain the column-bound long RNAs.

c. Elute the long RNA fraction from the column with nuclease-free water.

d. **Note**: This fractionation step serves two purposes: (1) it enables specific polyadenylation of the short fraction to facilitate subsequent adapter ligation, and (2) it allows exclusion of full-length rRNAs (which remain in the long fraction) to reduce background.

**6.4 Polyadenylation of the Short RNA Fraction**

**Timing**: 40 min

a. Take the short RNA fraction (approximately < 200 nt) and subject it to polyadenylation using E. coli Poly(A) Polymerase.

b. Prepare the 20 µL polyadenylation reaction mix according to Supplemental Table 18.

c. Incubate the reaction at 37 °C for 30 minutes.

d. **Note**: This polyadenylation step adds a poly(A) tail to the short non-poly(A) RNAs (including snoRNAs, snRNAs, tRNAs, and other small ncRNAs), enabling them to form canonical base-pairing with the poly(T) sequencing adapter in subsequent steps.

e. After incubation, inactivate the poly(A) polymerase by heating at 65°C for 10 minutes.

Supplemental Table 18 Polyadenylation Reaction System for Short Non-poly(A) RNA

| **Component** | **Volume** |
| --- | --- |
| Short RNA fraction (up to 1 µg) | Variable |
| 10× Poly(A) Polymerase Reaction Buffer | 2 µL |
| 10 mM ATP | 2 µL |
| E. coli Poly(A) Polymerase (5 units) | 1 µL |
| Murine RNase Inhibitor (optional) | 0.5 µL |
| Nuclease-free water | Variable (adjust to final 20 µL) |
| **Total volume** | **20 µL** |

**6.5 Re-pooling of RNA Fractions**

**Timing**: 10 min

a. Combine the polyadenylated short RNA fraction with the untreated long RNA fraction (which contains mRNAs and other naturally polyadenylated transcripts).

b. **Note**: The re-pooled sample contains all initial RNAs, with the short non-poly(A) fraction now artificially polyadenylated to enable capture.

**6.6 Optional: Ribosomal RNA Depletion**

**Timing**: 1.5–2 h

a. If rRNA depletion is desired to further reduce background, perform rRNA removal using biotinylated probes targeting conserved regions of rRNA.

b. Hybridize probes to the RNA sample, then remove probe-rRNA complexes with streptavidin-coated magnetic beads.

c. **Note**: This step can achieve ≥ 95% rRNA depletion while minimizing mechanical fragmentation of non-poly(A) species.

**6.7 Reverse Transcription Using High-Temperature, Strand-Displacing Polymerase**

**Timing**: 1.5–2 h

**6.7.1 Rationale**

Standard direct RNA-seq protocols use reverse transcriptases with temperature optima between 50-70°C, which exhibits strong strand displacement activity and functions at temperatures up to 70°C, facilitating RNA unfolding and enabling full-length coverage of structured ncRNAs.

**6.7.2 Priming and First-Strand Synthesis**

a. To the re-pooled RNA sample, add random primers to enable initial "coating" of RNAs with protective cDNA strand fragments along their entire length.

b. Incubate at 50 °C for 10 minutes to allow to initiate first-strand synthesis from random primers.

c. **Note**: Due to the strong strand displacement activity of reverse transcriptases, these short first-strand fragments are subsequently displaced by the cDNA strand initiated from the poly(T) adapter.

**6.7.3 Full-Length Reverse Transcription**

a. Add the poly(T)-containing reverse transcription adapter (RTA) to the reaction.

b. Incubate at 60-70 °C for 1 hour to facilitate reverse transcription of structured RNA regions.

c. Inactivate the enzyme by heating at 85°C for 5 minutes.

d. **Note**: Alternative high-temperature, strand-displacing enzymes such as Omniamp (now discontinued) may also be suitable; Lavalamp has shown reduced performance for this application.

**6.8 Sequencing Adapter Ligation and Final Library Preparation**

**Timing**: 1.5 h

a. Combine the purified cDNA-RNA hybrid from Step 7 with the reaction components according to Supplemental Table 19, to a total volume of 40 µL.

b. Mix gently by pipetting, and incubate the reaction at room temperature for 30 minutes.

c. Add 2 volumes of AMPure RNAClean XP beads to the reaction product, mix thoroughly.

d. Incubate the tube on a rotator mixer for 10 minutes at room temperature.

e. Pellet the beads on a magnetic rack, wash twice with 150 µL Wash Buffer, resuspending the beads by flicking the tube each time.

f. Elute the final library in 13 µL RNA Elution Buffer (REB), and incubate at room temperature for 10 minutes.

g. Transfer the eluate to a clean tube. Prepare the final library for loading by adding nuclease-free water and RNase-free resuspension buffer (RRB) according to the ONT protocol.

Supplemental Table 19 Sequencing Adapter Ligation Reaction System for Non-poly(A) RNA

| Component | Volume |
| --- | --- |
| Purified cDNA-RNA hybrid | Variable |
| 1× Quick Ligation Reaction Buffer | Variable (final 1×) |
| T4 DNA Ligase (2,000,000 U/mL) | 3 µL |
| RMX sequencing adapters (from ONT SQK-RNA004 kit) | 6 µL |
| **Total volume** | **40 µL** |

**6.9 Flow Cell Priming and Sequencing**

**Timing**: 30–40 min

a. Use an RNA-specific flow cell (FLO-MIN004RA/FLO-PRO004RA). Allow the flow cell to equilibrate at room temperature for 20 minutes.

b. Perform a flow cell check to assess pore count.

c. Prepare the priming mix according to the ONT Direct RNA Sequencing Kit (SQK-RNA004) protocol.

d. Load the priming mix via the priming port, and wait 5 minutes.

e. Mix the final library gently by pipetting, and load the library dropwise into the SpotON sample port.

f. Install the light shield and close the device lid.

g. In the MinKNOW software, select:

- Flow cell type: FLO-MIN004RA/FLO-PRO004RA

- Kit selection: Direct RNA Sequencing Kit (SQK-RNA004)

- Enable real-time basecalling or disable to preserve raw signal data

- Enable saving of .POD5 file format

**6.10 Alternative: Sequence-Specific Non-poly(A) RNA Enrichment**

For targeted sequencing of specific non-poly(A) RNAs (e.g., 16S rRNA), a custom reverse transcription adapter (RTA) can be designed with the following workflow:

a. Order custom oligo A and a modified version of oligo B where the 10(T) sequence is replaced with 10 bases complementary to the 3′ end of the target RNA sequence:

- Oligo A (5′→3′): /5PHOS/GGCTTCTTCTTGCTCTTAGGTAGTAGGTTC

- Modified Oligo B (5′→3′): GAGGCGAGCGGTCAATTTTCCTAAGAGCAAGAAGAAGCC(NNNNNNNNNN)

- *Note*: NNNNNNNNNN represents the 10-base sequence complementary to the target RNA 3′ end.

b. Anneal oligo A and modified oligo B at a 1:1 ratio in annealing buffer (10 mM Tris-HCl, 50 mM NaCl, pH 7.5) by heating to 95 °C for 2 minutes and cooling slowly (0.1 °C/sec) to 25 °C.

c. This custom RTA functions as the reverse transcription primer, enabling specific capture and sequencing of the target non-poly(A) RNA.

**6.11 Data Processing and Analysis**

**Timing**: Variable (depending on sequencing data volume)

**6.11.1 Basecalling and Read Processing**

a. Basecall raw .POD5 files using Guppy, Dorado, or the latest basecaller with high-accuracy (hac) or super-accuracy (sup) mode.

b. For RNA modification detection, utilize the latest Dorado basecalling models which support simultaneous detection of multiple modification types.

c. Convert all uracils (U) to thymines (T) in the output FASTQ files as needed for alignment.

**6.11.2 RNA Modification Detection**

Using the Dorado basecaller with the latest models (e.g., Dorado V5), the following RNA modifications can be detected at single-base resolution:

- m^6^A (N6-methyladenosine)
- m^5^C (5-methylcytosine)
- Pseudouridine (Ψ)
- Inosine (I)

*Note*: Modification detection accuracy is assessed using ONT official benchmarks.

**6.11.3 Read Alignment and Quantification**

a. Align reads to the reference genome or transcriptome using minimap2 with parameters optimized for long-read alignment (-ax map-ont).

b. For ncRNA quantification, use specialized references containing sequences of snoRNAs, snRNAs, tRNAs, scRNAs, srpRNAs, and rRFs.

c. Count uniquely aligned reads for each RNA species.

d. Normalize counts and perform differential expression analysis using DESeq2 or similar methods.

**6.12 Quality Control Checkpoints**

The following QC checkpoints are implemented throughout the experimental workflow:

- RNA integrity: RIN ≥ 8.0 assessed by Bioanalyzer or TapeStation.
- RNA purity: A260/280 = 1.9-2.2, A260/230 ≥ 2.0.
- Size fractionation efficiency: Verify separation of short (<200 nt) and long (>200 nt) RNA fractions by Bioanalyzer.
- Polyadenylation efficiency: Confirm successful poly(A) tail addition to short ncRNAs by qPCR or Bioanalyzer.
- rRNA depletion efficiency: Verify ≥95% rRNA removal when performed.
- Reverse transcription efficiency: Assess cDNA-RNA hybrid yield and length distribution.
- Library quantification: Final library concentration should be sufficient for sequencing (typically >20 ng).

**EXPECTED OUTCOMES**

1. **mRNA DRS**: This protocol will generate high-quality direct RNA sequencing libraries for mRNA, enabling full-length transcript sequencing, isoform analysis, and native RNA modification detection (e.g., m^6^A) with a final library yield >30 ng.
2. **rRNA DRS**: This protocol enables direct sequencing of full-length rRNA molecules, supporting detection of diverse rRNA modifications (m⁷G, Ψ, etc.) and characterization of modification dynamics under stress conditions, with a final library yield >30 ng.
3. **tRNA DRS**: This protocol will generate tRNA sequencing libraries with efficient adapter ligation (>70% efficiency), enabling full-length tRNA sequencing, abundance quantification, and modification mapping at single-base resolution, with a minimum input of 250 ng deacylated tRNA.
4. **circRNA DRS**: This protocol enables efficient enrichment of circRNAs via RNase R treatment, supporting full-length circRNA sequencing, back-splice junction identification, and circRNA-specific modification profiling, with at least two unique BSJ reads supporting each circRNA identification.
5. **miRNA DRS**: This protocol enables efficient enrichment and sequencing of short miRNA molecules, with custom MinKNOW settings retaining reads as short as 15 nt, supporting miRNA quantification, differential expression analysis, and base modification detection.
6. **Non-poly(A) RNA DRS**: This protocol enables simultaneous sequencing of both polyadenylated and non-polyadenylated RNA species, including snoRNAs, snRNAs, and other structured ncRNAs, supporting full-length transcript sequencing and multi-type RNA modification detection at single-base resolution.

**QUALITY CONTROL CHECKPOINTS SUMMARY**

| **Experimental Stage** | **QC Criterion** | **Acceptable Threshold** |
| --- | --- | --- |
| Total RNA extraction | RNA integrity (RIN) | ≥8.0 (≥8.5 recommended for mRNA, rRNA, circRNA) |
| Total RNA extraction | RNA purity (A260/280) | 1.9–2.2 |
| Total RNA extraction | RNA purity (A260/230) | ≥2.0 |
| RNA enrichment/pretreatment | Target RNA enrichment efficiency | >50% for miRNA-specific enrichment; ≥95% for rRNA depletion |
| Adapter ligation | Ligation efficiency | ≥70% for tRNA and miRNA adapter ligation |
| Final library preparation | Library concentration | >20 ng (target >30 ng for mRNA and rRNA) |
| Sequencing run | Flow cell pore count | Meet ONT official sequencing requirements |
| Sequencing run | Pore occupancy | Real-time monitoring within normal range |
| Data processing | Unique aligned reads | Mapping quality >0 for primary alignments |
